# Operant House: A versatile open-source platform for automated operant conditioning testing of mice in home cages

**DOI:** 10.1371/journal.pone.0360626

**Published:** 2026-10-13

**Authors:** Shintaro Otsuka, Yuriko Nakamura, Aya Ito-Ishida, Kunimichi Suzuki, Ayako Ishikawa, Keiko Matsuda, Shigetomo Suyama, Anis Contractor, Michisuke Yuzaki

**Affiliations:** 1 Department of Physiology, Keio University School of Medicine, Shinjuku, Tokyo, Japan; 2 Department of Neuroscience, Northwestern University Feinberg School of Medicine, Evanston, Illinois, United States of America; 3 Laboratory for Brain Development and Disorders, RIKEN Center for Brain Science, Wako, Saitama, Japan; 4 Center for Human biology-Microbiota-Quantum computational Research, Keio University, Shinjuku, Tokyo, Japan; Universidad de Guadalajara, MEXICO

## Abstract

Operant conditioning is a powerful method for probing cognitive functions, yet its widespread use is constrained by low throughput, labor-intensive procedures, and high costs. Here, we developed “Operant House,” a low-cost, programmable device equipped with a touchscreen, retractable levers, and a water-reward port, designed to enable automated operant conditioning with minimal experimenter intervention in individually housed mice within their home cages. Although individual home-cage testing requires careful attention to animal welfare, the system permits continuous monitoring through live camera observation and recorded videos. As proof of concept, we implemented two cognitive protocols: a delayed non-match-to-position (DNMTP) test for assessing working memory and a two-choice spatial discrimination (TCSD) test involving working-memory-related processes and behavioral flexibility. Because working memory impairment is one of the earliest clinical features of Alzheimer’s disease (AD), we further applied these protocols to a knock-in mouse model carrying familial AD-associated amyloid precursor protein mutations (*App*^*NL-G-F*^ mice). Using this model, we detected significant working memory deficits as early as 5 months of age. At this age, we also detected subtle and spatially heterogeneous synaptic alterations in the prefrontal cortex, whereas conventional electrophysiological measures were largely preserved. Together, these findings highlight the Operant House as a cost-effective, scalable platform for assessing higher cognitive functions in mice, offering an accessible tool for investigating neurological and neuropsychiatric disease models.

## Introduction

Operant conditioning has long been utilized in behavioral neuroscience to evaluate cognitive performance in a variety of species. In these tests, subjects are trained to perform specific behaviors, such as touching panels or pressing levers, in order to obtain rewards, such as water and food. Well-established protocols have been developed to measure various aspects of cognitive functions, including visual and spatial recognition, attention, impulsivity, working memory, and flexibility [1–5]. Operant conditioning serves as an unbiased assay, providing valuable insights into mouse cognition within the context of various neuropsychiatric and neurological disease models, including schizophrenia, autism spectrum disorders, and Alzheimer’s disease (AD). Conventional operant conditioning is typically conducted individually in a specialized apparatus under the supervision of an experimenter [6]. This process is labor-intensive and inefficient, as it must be repeated for each animal in a large cohort. Additionally, removing animals from their home cages—often during their inactive daytime period—for daily training sessions can induce significant stress and impaired performance. These limitations present substantial challenges to the widespread application of operant conditioning in disease model animals (Fig 1A).

**Fig 1:**
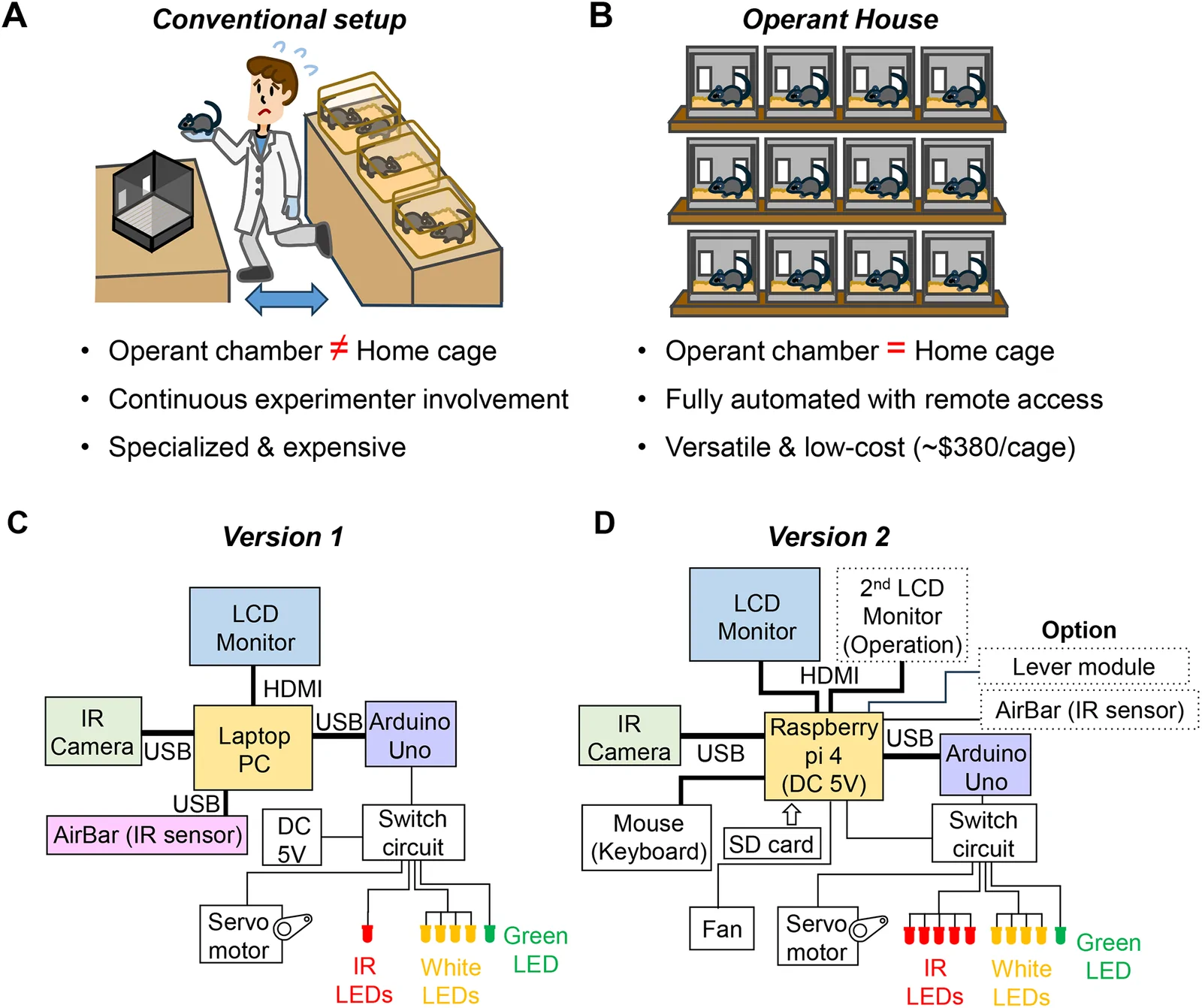
Concept and the diagram of the Operant House. (**A, B**) Concept. In the conventional setup (**A**), animals must be transferred between their home cages and a dedicated operant conditioning chamber by an experimenter. In contrast, in the low-cost Operant House setup (**B**), operant tasks are performed automatically within individual home cages in parallel. (**C, D**) Block diagrams. To capture IR camera images and control peripheral devices, version 1 (**C**) uses a laptop PC, whereas version 2 (**D**) employs a single-board computer (Raspberry Pi 4). To detect nose-poke actions, version 1 (**C**) uses a touchscreen equipped with an IR sensor (AirBar) placed on the LCD monitor, while version 2 (**D**) utilizes image recognition of mouse movements captured by an IR camera. Several optional devices are also available, including an operation monitor, lever modules, and an AirBar.

To overcome these challenges, various devices have been developed to enable automated operant conditioning in the home cage, thereby reducing experimenter intervention and allowing training during the animals’ active period. Importantly, Poddar et al. developed the Automated Rodent Training System (ARTS), a low-cost, modular system that enabled fully automated, high-throughput home-cage training of rats in complex sensorimotor tasks [7]. Francis and Kanold subsequently developed the Psibox, a low-cost system designed to fit within conventional mouse cages that enabled automated positive-reinforcement training in the home cage and demonstrated its utility in an auditory detection task [8]. These studies established the feasibility and advantages of scalable, automated operant training in the home-cage environment.

Other approaches have addressed complementary aspects of automated behavioral testing. For example, the CombiCage, which connects a home cage to a commercial operant chamber for the 5-choice serial reaction time task (5-CSRTT), was shown to achieve a tenfold increase in learning speed compared to conventional chambers for operant conditioning [9]. However, conducting simultaneous tests on multiple mice using such equipment is challenging due to its high cost and large size. An alternative approach is the IntelliCage system, which allows a group of individually tagged mice to be housed together in a single large cage for operant conditioning [10–15]. While the IntelliCage provides valuable population-level behavioral information, individual mouse behavior can be influenced by the presence of other mice in the same cage [16–19]. Building on these previous advances, for our intended applications we sought a platform that combined low per-unit cost and scalability with programmable touchscreen-based cognitive tasks, conventional lever-press paradigms, integrated video monitoring, and openly available hardware and software resources, while allowing each animal to be tested automatically in its own home cage.

To meet these requirements, we developed a compact and affordable operant conditioning device, referred to as the “Operant House.” The Operant House is a fully programmable device equipped with a touchscreen and retractable levers for operant tasks, along with a water port for reward delivery, all integrated into an individually housed home cage. Because each device accommodates a single animal throughout the experimental paradigm, individual housing is an inherent limitation of the present design and requires careful attention to animal welfare, including the potential effects of social isolation. The Operant House therefore enables live observation and retrospective inspection of recorded task videos to facilitate monitoring of the animals’ general condition and possible signs of stress. This platform can be easily and inexpensively (US $380–660 per unit), assembled using readily available materials and a 3D printer, following step-by-step instructions provided on our website (https://shintaro18.github.io/). As a result, this affordable platform enables automated behavioral testing with minimal experimenter intervention and parallel scaling of operant conditioning across individually housed mice in their home cages (Fig 1B).

As proof of concept, we present two standard protocols for assessing working memory using Operant House: the delayed non-matching-to-position (DNMTP) test and the two-choice spatial discrimination (TCSD) test. Working memory, the capacity to temporarily maintain goal-relevant information to guide behavior, is frequently impaired across neurological and neuropsychiatric disorders. Notably, working memory deficits associated with prefrontal cortex dysfunction emerge during the preclinical stage of AD [20–22] and in AD model mice such as 3xTg, APP/PS1 and 5xFAD, in which familial AD-related mutations are overexpressed [22–25]. The recently developed *App*^*NL–G–F*^ knock-in mice, which harbor three familial AD mutations in the amyloid precursor protein (*APP*) gene, are considered to more faithfully recapitulate human AD pathology because they lack artifacts caused by protein overexpression [26]. However, their behavioral phenotypes are generally milder than those of overexpression models, and the presence of early working memory deficits has remained inconsistent across studies [26–30]. Thus, using Operant House, we examined early cognitive performance in *App*^*NL–G–F*^ mice using the DNMTP and TCSD tasks. We found deficits in these tasks as early as 5 months of age. These findings indicate that Operant House provides a sensitive, standardized platform for detecting subtle cognitive deficits and may facilitate investigations of cognitive impairment in neurological and neuropsychiatric disorders.

## Materials and methods

### Automated operant conditioning using the Operant House

To begin habituation, mice were transferred from their home cages to the Operant House chamber between 9:00 a.m. and 12:00 p.m. The chamber was illuminated, and water was withheld until 9:00 p.m., resulting in 9–12 h of water restriction. Thereafter, the light was turned off, and mice were given ad libitum access to water until 9:00 a.m. the following day. From the second day onward, animals were subjected to either the DNMTP or TCSD paradigm.

In both paradigms, each daily session began at midnight (0:00), signaled by illumination of a green LED and a brief 1-s water delivery. Sessions continued until the animal obtained a predetermined number of water accesses or until the maximum session duration elapsed. During each session, the cumulative duration of water access was continuously monitored to ensure adequate opportunity for water consumption. If the cumulative water-access duration fell below the predefined level (DNMTP: 160 s; TCSD: 300 s), supplemental water access was provided immediately after the session. Outside of task sessions, mice did not have access to water (Fig 6A, 8A). The actual volume of water consumed by each mouse was not directly measured.

Body weight was monitored as part of the assessment of animal welfare during behavioral testing. To assess whether the DNMTP water-access regimen was associated with systematic changes in body weight, paired body-weight measurements obtained before and after testing were analyzed in six wild-type mice from independent experiments performed using Operant House version 2. These experiments used the same DNMTP water-access regimen (up to 80 water-access periods of 2 s each, corresponding to a maximum programmed water-access duration of 160 s per day), although other experimental parameters were not identical across all experiments. Pre- and post-testing body weights were compared using a paired t-test.

The ceiling light remained off during behavioral sessions and during the dark phase (9:00 p.m. to 9:00 a.m.), except when punishment was delivered.

### Delayed non-matching to position (DNMTP) test

A mask with three apertures, each aligned with an illuminated rectangular panel on the LCD screen, was used to present visual cues. The protocol consisted of three blocks (Fig 5A). Mice were advanced to the next block either after a predetermined number of days or when their performance reached a specified criterion level. Daily trials in each block were concluded when the total number of correct responses reached 80 or when 10 hours had elapsed since the start of the trials.

#### Block 1−1: VD1 task (Fig 5B).

In each trial, one of the three panels was randomly illuminated. When the subject touched the illuminated panel, the panel light was turned off, the green LED at the water port was activated, and the subject was allowed access to water for 2 sec. The water nozzle retracted 2 sec after detecting the subject’s nose poke into the water slit. Touching an unilluminated aperture was considered an incorrect response, resulting in a 2-sec timeout. The inter-trial interval (ITI) was set at 5 sec. Since many subjects did not initially recognize the association between panels and the water reward, they were given 1-sec water access if they did not respond to the illuminated panels for longer than 6 min (stimulus reward). VD1 was performed until the subject achieved 5 successive correct responses, at which point they were moved to visual discrimination task 2 (VD2).

#### Block 1–2: VD2 task (Fig 5C).

VD2 differed from VD1 in two ways. Firstly, the stimulus reward was not provided. Secondly, mice were punished with chamber light illumination (10 sec) when touching unilluminated panels. In the experiments involving ibotenic acid injection, VD2 was repeated for four days before the DNMTP pre-acquisition task. For experiments with the Alzheimer’s disease model, VD2 was repeated until the correct response exceeded 50%. The learning curves in control animals were similar in both conditions. In the analysis of wild-type animals using Operant House Version 2, VD2 was repeated until the correct response exceeded 65% for two consecutive days.

#### Block 2: DNMTP pre-acquisition task (Fig 5D).

The DNMTP pre-acquisition task was designed to help mice recognize the rules employed in the DNMTP task. At the beginning of the sample phase, either the left or right panel flickered. When the mice touched the flickering panel, the panel turned off. After 1 sec, the middle panel illuminated, signaling the start of the delay phase. This sequence was repeated once more to indicate the end of the delay phase. The delay phase lasted 1 sec, followed by the choice phase. In the choice phase, the panel located opposite the sample panel was illuminated, and touching this panel led to access to a water reward for 2 sec. Touching the same panel that had been illuminated during the sample phase was considered an incorrect response and resulted in a 10-sec chamber illumination punishment. The ITI was 30 sec. The DNMTP pre-acquisition task was performed for one day in Version 1. In Operant House Version 2, the DNMTP pre-acquisition task was repeated until the correct rate exceeded 85% for two consecutive days.

#### Block 3: DNMTP task (Fig 5E, F).

The DNMTP task was similar to the DNMTP pre-acquisition task, with the difference being that in the choice phase, both panels on the left and right sides were illuminated. The subject was required to remember the location of the sample panel and touch the correct panel in the choice phase. During the acquisition trials, the duration of the delay phase was set to 1 sec. The trials were repeated until the total success rate reached 80% (for *App*^*NL-G-F*^ mice) or 75% (for experiments using Operant House Version 2) for two consecutive days. Most wild-type mice achieved this criterion within 1–2 weeks. Once the criterion was met, the duration of the delay was gradually increased (Fig 5F).

To prevent mice from adapting to a fixed pattern of delay changes, two delay conditions (delay A and delay B) were randomly assigned on a trial-by-trial basis. To ensure that the delay schedules were identical across animals, the same randomization seed was used for all mice. Trials assigned to delay A were used for the working memory analysis.

In some experiments, the actual delay duration, defined as the interval between touches of the sample and choice panels, was prolonged by behaviors such as eating or sleeping during the delay period and differed between experimental groups. The primary behavioral analyses therefore included all trials, and additional sensitivity analyses were performed using only trials in which the actual delay duration was below a predefined threshold (programmed delay × 1.3 + 10 s). The same criterion was applied to all groups and was based solely on actual delay duration, irrespective of trial outcome. Because selection based on actual delay duration could itself introduce bias, these delay-restricted analyses were considered sensitivity analyses rather than independent confirmatory tests. The numbers of retained and excluded trials are reported in the corresponding Results sections.

### Two-choice spatial discrimination (TCSD) test

A mask with five apertures, each corresponding to illuminated rectangles on the LCD screen, was used to present visual cues. In the TCSD test, mice were required to memorize the correct position of the illuminated panel from the previous trial to receive a water reward. The experiment consisted of two blocks (Fig 8A). In the first block, mice were trained to associate touching the illuminated panels with the water reward by performing visual discrimination tasks (VD1 and VD2). The ITI was set at 5 sec. Daily trials were terminated either when the total number of correct responses reached 150 or when 6 hours had elapsed since the start of the trials.

#### Block 1−1: VD1 task (Fig 8B).

The VD1 task was conducted similarly to that of the DNMTP test (Fig 5B), with the exception that one of the five panels was randomly illuminated. The stimulus reward was also employed. Once the mice achieved five successive correct responses, they proceeded to the VD2 task.

#### Block 1–2: VD2 task (Fig 8C).

The VD2 task was also identical to the VD2 in DNMTP test, except that five panels were used. The VD2 task was repeated until the correct response rate exceeded 50%.

#### Block 2: TCSD task (Fig 8D).

In the TCSD task, two panels were simultaneously illuminated, and one of the two panels was randomly assigned as the correct choice at the beginning of trials. When the subject touched the correct panel, it was rewarded with 2 sec of water access. The incorrect choice was punished with chamber illumination for 5 sec. The position of the correct choice was reversed to the opposite side when the subject achieved more than 7 correct responses in the most recent 8 trials. The TCSD task was repeated for 3 days.

### Lever-pressing conditioning test (S13 Fig)

Following a 24-hour water-restriction period, the subjects were placed in the chamber at 9:00 p.m. and the task commenced immediately. To begin trials, both right and left levers were inserted into the chamber and pressing either lever resulted in a reward of 2-sec access to water. Daily trials were terminated when the subject pressed the levers 80 times, which was followed by the retraction of the levers. A wire grid wall was inserted in the middle of the chamber on the first day to reduce the chamber space and prompt the mice to press the levers. The wire grid wall was removed on the second day.

### Animals

All procedures involving animal care and use were approved by the Animal Resource Committee of Keio University (approval number 09050−5) and were conducted in accordance with institutional guidelines.

Homozygous *App*^*NL-G-F*^ knock-in mice were obtained from RIKEN Bioresource Center (#RBRC06344) and crossed with C57BL/6J (Crlj) to produce heterozygous breeding pairs. The resulting mice were used to obtain mice homozygous for *App*^*NL-G-F*^, while wild-type littermates were used as control subjects. Up to four littermates were housed in the same cages (155 x 245 x 148 mm) under a 12:12 hour light/dark cycle at a temperature of 22 ± 1°C, with ad libitum access to food and water. During individual housing and behavioral testing in the Operant House, body weight was measured daily and the general condition of each mouse was assessed by visual inspection. Health monitoring included appearance and grooming, posture, spontaneous activity, apparent food and water intake, and signs of injury or distress. Animals showing substantial weight loss, abnormal behavior, or other signs of deteriorating health would have been evaluated promptly and, when appropriate, removed from the experiment in accordance with the approved humane-endpoint criteria. No animals met these exclusion or humane-endpoint criteria during the experiments.

For the DNMTP experiment, both female and male mice at the age of 5 months were used. In the TCSD experiment, we used 5-month-old male mice. Wild-type mice (C57BL/6J, 2 males and 3 females) at the age of 4 months were used for the lever-pressing conditioning test. Histological and electrophysiological analyses were conducted using male mice.

### Ibotenic acid injection

Male wild-type mice (C57BL/6J, Japan SLC) at the age of 2 months were subjected to an intraperitoneal injection of a mixed anesthetic comprising medetomidine (0.3 mg/kg), midazolam (4 mg/kg), and butorphanol (5 mg/kg). Ibotenic acid (Abcam, ab120041) was prepared as a solution at a concentration of 10 mg/ml in saline and stored at −20°C until use. To verify the accuracy of the injection site, Red Retrobeads IX (LumaFluor) were added at a volume ratio of 1:20 to the ibotenic acid solution. A total of 400 nl of the resulting mixture was injected into the bilateral prefrontal cortex using a stereotaxic device (Stoelting) and Nanoliter 200 (World Precision Instrument). The injection coordinates were as follows: anterior-posterior (AP) +2.75 mm, medial-lateral (ML) 0.4 mm, and depth from the pia 0.8 mm. For the control group, saline and Red Retrobeads IX were injected. Following the surgery, the mice were allowed to recover for a period of five days before being subjected to the behavioral task.

### Immunostaining of c-Fos after the DNMTP task

Female wild-type mice (C57BL/6J, Japan SLC) aged 2–3 months were trained in the DNMTP task following the protocol described above. On the day when the mice performed the task with a delay of 8–12 sec, they were perfused with 4% paraformaldehyde (PFA) in phosphate-buffered saline (PBS) 60–90 min after the task began. Control mice were housed individually and were also perfused on the same day. The brains were post-fixed overnight with 4% PFA, and 100 μm sections were obtained using a vibratome (DTK-1000, Dosaka).

The brain slices were treated with a blocking solution containing 2% donkey serum and 0.3% Triton-X, and then incubated with an anti-c-Fos antibody (mouse monoclonal [2H2], Abcam, ab208942, 1:1000) dissolved in the same blocking solution. After three washes with PBS, the sections were incubated with the secondary antibody (donkey anti-mouse Alexa 555, Invitrogen, 1:500) and Neurotrace 600/647 (ThermoFisher, 1:500) for three hours at room temperature. Following three additional washes with PBS, the sections were mounted in 70% glycerol, and images were captured using a 10 x objective lens on a confocal microscope (FV1000, Olympus). Projection images from five z-sections were analyzed using Fiji software. Images were first filtered with a median filter, and the density of c-Fos positive cells was quantified. Cells exhibiting c-Fos intensity three times greater than the background were defined as c-Fos positive cells. For each mouse, measurements obtained within each brain region were averaged to yield one mean density value per region for statistical analysis.

### Immunostaining of Aβ and synaptic markers

Acute coronal slices of the medial prefrontal cortex (mPFC) with a thickness of 300 µm were obtained following the same procedure described in the electrophysiology section below. The slices were fixed in 4% PFA in PBS overnight at 4 °C. Cryoprotection was achieved by immersing the slices in a 30% sucrose solution in PBS, followed by embedding them in Tissue-Tek embedding medium (Electron Microscopy Sciences, Hatfield, PA, USA). The frozen samples were then cut into 20 μm-thick coronal sections using a cryostat (CM1850, Leica).

Sections were treated with a blocking solution consisting of 10% donkey serum and 0.1% Triton-X in PBS for 30 min and incubated overnight with primary antibodies in 0.1% Triton-X / PBS. The following primary antibodies were used: anti-VGluT1 (rabbit, Frontier Institute, 1:500), anti-VGAT (guinea pig, Frontier Institute, 1:1000), anti-Iba1 (rabbit, Sigma, 1:500), and anti-Aβ antibody (mouse monoclonal [82E1], IBL, 1:500). After three washes with 0.1% Triton-X / PBS, the sections were incubated for three hours at room temperature with the appropriate secondary antibodies (goat anti-mouse IgG1 Alexa 488, goat anti-mouse IgG1 Alexa 647, donkey anti-rabbit Cy3, or donkey anti-guinea pig Alexa 488, Jackson, 1:500) and Neurotrace 600/647 (ThermoFisher, 1:500). Following three additional washes with 0.1% Triton-X / PBS, the sections were mounted in Vectashield Mounting Medium (Vector Laboratories). Images were captured using either a confocal microscope (FV1000, Olympus) with a 4x objective lens or an Olympus SR-OSR microscope with either a 10 x or 100 x objective lens. For images obtained using the 100x objective lens, z-projected images from 23 z-sections were acquired and analyzed using Fiji software. The analysis was performed semi-automatically using a custom macro. Background noise was reduced by applying background subtraction using the rolling ball method (5–50 pixels). Regions of interest corresponding to Aβ plaques were determined based on intensity using Otsu binarization and size thresholding (above 50 μm^2^). The mean signal intensity outside the plaques was obtained from three different positions within areas of 370 μm^2^.

### Electrophysiological recordings in acute cortical slices

Coronal slices of the mPFC with a thickness of 300 µm were prepared from 5-6-month-old mice. The mice were anesthetized with isoflurane, and acute brain slices were obtained using a vibratome (DSK-1000N, Leica) in chilled (0–4 °C) normal artificial cerebrospinal fluid (ACSF) containing (in mM): 126 NaCl, 3 KCl, 1.3 MgSO_4_, 2.4 CaCl_2_, 1.2 NaH_2_PO_4_, 26 NaHCO_3_, and 10 glucose. The slices were maintained at a temperature of 34 °C with 95% O_2_ and 5% CO_2_ during the cutting procedure. Before recording, the slices were incubated for a minimum of 30 min in normal ACSF at 34 °C, which was continuously bubbled with 95% O_2_ and 5% CO_2_.

Pyramidal neurons were identified based on their characteristic pyramidal soma shape using infrared differential interference contrast optics (BX51, Olympus) and a × 60 water immersion objective lens (LUMPlanFI/IR, NA 0.9) along with a CCD camera (Cool SNAP Kino, Teledyne Imaging). Whole-cell patch-clamp recordings were performed on neurons in layer 2/3 and layer 5 of the mPFC, including the anterior cingulate, prelimbic, and infralimbic areas, at room temperature. The patch pipettes had a resistance of 4–6 MΩ and were filled with an intracellular solution consisting (in mM): 130 K-gluconate, 8 KCl, 1 MgCl_2_, 0.6 EGTA, 10 HEPES, 3 MgATP, 0.5 Na_2_GTP, and 10 Na-phosphocreatine (pH 7.3 with KOH).

To assess the intrinsic membrane properties and excitability of pyramidal neurons, 500-ms step currents of varying amplitudes were injected in current-clamp mode. Current injection experiments were performed within 5 min of establishing the whole-cell patch-clamp configuration.

For measuring evoked EPSCs/IPSCs and miniature EPSCs/IPSCs, we used an internal solution containing (mM) 130 Cs-gluconate, 6 KCl, 10 NaCl, 10 HEPES, 0.16 CaCl_2_, 2 MgCl_2_, 0.5 EGTA, 4 Na-ATP, and 0.4 Na-GTP (pH 7.3, adjusted with CsOH). EPSCs and IPSCs were recorded at holding potentials of −70 mV and 0 mV, respectively. To assess the inhibitory-to-excitatory (I/E) balance, EPSCs and IPSCs were evoked by electrical stimulation at 20-sec intervals using a pair of bipolar tungsten electrodes (FHC), positioned near the recorded neuron (S20A Fig). Ten to twenty consecutive traces were averaged to obtain the mean EPSC or IPSC. The baseline current was defined as the average current in a 20-ms window prior to the electrical stimulation, and the peak EPSC and IPSC amplitudes were calculated as the absolute value of the negative or positive peak current relative to the baseline. Miniature EPSCs and IPSCs were recorded in the presence of 1 μM TTX. The experimental setup included a Multiclamp 700B amplifier, Digidata 1440A A/D converter, and pClamp10 software (Molecular Devices). Data were digitized at 20 kHz, low-pass filtered at 4 kHz, and analyzed using custom-made MATLAB scripts (MathWorks). Only recordings from cells with a seal resistance > 1 GΩ and a series resistance < 35 MΩ were included in the analysis.

### Electrophysiological recordings in acute hippocampal slices

Hippocampal slices with a thickness of 300 μm were prepared from 5-month-old *App*^*NL-G-F*^ mice and littermate wild-type mice. The mice were decapitated under isoflurane anesthesia. For the dissection, a cutting solution composed of the following concentrations (in mM) was used: 229 mannitol, 3 KCl, 23 NaHCO_3_, 1.23 NaHPO_4_, 0.5 CaCl_2_, 3 MgCl_2_, and 10 glucose, with a pH of 7.4. The cutting solution was continuously bubbled with a mixed gas of 95% O_2_ and 5% CO_2_. After the dissection, the slices were kept at room temperature in ACSF solution with the following concentrations (in mM): 124 NaCl, 3 KCl, 2 CaCl_2_, 2 MgCl_2_, 1.23 NaH_2_PO_4_, 26 NaHCO_3_, and 10 glucose, with a pH of 7.4. The ACSF solution was also bubbled with a mixed gas of 95% O_2_ and 5% CO_2_.

Field excitatory postsynaptic potentials (fEPSPs) were recorded in the stratum radiatum of the CA1 region using the MED64 system (Alpha MED Scientific). The hippocampal slices were placed on the MED64 probe, which consisted of an array of 64 planar microelectrodes arranged in an 8 x 8 pattern (P515A, Alpha Med Scientific). The recording probe covered the entire hippocampal CA1 region. During the experiments, the slices were continuously perfused with ACSF at a rate of 1.5 ml/min at room temperature. Monopolar, biphasic constant current pulses with a duration of 0.1 msec were applied to the Schaffer collateral pathway at 30-sec intervals for electrical stimulation. The amplitude of the electrical stimulation was determined based on the input-output curve, and we used 1/3 of the amplitude that induced the maximum fEPSP response.

To induce long-term potentiation (LTP), a stable baseline of fEPSP amplitude was maintained for at least 10 min, followed by high-frequency stimulation consisting of 100 Hz and 100 pulses. The data were analyzed using the data acquisition software Mobius (Alpha-Med Scientific).

### Data analysis and statistics

Statistical analyses were performed in R (version 4.5.1) using RStudio version 2026.07.1.

Behavioral performance was analyzed using generalized linear mixed-effects models (GLMMs) with a binomial distribution and logit link function (lme4 package). Depending on the analysis, genotype or treatment condition, session or another repeated-measure factor (*e.g*., delay duration or within-session trial position), and their interaction, where applicable, were included as fixed effects, with mouse identity included as a random effect to account for repeated measurements within individual animals. Fixed effects were evaluated using Type III Wald chi-square tests (car package). *Post hoc* comparisons were performed using estimated marginal means (emmeans package), with *p* values adjusted for multiple comparisons using the Holm method. Effect sizes in GLMM analyses are reported as odds ratios (ORs). Simulation-based residual diagnostics showed no significant evidence of non-uniformity, over- or underdispersion, or excess outliers in any of the datasets analyzed using GLMMs. Residual uniformity, dispersion, and outliers were assessed using the testUniformity, testDispersion, and testOutliers functions, respectively, in the DHARMa package. For the comparison of correct-response rates during the first and last 20 trials in *App*^*NL-G-F*^ mice shown in Fig 10K, the corresponding GLMM yielded a singular fit. This analysis was therefore performed using a binomial generalized linear model (GLM), with trial period (first versus last 20 trials), session, and mouse identity included as fixed effects.

For continuous outcomes involving repeated measurements, comparisons were performed using linear mixed-effects models (LMMs). For one-group analyses, session or the relevant repeated-measure factor was included as a fixed effect. For two-group analyses, genotype (or treatment), the repeated-measure factor, and their interaction were included as fixed effects. In both models, mouse identity was included as a random intercept to account for repeated measurements within individual mice. LMMs were fitted using the lmer function in the lme4 package with restricted maximum likelihood (REML) estimation. Main effects and interactions were evaluated using Type III F tests with the Kenward–Roger approximation for denominator degrees of freedom (lmerTest package). When appropriate, *post hoc* comparisons were performed using estimated marginal means calculated with the emmeans package, with *p* values adjusted for multiple comparisons using the Holm method. Cohen’s *d* was calculated using the eff_size function in the emmeans package and converted to Hedges’ *g* using the small-sample correction factor. Hedges’ *g* was reported as an effect-size estimate for pairwise comparisons where applicable. Residual normality was assessed using the Shapiro–Wilk test (shapiro.test), and singularity was assessed using isSingular in the lme4 package. All datasets analyzed using LMMs showed no significant deviation from residual normality and no evidence of singular fits. In the revised mixed-effects analyses, no observations were excluded solely on the basis of statistical outlier testing.

For comparisons between two independent groups not involving repeated measurements, Welch’s *t*-test or the Mann–Whitney U test was used, as appropriate. Paired comparisons were performed using the paired *t*-test where appropriate. Differences between cumulative distributions were assessed using the two-sample Kolmogorov–Smirnov test. For the histological analyses shown in S19 Fig, measurements from multiple sections were averaged within each mouse, and the individual mouse was treated as the experimental unit. For S19C Fig, mouse-level averages were compared between genotypes using Welch’s *t*-test. For S19D Fig, inside–outside differences were calculated for each section and averaged within each mouse, and the resulting mouse-level paired measurements were compared using a paired *t*-test.

For the electrophysiological analyses shown in S20 and S21 Fig, all genotype comparisons were performed using heteroscedastic linear mixed-effects models fitted with the lme function in the nlme package. Individual cells (S20 Fig) or slice recordings (S21 Fig) were retained as observational units, with genotype included as a fixed effect and mouse identity as a random intercept to account for the non-independence of multiple recordings obtained from the same animal. Genotype-specific residual variances were allowed using the varIdent variance structure. Genotype effects on mean electrophysiological measures were evaluated by F tests of the fixed genotype effect. Normality of normalized residuals was assessed using the Shapiro–Wilk test.

For the analysis of cell-to-cell variability in the I/E ratio in S20C Fig, a homoscedastic model assuming a common residual variance across genotypes was compared with the heteroscedastic model allowing genotype-specific residual variances. Both models were fitted by maximum likelihood, and model fits were compared using a likelihood-ratio test. Because normalized residuals from models fitted to untransformed I/E ratios deviated significantly from normality, I/E ratios were natural-log transformed before the final analysis.

Statistical significance was defined as *p* < 0.05. Omnibus tests of fixed effects were not adjusted for multiple comparisons; *p* values for *post hoc* pairwise comparisons were adjusted using the Holm method. All tests were two-sided unless otherwise specified. Prespecified comparisons with chance-level performance were one-sided. Mean correct-response rates were calculated for each animal and session for graphical presentation, whereas inferential analyses of operant-task performance were based on trial-level correct/incorrect outcomes in the binomial GLMMs or, for the *App*^*NL-G-F*^ analysis shown in Fig 10K, the binomial GLM described above.

### Figure preparation

All icons and schematic illustrations were created by authors of this article using the Shapes feature in Microsoft PowerPoint. No third-party assets, logos or illustrations were used.

## Results

### Design of the Operant House for automated operant conditioning in individual home cages

Lever-press tasks have been widely used for operant conditioning in rodents. More recently, operant paradigms using visual stimuli presented on liquid crystal display (LCD) touchscreens combined with nose-poke responses have been developed, enabling assessment of higher-order cognitive functions for non-human primate and rodent models [31]. However, in most operant conditioning paradigms, individual animals must be transferred from their home cages to dedicated operant chambers for each session (Fig 1A). This process is labor-intensive for experimenters and can impose additional stress on the animals. To address these issues, we developed an automated operant conditioning system, the Operant House, which allows each mouse to perform operant tasks within its own individual home cage (Fig 1B). The Operant House was designed according to the following basic principles:

1 Task execution in individual home cages

To maintain compatibility with previously reported touchscreen-based operant chambers [32], we used home cages with the same internal dimensions (215 × 145 × 205 mm) and replaced one wall of each home cage with an LCD screen. For task control, each home cage was equipped with its own computer: either a laptop PC (Fig 1C) or a single-board computer (Raspberry Pi 4; Fig 1D).

2 Use of a conventional LCD instead of an LCD touch panel

Commercial LCD touch panels are costly and, in our hands, did not reliably detect mouse nose pokes because their sensing systems are calibrated to detect larger contacts, such as human fingertips. We therefore placed an additional masking wall in front of a conventional LCD monitor with one or more small apertures and detected nose pokes into these apertures using infrared (IR) light as operant responses.

3 Long-term, fully automated task performance with minimal stress in a standard housing environment

As in regular housing cages, wood chips were used as bedding inside the operant chamber. A white light-emitting diode (LED) mounted on the ceiling was turned on during the light phase to maintain circadian rhythm, while operant tasks were conducted during the dark phase, when mice are most active. Mouse behavior and task progression were continuously monitored and recorded with an IR camera. To facilitate learning, water access during the dark phase was restricted to a 10-h period, during which mice could obtain water by performing the operant task correctly. A green LED served as the conditioned stimulus signaling a correct response, whereas brief illumination of the ceiling white LED was used as a mild aversive stimulus after incorrect responses.

Although the system is designed for individually housed mice, it also enables continuous monitoring. Task operation itself does not require continuous user intervention; however, mice were checked daily, including measurement of body weight and visual assessment of their general condition, as described in the Methods. The system can also be accessed and controlled remotely via software such as TeamViewer or Chrome Remote Desktop. Through this remote desktop access, users can monitor live videos from the IR camera mounted on the ceiling of the device or review recorded videos from task sessions to assess task engagement, animal health status, signs of stress, and proper device operation from an office computer. Task outcomes can also be sent automatically by e-mail.

4 Facilitation of dissemination to the research community

The Operant House was built from inexpensive, widely available components. All tasks were controlled by open-source Python programs with a graphical user interface. The software implements a variety of operant paradigms, and users can modify the programs as templates for their own task designs.

### Development of Operant House version 1

As a first prototype based on the above design, we constructed Operant House version 1 using polyvinyl chloride (PVC) plates for the chamber walls (Fig 2A). A low-cost laptop computer controlled the entire system and interfaced via a universal serial bus (USB) connection with a microcontroller board (Arduino Uno, Arduino LLC, Italy), which drove the peripheral devices (Fig 1C). Nose pokes directed toward visual stimuli presented on the LCD monitor and made through the apertures in the masking screen were detected by an IR sensor (AirBar, Neonode, Sweden, or NNAMC3460PCEV, Neonate Inc.; Fig 2B–D).

**Fig 2:**
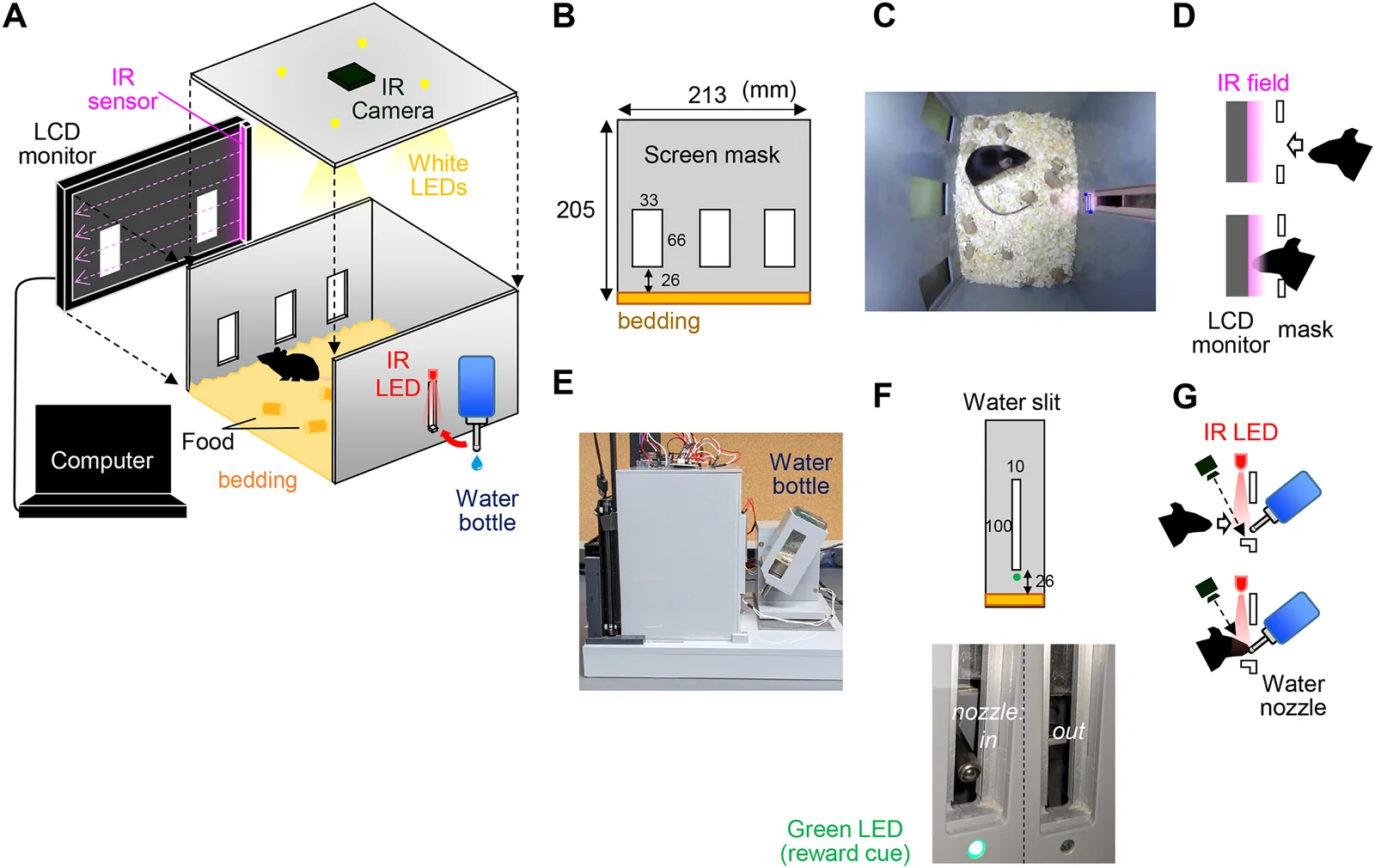
Assembly of the Operant House version 1. The front wall of the Operant House is composed of an LCD monitor (**A**) and a screen mask with open windows (**B**). Food is continuously available on the floor (**C**). An IR sensor generates an infrared field on the monitor surface to detect nose pokes (**D**). When the operant task is successfully completed, a water bottle tilts forward through a gap in the rear wall (**E**), allowing the mouse to drink (**F**). A green LED below the gap lights up as a cue indicating a reward. During the nighttime, an IR LED above the gap illuminates the area, and the drinking behavior of the mouse is recorded by an IR camera mounted on the ceiling (**G**). White LEDs are also installed on the ceiling (**A**); they illuminate the cage during the day and briefly turn on at night as a punishment.

Upon a correct response, a green LED mounted on the wall opposite the LCD was illuminated, and a water-bottle holder behind a slit was rotated into the cage interior by a servo motor, presenting the spout to the mouse (Fig 2E, F). Drinking behavior was also monitored using an IR camera (Fig 2G).

### Development of improved Operant House version 2

In version 1, construction required cutting PVC sheets into multiple sizes and gluing them together. Consequently, scaling up to many Operant House units substantially increased production effort and cost. To overcome this limitation, we developed an improved Operant House version 2, in which the chamber is fabricated by fused deposition modeling (FDM) 3D printing using polylactic acid filament. The use of 3D printing also increases the flexibility in chamber geometry. For example, one wall can be replaced with a panel containing a retractable lever, enabling conventional lever-press tasks (S13A–S13D Fig) and thereby greatly expanding the range of operant paradigms that can be implemented within the Operant House.

In version 1, each Operant House was equipped with a laptop PC for task control, requiring additional space to dissipate the heat generated by multiple laptops. In version 2, we replaced the laptop PCs with single-board computers (Raspberry Pi 4B) equipped with secure digital (SD) memory cards for data storage (Fig 1D). While the original IR sensors could still be used for nose-poke detection (Fig 4C), we also implemented a lower-cost system based on IR LEDs and an IR camera (Fig 3A, B; Fig 4B). In addition to reducing costs, this new detection system has an advantage that its sensitivity can be adjusted by changing the position of the detection zone (Fig 3C).

**Fig 3:**
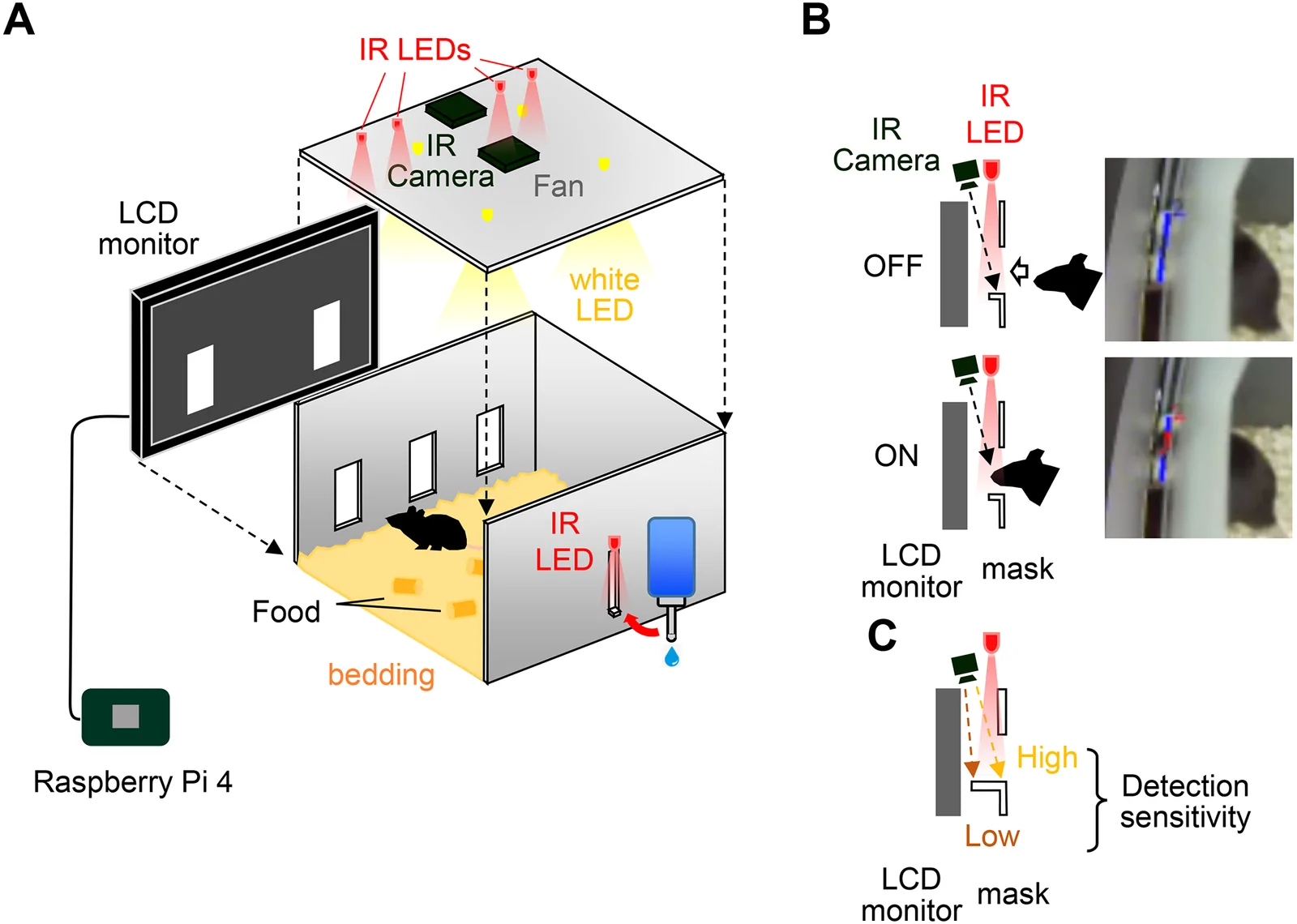
Assembly of Operant House version 2. Similar to version 1, the Operant House consists of an LCD monitor and a screen mask with open windows, with food continuously available on the floor. When the operant task is successfully completed, a water bottle tilts forward through a gap in the rear wall, enabling the mouse to drink (**A**). Unlike version 1, nose-poke actions are detected using image recognition of mouse movements, eliminating the need for an IR sensor (**B**). This is achieved by adding four IR LEDs to the ceiling. The sensitivity of nose-poke detection can be fine-tuned by shifting the detection points in the image captured by the IR camera (**C**). All devices are controlled by a single-board computer (Raspberry Pi 4).

**Fig 4:**
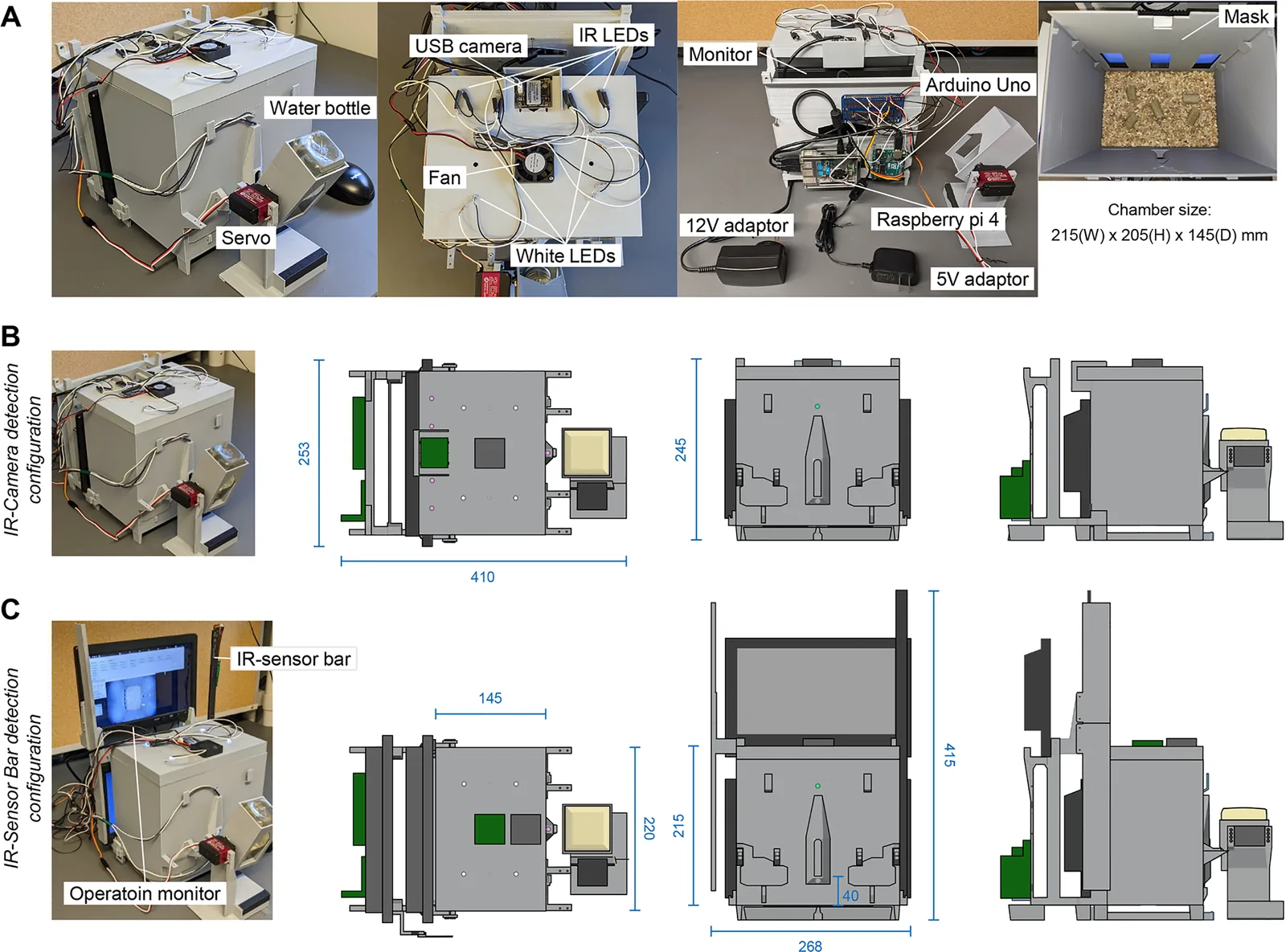
Dimensions of the Operant House Version 2. (**A**) External view of the Operant House. (**B**-**C**) Dimensions of the Operant House configured for IR camera-based nose-poke detection (**B**) and IR sensor bar-based nose-poke detection (**C**).

Taken together, these improvements made Operant House version 2 easier to construct, substantially reduced the cost (to approximately US$380 per unit), and facilitated automated operant testing of multiple individually housed mice in parallel.

### Implementation of Operant House version 2

To implement Operant House version 2, we used parts that are widely available worldwide (S1 Fig) and standard tools commonly found in biological research laboratories (S2 Fig). Chamber components were fabricated using a FDM 3D printer (S3 Fig), assembled (S4 Fig), and then equipped and wired with the Raspberry Pi, LEDs, Arduino Uno, and other electronic components (S5–S11 Fig). Finally, we configured the Raspberry Pi and installed the Operant House software (S12 Fig).

The completed Operant House version 2 had the following specifications (Fig 4):

- Illuminance inside the chamber when the ceiling light was on: 380 lux, measured at the cage floor.
- Camera: 30 frames/s; data size ~35.6 MB/h (recorded at 5 frames/s, 320 × 240 pixels)
- Noise level: fan, +0 dB; servo motor, +10–17 dB (relative to a baseline ambient noise level of 40 dB)
- Touch (nose-poke) detection: sampling frequency, 30 Hz; automated counts matched manual counts by a single observer during the first 30 min of the VD2 session (S1 Video).
- Long-term operation: overall system stable for >1 month; water-tank capacity sufficient for ~2 weeks; bedding replacement required approximately every 3 weeks
- Implemented operant tasks: visual discrimination, spatial discrimination, DNMTP, pattern separation tasks (TCSD and trial-unique nonmatching-to-location [TUNL]), delayed alternation, habit formation tasks, probabilistic reversal learning, Go/No-Go, lever-press tasks, allocentric/egocentric learning paradigms, and 5-CSRTT. Of these, visual discrimination, lever pressing, DNMTP, and TCSD were experimentally evaluated in mice in the present study. TUNL has been evaluated in a separate study [39], whereas the remaining paradigms have been implemented and technically tested but have not yet undergone systematic behavioral validation in animals.
- Water delivery: 25–35 μl per lick.
- Although the 3D-printed surface was less smooth than polyvinyl chloride (PVC) or aluminum, which are commonly used in operant chambers, its surface quality was sufficient for housing mice (S11C–S11E Fig).

### Assessment of lever pressing tasks using Operant House

To assess the efficiency of simple operant conditioning in the Operant House, we trained mice on a two-lever pressing task in which a press on either lever delivered a water reward. On day 1, mice were introduced to the lever–reward contingency while their movements were constrained by placing a wire grid in front of the levers; from day 2 onward, the grid was removed to allow unrestricted access. Lever-press rates increased across training days (S13E Fig). Consistent with this improvement, mice required approximately 9 min and 4 min to obtain the first 30 lever presses on days 2 and 3, respectively (S13F Fig).

For comparison, using a conventional paradigm in which animals are transferred from their home cages to a dedicated operant chamber, mice required 50 min and 28 min on days 2 and 3, respectively, to reach 30 lever presses [4]. Thus, mice acquired lever pressing rapidly under the Operant House training conditions, although direct quantitative comparison with the previous study is limited by differences in experimental conditions and training procedures.

### Assessment of working memory through the DNMTP task using Operant House

To evaluate the performance of the Operant House, we designed a working memory assessment protocol using a DNMTP task with Operant House version 1. In this test, the subject was required to remember the position of one of three illuminated panels during the sample phase and to choose the panel on the opposite side during the choice phase to receive a water reward. The protocol consisted of three blocks (Fig 5A, refer to Methods for detailed description):

**Fig 5:**
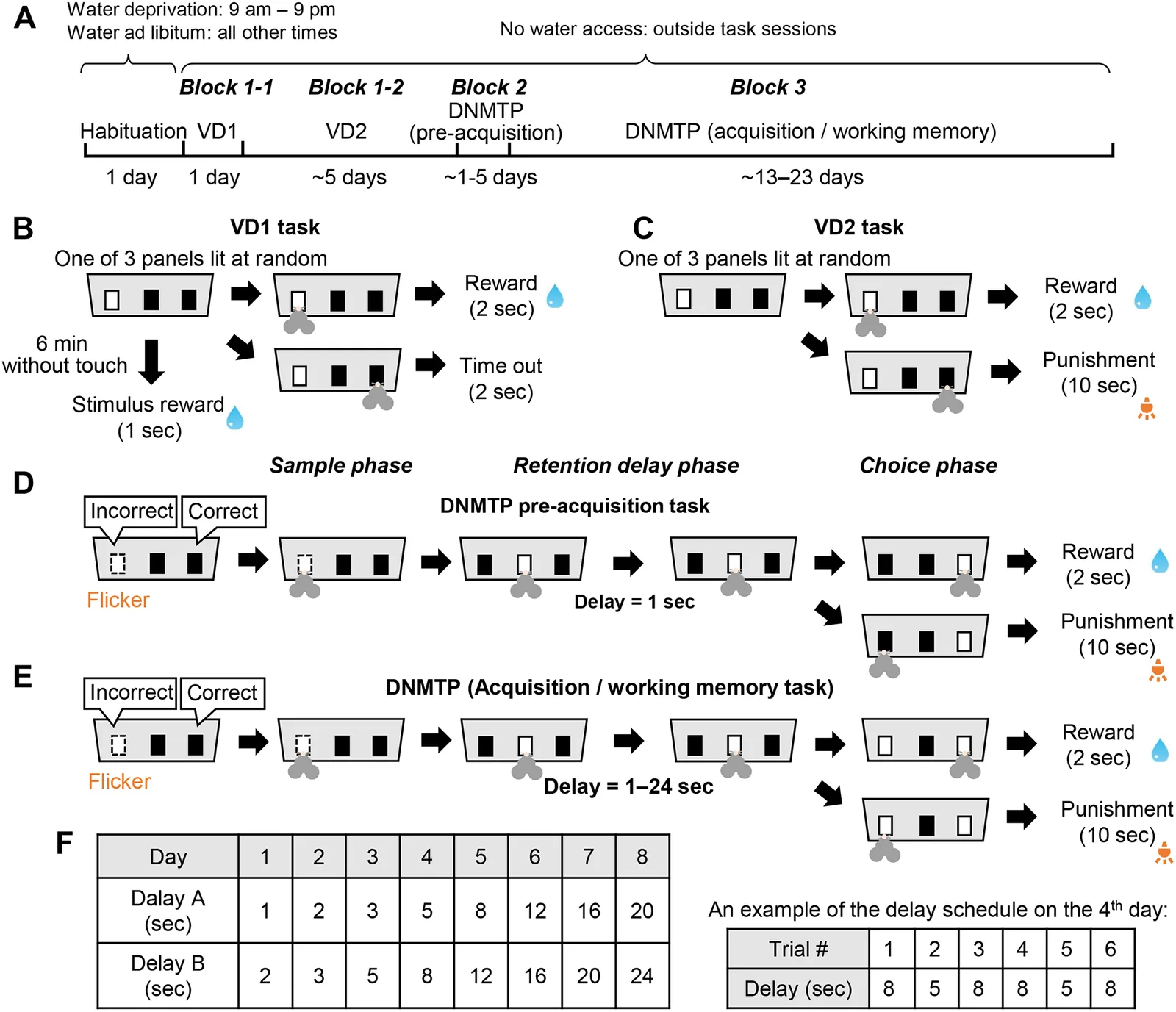
Protocol for the delayed non-matching-to-position (DNMTP) test. (**A**) The DNMTP experiment consisted of three blocks: Block 1 (visual discrimination tasks VD1 and VD2), Block 2 (DNMTP pre-acquisition task), and Block 3 (DNMTP task). The water restriction schedule is indicated above. (**B**) VD1 task. Touching an illuminated panel was rewarded with water, whereas touching an unilluminated panel resulted in a 2-sec timeout. If the subject did not respond to any panel within 6 min, a 1-sec stimulus water reward was delivered. (**C**) VD2 task. Same as VD1, except that incorrect responses were penalized with a 10-sec light stimulus and omission of the stimulus water reward. (**D**) DNMTP pre-acquisition task. Subjects were trained to complete a sequence of four touches to receive water. The first touch was on a blinking panel (left or right), the second and third touches were on the middle panel, and the fourth touch was on the panel opposite to the initial touch. (**E**) DNMTP task. Same as the pre-acquisition task, except that during the choice phase both left and right panels were illuminated. The interval between the second and third touches (on the middle panel) constituted the delay phase, starting at 1 sec until the criterion was met. (**F**) (Left) Delay settings in the DNMTP Task. Two delay schedules (A or B) were applied randomly to each trial, and the delay durations were progressively increased across days. (Right) Representative delay schedule for an animal on day 4 of the working memory phase.

In the first block, mice performed visual discrimination tasks, VD1 and VD2, and learned to touch illuminated panels to receive water rewards (Fig 5B, C). By the end of day 1, the average correct rate for the VD1 task in wild-type mice was significantly higher than the chance level (Fig 6A, GLMM: OR = 1.87, one-tailed *p* < 0.0001). Throughout the VD2 task, the success rate gradually improved, reaching 76.5% on the fourth day (Fig 6B, GLMM, session effect: χ^2^(3) = 21.45, *p* < 0.0001; day 4 vs day 1: OR = 1.96, *p* < 0.0001).

**Fig 6:**
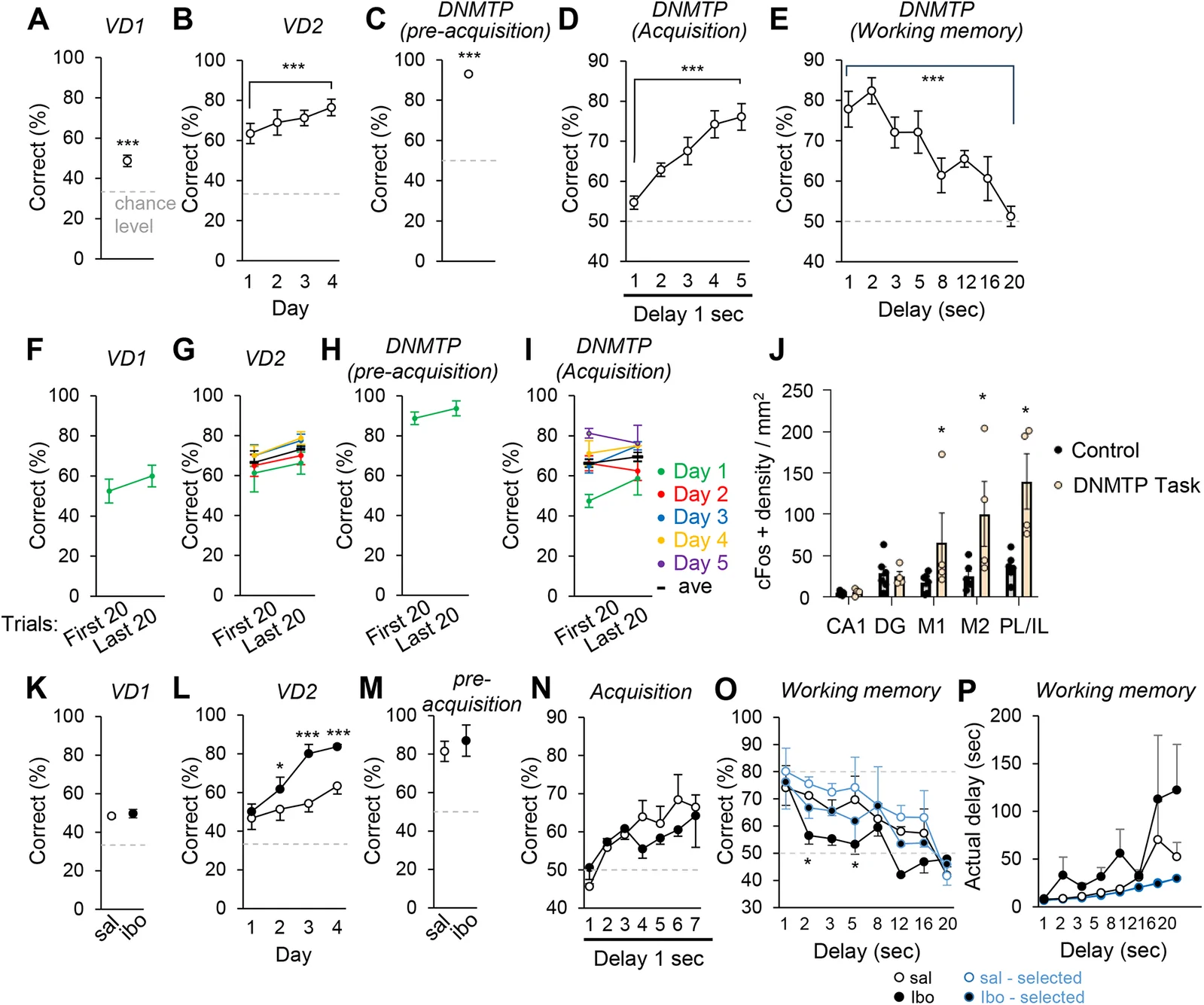
Working memory assessment using the DNMTP test in wild-type mice. (**A-E**) Average correct rates were calculated across all trials per day for the VD1 (**A**), VD2 (**B**), DNMTP pre-acquisition (**C**), DNMTP acquisition with a 1-sec delay (**D**), and DNMTP working memory tasks (**E**). The dotted lines indicate chance-level performance. Note the decline in correct rates as delay intervals increased in the working memory task (**E**). (**F-I**) Comparison of correct rates between the first and last 20 trials of each task on the same day. No significant differences were observed for VD1 (**F**, GLMM: χ^2^ (1) = 0.23, OR = 1.16, *p* = 0.6340), VD2 (**G**, GLMM: χ^2^(1) = 3.22, OR = 1.38, *p* = 0.0726), DNMTP pre-acquisition (**H**, GLMM: χ^2^ (1) = 1.99, OR = 2.41, *p* = 0.1584), or DNMTP acquisition tasks (**I**, GLMM: χ^2^(1) = 0.64, OR = 1.13, *p* = 0.4229). (**J**) c-Fos expression. c-Fos-positive cell densities were significantly increased in M1, M2, and PL/IL in mice performing the DNMTP working-memory task compared with home-cage controls (LMM: task × region, *F*_4,32_ = 5.68, *p* = 0.0014, *post hoc*: M1 (*p* = 0.0496, Hedges’ *g* = 1.57), M2 (*p* = 0.0031, Hedges’ *g* = 2.47), PL/IL (*p* = 0.0001, Hedges’ *g* = 3.36)). The density of c-Fos-positive neurons was measured in brains fixed 60–90 min after initiating the task (with delays of 8–12 sec). (**K-O**) Average correct rates during VD1 (**K**), VD2 (**L**), DNMTP pre-acquisition (**M**), and DNMTP acquisition with a 1-sec delay (**N**). (**O**) Results of the DNMTP working memory task. Average correct rates in mice injected with saline (open circles) or ibotenic acid (filled circles). Blue-outlined symbols indicate trials that met the actual delay-time criteria (see main text). (**P**) Average actual delay duration during the DNMTP task. *n* = 4 mice (**A-I**); *n* = 6 and 4 mice for control and DNMTP task group, respectively (**J**); *n* = 3 mice (**K-P**) per group. GLMM (vs. chance; one-tailed): ***p < 0.0001 (**A**, **C**). GLMM with Holm-adjusted *post hoc* tests: ***p < 0.0001; *p < 0.05 (**B, D, E, L, O**). LMM with Holm-adjusted *post hoc* tests: ***p < 0.0001; *p < 0.05 (**J**). All graphs show mean ± SEM.

In the second block, mice performed the DNMTP pre-acquisition task. Mice received a water reward after touching the correct panel during the choice phase. The correct panel was on the opposite side of the panel presented during the sample phase. The choice phase followed the sample phase after a fixed 1-sec delay phase. In this DNMTP pre-acquisition task, the correct panel was explicitly illuminated during the choice phase to help the mice recognize the rules of the DNMTP task (Fig 5D). Performance was significantly above chance level (Fig 6C; GLMM: OR = 13.33, one-tailed *p* < 0.0001).

In the third block, mice first performed the DNMTP acquisition task with a fixed 1-s delay. The success rate increased daily in wild-type mice, reaching 76.5% within 5 days (Fig 6D; GLMM; session effect: χ^2^(4) = 65.14, *p* < 0.0001; day 5 vs day 1: OR = 2.60, *p* < 0.0001).

In the third block, working memory was assessed by increasing the duration of the delay phase (Fig 5E) according to a set schedule (Fig 5F). The correct response rate decreased with increasing delay duration (Fig 6E, GLMM; delay effect: χ^2^(7) = 61.75, *p* < 0.0001; delay 20 s vs delay 1 s: OR = 0.32, *p* < 0.0001).

Motivation is a critical factor influencing behavioral outcomes in tasks. Because the level of thirst influences motivation in our tasks, the correct rate and the frequency of trials may decline with reduced motivation after repeating trials and obtaining rewards. However, we did not observe a decrease in correct rates as trials were repeated each day. The correct rates in the first and last 20 trials were similar in VD1, VD2, DNMTP pre-acquisition, and DNMTP acquisition tasks (Fig 6F-I). This trend was reflected in the average correct rate for 10-trial bins each day (S14A–S14E Fig). Most mice completed trials after achieving 80 correct responses (S14F–S14J Fig). The average duration of the entire trials per day was less than 3 hours except for the DNMTP task, which included delay phase between sample and choice phase (S14K–S14O Fig). The duration of each trial in the VD2 and DNMTP acquisition tasks was similar across repeated trials in a day (LMM: VD2, *F*_3, 9_ = 1.73, *p* = 0.23, Hedges’ *g* = 1.39 for day 4 vs day 1; DNMTP acquisition, *F*_4, 12_ = 1.22, *p* = 0.35, Hedges’ *g* = 1.16 for day 5 vs day 1; S6F, S6I Fig). These findings provided no evidence of a systematic decline in task engagement across repeated trials within a session. In six wild-type mice from independent Operant House version 2 experiments using the same programmed DNMTP water-access regimen, body weight showed no significant group-level change between measurements obtained before and after testing (22.47 ± 0.52 g vs. 21.89 ± 1.11 g, respectively; paired t-test, t(5) = −0.65, *p* = 0.545).

To identify the brain regions responsible for the DNMTP task, we performed immunostaining for c-Fos, a marker of neuronal activation. We observed significantly increased c-Fos immunoreactivity in the higher motor area (M1 and M2) and the prelimbic and infralimbic cortex (PL/IL) of mice that underwent the DNMTP task compared to homecage control mice (LMM: task × region, *F*_4,32_ = 5.68, *p* = 0.0014, *post hoc*: M1 (*p* = 0.0496, Hedges’ *g* = 1.57), M2 (*p* = 0.0031, Hedges’ *g* = 2.47), PL/IL (*p* = 0.0001, Hedges’ *g* = 3.36); Fig 6J).

To further examine the contribution of these c-Fos-positive regions to DNMTP performance, we induced bilateral lesions of the PL/IL and M2 by injection of ibotenic acid (IBO). Across the training blocks, IBO- and saline-injected mice showed comparable correct response rates during repeated trials (Fig 6K–N; S15A–S15D Fig). In addition, both groups completed the task similarly, reaching 80 correct responses (S15E–S15H Fig) within comparable time periods (S15I–S15P Fig), indicating that motivation was not detectably affected by IBO treatment. During the working memory phase, IBO-treated mice showed significantly lower performance than saline-treated mice (Fig 6O; GLMM: χ^2^(1) = 5.98, OR = 0.70; *p* = 0.0145).

Behavioral observation revealed that mice occasionally engaged in eating or sleeping during the delay phase, resulting in prolongation of the actual delay duration, defined as the interval between touches of the sample and choice panels (Fig 6P). To assess whether variability in actual delay duration contributed to the observed group difference, we performed an additional sensitivity analysis restricted to trials in which the actual delay duration was within 1.3 times the programmed delay plus 10 s. This criterion retained 1,231 of 1,783 trials (69.0%) in saline-treated mice and 1,309 of 2,013 trials (65.0%) in IBO-treated mice; consequently, 552 (31.0%) and 704 (35.0%) trials, respectively, were excluded. The mean actual delay duration did not differ between IBO- and saline-treated mice under this criterion (Fig 6P, blue outlines). The lower correct-response rate in IBO-treated mice observed in the primary all-trial analysis was also detected in this delay-restricted sensitivity analysis (GLMM: χ^2^(1) = 4.07, OR = 0.71; *p* = 0.0435; Fig. 6O, blue outlines).

The DNMTP task can be conducted using Operant House Version 2 with a slight modification in the protocol (S16A–S16F Fig), as described in the Methods section. Together, these results suggest that the DNMTP task effectively assesses working memory in wild-type mice, which is consistent with a contribution of PL/IL and/or M2.

### The DNMTP task detected early working memory deficit in female *App*^*NL-G-F*^ mice

In humans, a working memory deficit is suggested to be one of the earliest signs of pre-symptomatic AD [20–22]. However, the presence of early working memory deficits has remained inconsistent across studies using *App*^*NL–G–F*^ knock-in mice, which are considered to more faithfully recapitulate human AD pathology [26–29]. Thus, to test the effectiveness of the Operant House, we assessed working memory in *App*^*NL-G-F*^ mice using the DNMTP test.

*App*^*NL-G-F*^ mice showed a higher correct-response rate in VD1, whereas correct-response rate did not differ significantly between genotypes in VD2 or the DNMTP pre-acquisition task (GLMM; VD1, χ^2^(1) = 5.39, OR = 1.56; *p* = 0.020; VD2, χ^2^(1) = 1.63, OR = 1.35; *p* = 0.202; pre-acquisition, χ^2^(1) = 0.04, OR = 1.09; *p* = 0.835) (Fig 7A-C). However, the performance in DNMTP acquisition task differed significantly between wild-type and female *App*^*NL-G-F*^ mice. While wild-type mice reached the criteria of the DNMTP acquisition task (success rate > 80% for 2 successive days with 1-sec fixed delay) in 12 ± 1 days (mean ± sem), female *App*^*NL-G-F*^ homozygous mice took 22 ± 3 days, significantly longer than the wild-type (Mann-Whitney U test: *p =* 0.0097) (Fig 7D, E).

**Fig 7:**
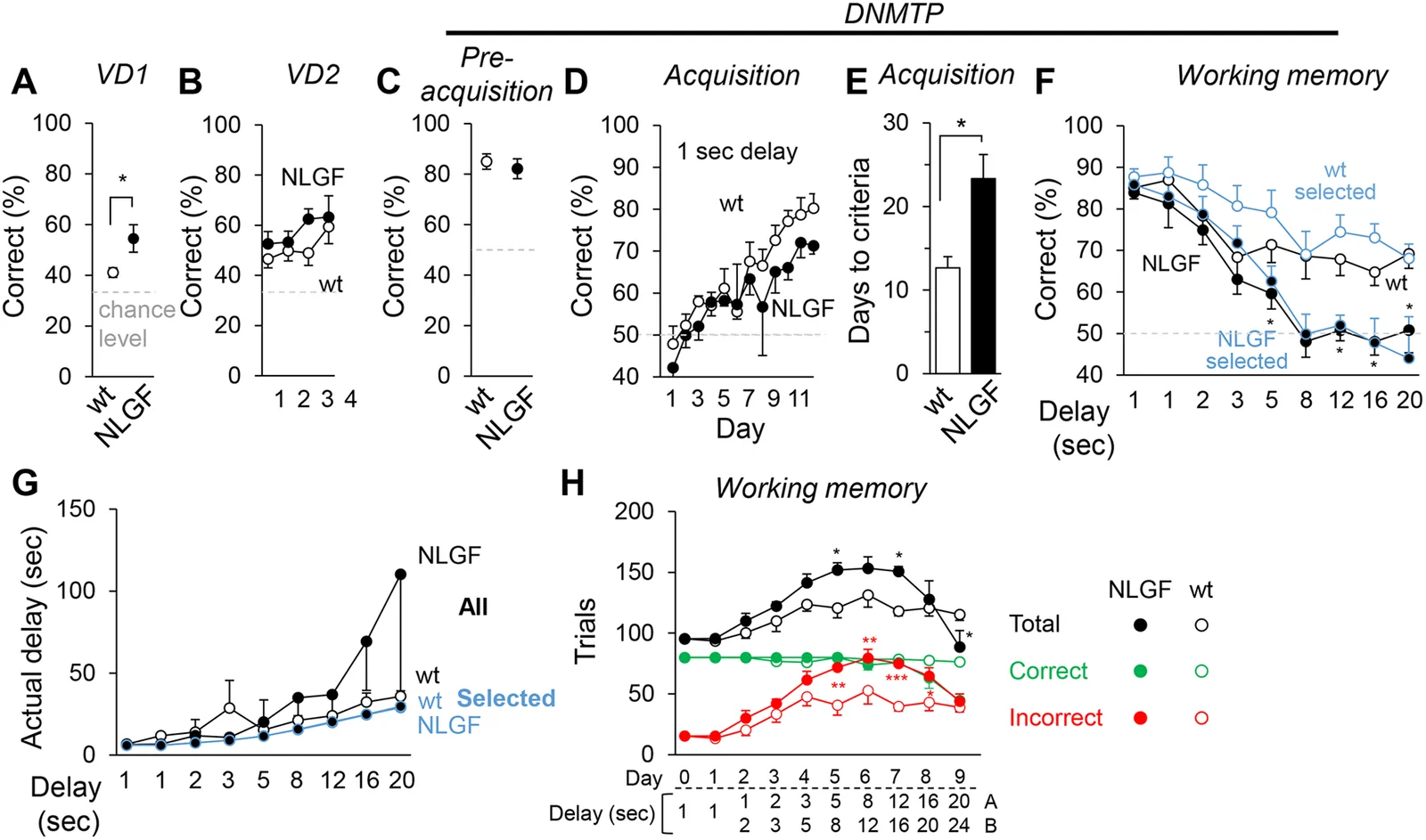
Female *App*^*NL−G−F*^ mice exhibit working memory deficits in the DNMTP test at 5 months of age. (**A-C**) Average correct rates for VD1 (**A**), VD2 (**B**), and DNMTP pre-acquisition tasks (**C**) in wild-type (open circles) and *App*^*NL−G−F*^ (filled circles) mice. Dotted lines indicate chance-level performance. (**D-E**) Daily improvements in correct rates during the DNMTP acquisition task with a 1-sec delay were slower in *App*^*NL−G−F*^ mice (**D**). These mice required more days to reach the performance criterion (80% correct for two consecutive days) (**E**). (**F**) In the DNMTP working memory task, correct rates in *App*^*NL−G−F*^ mice dropped sharply when the delay was 2 sec or longer. Blue-outlined plots represent trials meeting the criteria for actual delay times (refer to the main text). (**G**) No difference in actual delay times was observed between wild-type and *App*^*NL−G−F*^ mice when comparing trials that met the criteria for actual delay time (Blue-outlined plots). (**H**) *App*^*NL−G−F*^ mice performed more total trials in the DNMTP working memory task. This increase was observed in incorrect trials but not in correct trials. GLMM: *p < 0.05 (**A**). Mann-Whitney U test: *p < 0.05 (**E**). GLMM and LMM with Holm-adjusted *post hoc* tests: ***p < 0.0001; ** *p* < 0.005; *p < 0.05 (**F, H**). *n* = 6 mice per genotype.

After reaching the criteria above, the mice performed DNMTP task with a longer delay time to assess working memory. The correct rate in female *App*^*NL-G-F*^ mice showed a significant reduction compared to wild-types when the delay time was longer than 2 sec (GLMM; χ^2^(1) = 6.72, OR = 0.68; *p* = 0.0095) (Fig 7F). Consequently, the number of total and incorrect responses per day was significantly higher in *App*^*NL-G-F*^ mice (LMM; total: genotype × session, *F*_6, 60_ = 4.24, *p =* 0.0013, Hedges’ *g*: day 5, 1.90; day 7, 1.99; day 9, 1.63; incorrect; genotype: *F*_1, 10_ = 10.95, *p =* 0.0079, Hedges’ *g* = 1.52) (Fig 7H).

To assess the impact of motivation levels, we examined the temporal variation of correct responses over trials within a day. Neither wild-type nor *App*^*NL-G-F*^ mice showed any difference in the correct rate with repeated trials (S17A–S17D Fig). In addition, no difference was observed in the total number of trials or the average time spent per trial (S17E–S17P Fig). These results indicate impaired DNMTP performance in female *App*^*NL-G-F*^ mice, without evidence of a systematic reduction in task engagement or basal activity.

Similar to ibotenic acid-treated animals, *App*^*NL-G-F*^ mice tended to show prolonged actual delay durations, which could potentially confound the interpretation of working memory performance (Fig 7G). The primary analysis therefore included all trials, and an additional sensitivity analysis was performed using only trials that met the predefined actual-delay criterion (programmed delay × 1.3 + 10 s). This criterion retained 2,756 of 3,403 trials (81.0%) in wild-type mice and 2,909 of 3,700 trials (78.6%) in *App*^*NL-G-F*^ mice; 647 (19.0%) and 791 (21.4%) trials, respectively, were excluded. Thus, the proportions of retained trials were similar between genotypes. Among the retained trials, mean actual delay durations were comparable between genotypes (Fig 7G, blue outlines). The lower correct-response rate in *App*^*NL-G-F*^ mice observed in the primary all-trial analysis was also detected in this delay-restricted sensitivity analysis (GLMM: χ^2^(1) = 7.71, OR = 0.61, *P* = 0.0055; Fig 7F, blue outlines).

Interestingly, male *App*^*NL-G-F*^ mice did not exhibit any differences in the VD1/2 or DNMTP tasks (S18 Fig) (GLMM: VD1, χ^2^(1) = 2.01, OR = 1.22; *p* = 0.139; VD2, χ^2^(1) = 0.17, OR = 1.09; *p* = 0.680; DNMTP pre-acquisition, χ^2^(1) = 1.07, OR = 1.76, *p* = 0.305; DNMTP working memory, all trials, χ^2^(1) = 0.16, OR = 0.91; *p* = 0.687; selected trials, χ^2^(1) = 0.100, OR = 0.93; *p* = 0.7514; Mann-Whitney U test: Days to criteria in the DNMTP acquisition, *p* = 0.313). Taken together, these results indicate that sex-dependent working memory deficit in female *App*^*NL-G-F*^ mice at early stages [27] could be detected by the DNMTP task in the Operant House.

### Working-memory-related performance assessed with the TCSD task using the Operant House

To assess working memory-related performance by an independent method and to detect any changes in male *App*^*NL-G-F*^ mice, we established a TCSD test, in which mice were required to memorize the correct panel from two options by recalling the position from the previous trial (Fig 8, refer to Methods for detailed description). The correct position was switched to the opposite side after the mice achieved more than 7 correct responses in the most recent 8 trials. To identify the brain regions involved in the TCSD task, we performed prefrontal cortex lesions using ibotenic acid injections. In block1, VD1 and VD2 tasks, in which mice needed to touch a single lit panel, no significant differences were observed between groups (Mann-Whitney U test: VD1, *p =* 0.853; VD2, *p =* 0.826) (Fig 9A, B). In block 2, the TCSD test revealed lower correct rates in the lesion group over 3 days (Fig 9C; GLMM: χ^2^(1) = 12.40, OR = 0.62; *p* = 0.0004). The lesion group also exhibited a significant reduction in the number of reversals (Fig 9D; LMM: *F*_1,5_ = 16.15, *p* = 0.0101, Hedges’ *g* = −1.70), with an increased average trial number per reversal (Fig 9E; LMM: *F*_1,5_ = 23.36, *p* = 0.00475, Hedges’ *g* = 2.68) and the number of the incorrect trials (Fig 9F; LMM: *F*_1,5_ = 10.36, *p* = 0.0235, Hedges’ *g* = 2.18). These results suggest that prefrontal-lesioned animals exhibited impaired TCSD performance.

**Fig 8:**
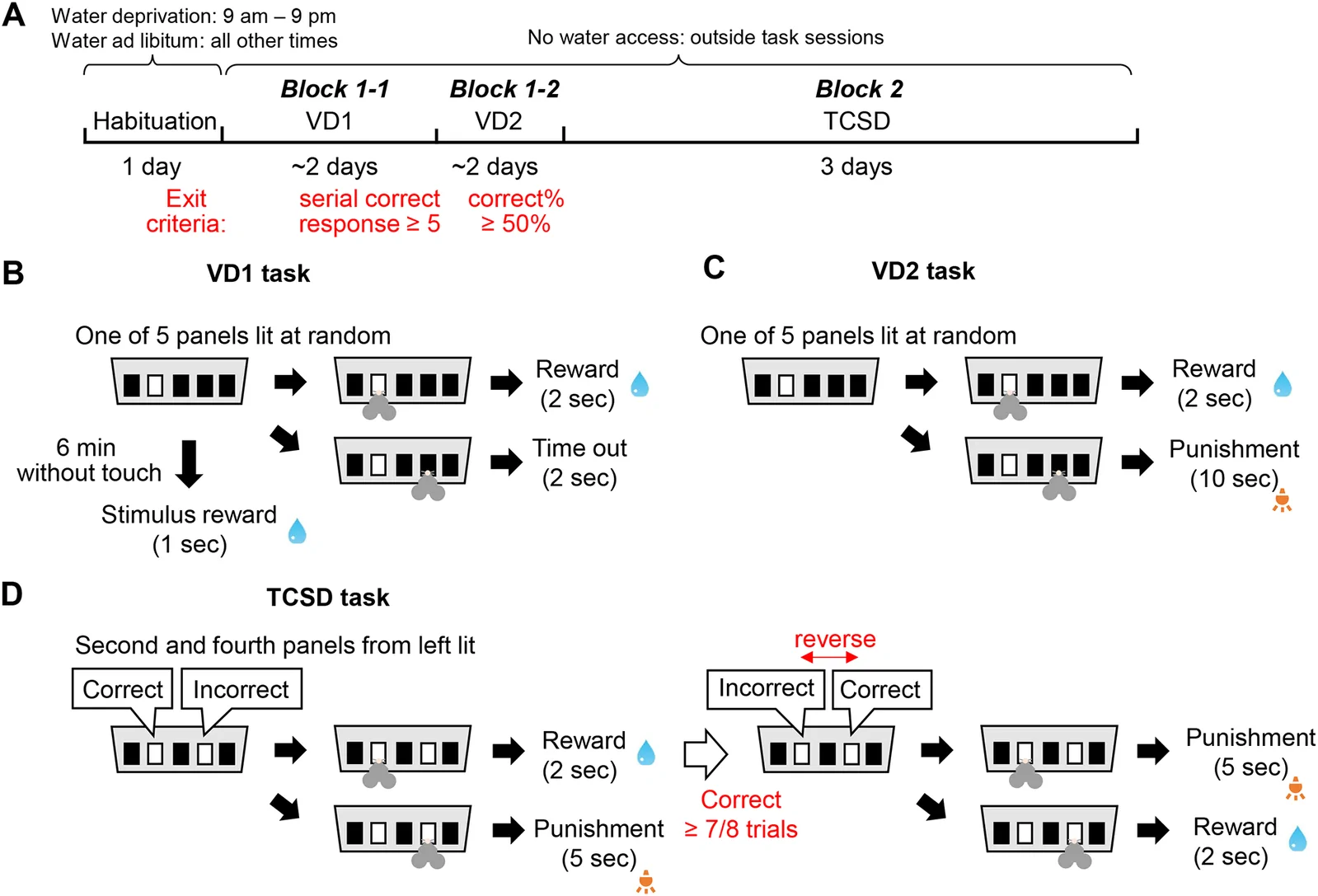
Protocol for two-choice spatial discrimination (TCSD) tests. (**A**) The TCSD test consisted of two blocks: Block 1 included Visual Discrimination 1 (VD1) and VD2 tasks, and Block 2 was the TCSD task. The water restriction schedule is indicated above. (**B**) VD1 Task. Touching the illuminated panel was rewarded with water, while touching unilluminated panels resulted in a 2-sec timeout. If the subject failed to respond within 6 min, a 1-sec water reward was provided as a stimulus. (**C**) VD2 Task. This task was similar to VD1, with the omission of stimulus rewards and the addition of a punishment for incorrect responses consisting of a 10-sec light stimulus. (**D**) TCSD Task. At the start of the trials, the left or right panel was randomly designated as the correct choice. Selecting the correct panel resulted in a 2-sec reward, while choosing the incorrect panel triggered a 5-sec punishment. The correct panel switched after the subject achieved 7 correct responses out of the last 8 trials.

**Fig 9:**
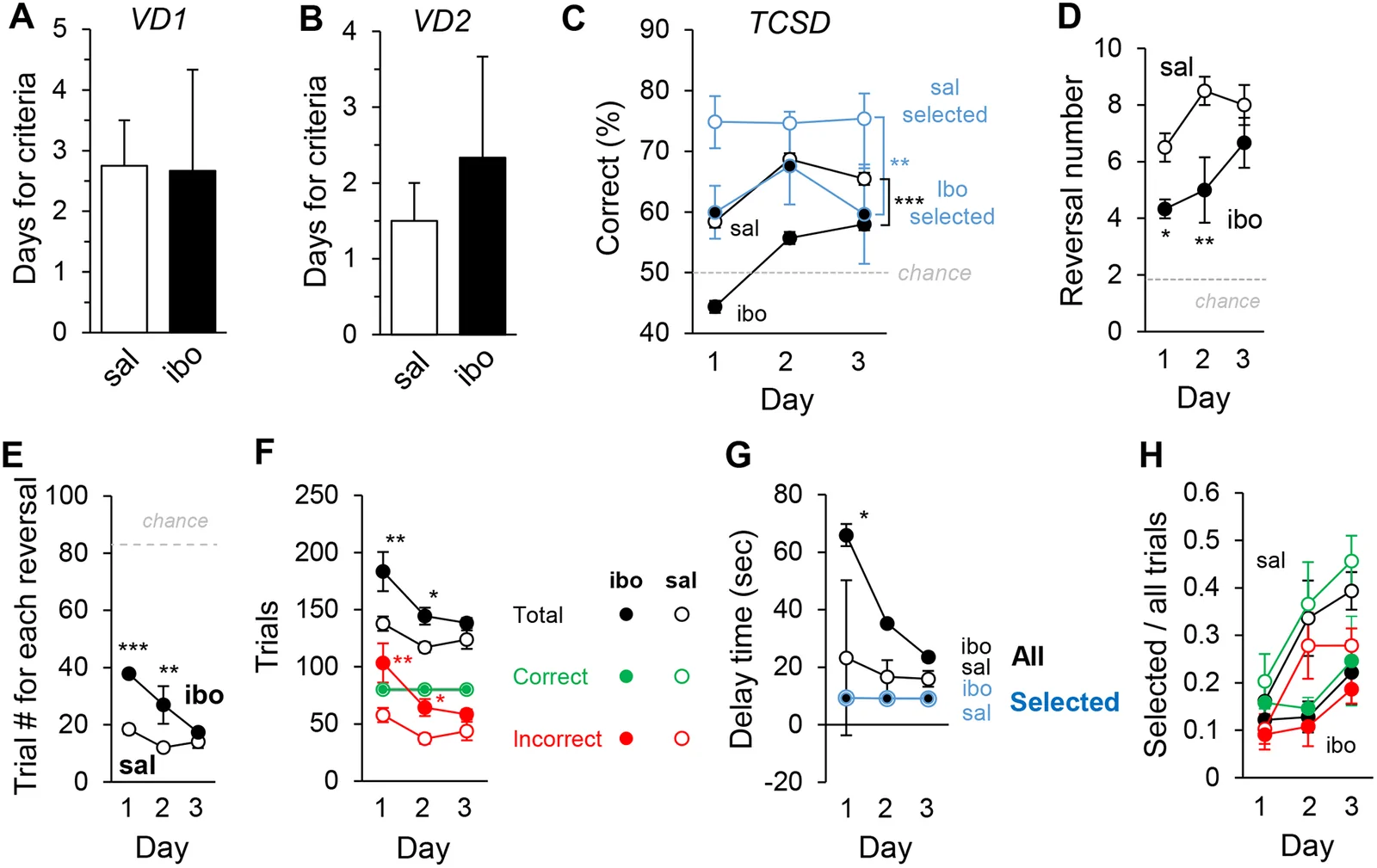
Prefrontal cortex-lesioned wild-type mice exhibit impaired performance in the TCSD task. (**A, B**) The number of days required to reach the performance criterion in VD1 (**A**) and VD2 (**B**) did not differ between saline- and ibotenic acid-treated mice. (**C**) TCSD task performances. The lesioned animals showed lower correct rates than control animals. Blue-outlined plots indicate trials that met the 10 sec criterion for actual delay times. (**D**) Reversals. The number of reversals per day was reduced in the lesion group. (**E**) Trial efficiency. Ibotenic acid-treated mice required more trials per reversal on average than control mice. (**F**) Trial outcomes. The lesion group showed a higher number of incorrect and total trials. (**G**) Touch intervals. Ibotenic acid-treated mice showed an increased mean time between touches. The delay times of trials that met the 10 sec criterion for actual delay times were nearly identical between groups. (**H**) Ratio of selected to total trials. Mann–Whitney U test (**A**, **B**); GLMM (**C**); LMM (**D**–**G**). Holm adjustment was applied to *post hoc* comparisons where applicable. ***p < 0.001; **p < 0.01; *p < 0.05. *n* = 4 (saline) and *n* = 3 (ibotenic acid).

In the TCSD task, although the inter-trial interval was set to 5 sec, mice could spend any amount of time between trials. Indeed, the delay time from touching a panel in one trial to touching a panel in the next was significantly longer in the lesion group (Fig 9G; LMM: *F*_1,5_ = 7.95, *p* = 0.0371, Hedges’ *g* = 1.22). To assess the potential influence of this longer delay duration, we performed an additional sensitivity analysis restricted to trials with actual delay times of 10 sec or less. This criterion retained 445 of 1,514 trials (29.4%) in saline-treated mice and 210 of 1,398 trials (15.0%) in IBO-treated mice; consequently, 1,069 (70.6%) and 1,188 (85.0%) trials, respectively, were excluded. The delay times in these selected trials were comparable between the groups (Fig 9G, blue-outlined circles). Because a substantial proportion of trials was excluded, particularly in the IBO group, this delay-restricted analysis was interpreted as a sensitivity analysis and considered together with the primary analysis based on all trials. Nevertheless, the correct rate remained significantly lower in the lesioned animals for these trials (GLMM: χ^2^(1) = 7.20, OR = 0.61; *p* = 0.0073) (Fig 9C, blue-outlined circles). These findings support a contribution of the prefrontal cortex to TCSD performance.

### The TCSD test detected impaired task performance in male *App*^*NL-G-F*^ mice

Next, male *App*^*NL-G-F*^ mice and their wild-type littermates at 5 months of age were subjected to the TCSD task. There was no significant difference between genotypes in the number of days required to reach the learning criterion in the VD1 or VD2 tasks (Mann-Whitney U test: VD1, *p =* 0.454; VD2, *p =* 0.0689) (Fig 10A, B). In block 2, the male *App*^*NL-G-F*^ mice showed robust impairment in the TCSD task across three days (Fig 10C; GLMM: χ^2^(1) = 12.71, OR = 0.76; *p* = 0.0004) and in each 50-trial bin (Fig 10D; GLMM: χ^2^(1) = 17.98, OR = 0.74; *p* < 0.0001). *App*^*NL-G-F*^ mice also completed fewer reversals (Fig 10E; LMM: *F*_1,12_ = 19.06, *p* = 0.0009, Hedges’ *g* = −2.33) and required more trials to meet the reversal criterion (Fig 10F; LMM: *F*_1,12_ = 14.44, *p* = 0.0025, Hedges’ *g* = 1.46), whereas the total numbers of trials were unaffected (Fig 10G; LMM: *F*_1,12_ = 1.35, *p* = 0.27, Hedges’ *g* = −0.87). These results indicate impaired TCSD performance, consistent with deficits in working-memory-related processes and/or behavioral flexibility and response selection.

**Fig 10:**
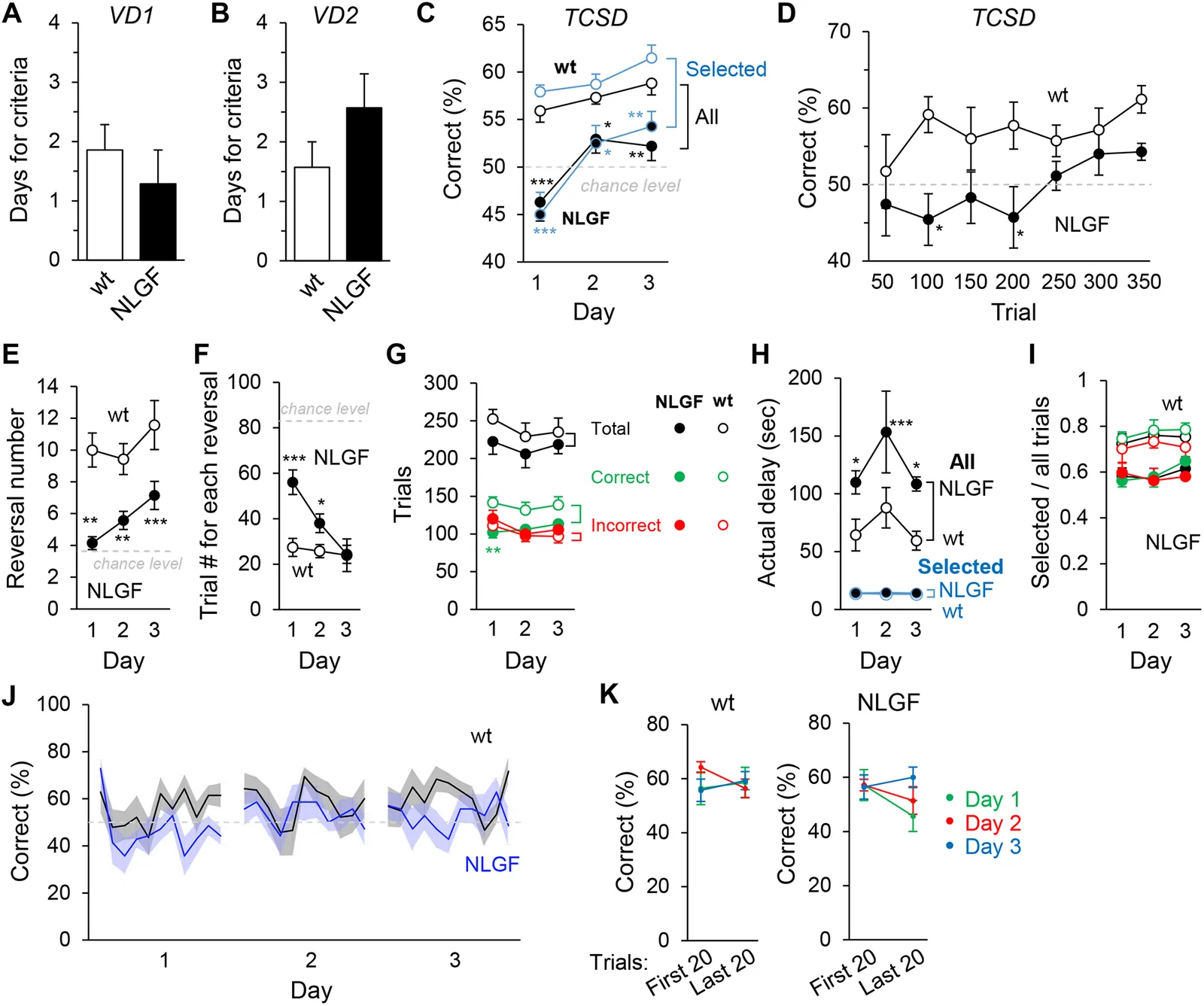
Male *App*^*NL−G−F*^ mice exhibit impaired performance in the TCSD task at 5 months of age. (**A, B**) The number of days required to reach the performance criterion for the VD1 (**A**) and VD2 (**B**) tasks was comparable between male *App*^*NL−G−F*^ and wild-type (wt) mice. (**C**) TCSD task performance. Male *App*^*NL−G−F*^ mice exhibited lower correct-response rates than wild-type mice. Blue-outlined plots represent selected trials that met the criterion for actual delay duration. (**D**) Performance over time. Correct-response rates for each 50-trial bin during the first 350 trials revealed poorer performance in *App*^*NL−G−F*^ mice than in wild-type mice. (**E**) Reversals. The number of reversals per day was reduced in *App*^*NL−G−F*^ mice. (**F**) Trial efficiency. *App*^*NL−G−F*^ mice required more trials per reversal on average than wild-type mice. (**G**) Trial outcomes. *App*^*NL−G−F*^ mice exhibited a lower number of correct trials, whereas the number of incorrect trials was comparable between genotypes. (**H**) Touch intervals. *App*^*NL−G−F*^ mice showed longer average intervals between touches, whereas the actual delay durations were comparable between genotypes when the analysis was restricted to selected trials meeting the predefined delay-time criterion. (**I**) Proportion of trials meeting the predefined actual-delay criterion. More than half of the trials met this criterion in both genotypes. (**J**) Correct-response rates for successive 10-trial bins across daily sessions. The first 11 bins of each day are shown; no obvious systematic decline in correct-response rate was observed over the course of each session. (**K**) Correct-response rates during the first and last 20 trials of each session. No significant difference was detected between the first and last 20 trials in either genotype. Mann–Whitney U test (**A, B**); GLMM (**C, D**); LMM (**E–H**); GLMM for wild-type mice and binomial GLM for *App*^*NL−G−F*^ mice (**K**). No inferential statistical test was performed for (**I**) or (**J**). Holm adjustment was applied to *post hoc* comparisons where applicable. ***p < 0.0001; **p < 0.01; *p < 0.05. *n* = 7 per genotype.

Consistent with results from the DNMTP task, the *App*^*NL-G-F*^ mice exhibited significantly prolonged delay times during the TCSD test (Fig 10H; LMM, genotype effect: *F*_1,12_ = 8.35, *p* = 0.0136, Hedges’ *g* = 1.30). To assess the potential influence of this longer delay duration, we performed an additional sensitivity analysis restricted to trials with actual delay times of 20 s or less. This criterion retained 3,728 of 5,019 trials (74.3%) in wild-type mice and 2,696 of 4,531 trials (59.5%) in *App*^*NL-G-F*^ mice; consequently, 1,291 (25.7%) and 1,835 (40.5%) trials, respectively, were excluded. Thus, although more than half of the trials were retained in both genotypes, a larger proportion of trials was excluded in *App*^*NL-G-F*^ mice. The actual delay times of these selected trials were comparable between genotypes (Fig 10H, blue-outlined circles). Because trial retention differed between genotypes, this delay-restricted analysis was interpreted as a sensitivity analysis and considered together with the primary analysis based on all trials. Nevertheless, correct-response rates remained significantly lower in App^NL-G-F^ mice (Fig 10C, blue-outlined circles; GLMM: χ^2^(1) = 9.47, OR = 0.71, *p* = 0.0021).

Correct-response rates across successive 10-trial bins showed no obvious systematic decline over the course of each daily session in either genotype (Fig 10J). To formally test this, we compared performance during the first and last 20 trials of each session. No significant difference was detected in wild-type mice (GLMM: χ^2^(1) = 0.05, OR = 0.97, *p* = 0.8203) or *App*^*NL-G-F*^ mice (binomial GLM: χ^2^(1) = 1.70, OR = 0.83, *p* = 0.1920; Fig 10K), providing no evidence of a systematic decline in task performance over the course of the daily sessions. Together, these results demonstrate that the Operant House can detect early cognitive deficits in *App*^*NL-G-F*^ mice as early as 5 months of age using two complementary paradigms, DNMTP and TCSD.

### Abnormal synaptic changes in the prefrontal cortex of 5-month-old *App*^*NL-G-F*^ mice

Finally, to determine whether 5-month-old *App*^*NL-G-F*^ mice already exhibit pathology in the prefrontal cortex, which is critically involved in working memory, we examined structural and functional synaptic changes in this region. Immunohistochemistry revealed abundant amyloid-β (Aβ) plaques in the prefrontal cortex of 5-month-old *App*^*NL-G-F*^ (S19A Fig). To assess synaptic alterations, we quantified the excitatory presynaptic marker vesicular glutamate transporter 1 (VGluT1) and the inhibitory presynaptic marker vesicular GABA transporter (VGAT) in the prefrontal cortex. In regions outside Aβ plaques, neither VGluT1 nor VGAT immunoreactivity differed significantly between genotypes, although VGAT showed a tendency toward lower intensity in *App*^*NL-G-F*^ mice (S19B, S19C Fig). Notably, within individual Aβ plaques, both VGluT1 and VGAT immunoreactivities were significantly lower than in the surrounding neuropil (S19D Fig). These findings indicate localized reductions in both excitatory and inhibitory presynaptic markers around Aβ plaques.

To test whether these changes in synaptic markers are accompanied by functional alterations, we next performed whole-cell patch-clamp recordings from pyramidal neurons in acute medial prefrontal cortex (mPFC) slices from 5-month-old *App*^*NL-G-F*^ and wild-type mice. Passive membrane properties of pyramidal neurons did not differ between genotypes (S20A Fig), and the mean frequency and amplitude of miniature excitatory and inhibitory postsynaptic currents (mEPSCs and mIPSCs) were also comparable (S20B Fig). Similarly, the mean amplitudes of excitatory and inhibitory currents (EPSCs and IPSCs) evoked by local stimulation near the recorded cells were not significantly different between groups (S20C Fig). However, cell-to-cell variability in the I/E ratio was significantly greater in *App*^*NL-G-F*^ than in wild-type pyramidal neurons after accounting for the non-independence of multiple cells recorded from the same mouse (heteroscedastic linear mixed-effects model on natural-log-transformed I/E ratios; likelihood-ratio test, χ^2^(1) = 23.31, *p* < 0.0001; S20C Fig). Because the spatial relationship of each recorded neuron to nearby Aβ plaques was not determined, this increased variability may reflect heterogeneity associated with local Aβ pathology, although this possibility remains speculative.

Interestingly, tetanic stimulation (100 pulses at 100 Hz) applied to Schaffer collaterals elicited long-term potentiation (LTP) of excitatory synapses between Schaffer collaterals and CA1 pyramidal neurons in acute dorsal hippocampal slices from both wild-type and *App*^*NL-G-F*^ mice. The magnitude of LTP did not differ significantly between genotypes after accounting for multiple slice recordings obtained from the same mouse (heteroscedastic linear mixed-effects model, *F*(1, 4) = 0.059, *p* = 0.821), and paired-pulse ratios after LTP induction were also comparable (*F*(1, 4) = 0.289, *p* = 0.620; S21 Fig). These results provide no evidence of a detectable impairment in Schaffer collateral–CA1 LTP in 5-month-old *App*^*NL-G-F*^ mice, suggesting that altered hippocampal LTP is unlikely to account for the working-memory deficits observed at this age. Together, these results support the notion that the working-memory deficits detected by the DNMTP task and altered performance in the TCSD tasks in the Operant House are associated with synaptic alterations already present in the prefrontal cortex of 5-month-old *AppNL-G-F* mice.

## Discussion

In this study, we developed the Operant House, a custom-built, cost-effective, and programmable device designed for automated operant conditioning tests in individually housed mice within the home cages. Several low-cost, home-cage–mountable systems have been reported, including the ArduiPod Box [33] and Psibox [8]. These devices can be installed in standard home cages, but were developed for a more restricted set of operant paradigms than those considered here. Other systems, such as ROBucket [34] and DIY-NAMIC [35], support more complex operant paradigms, yet their input interfaces are fixed: the number of nose-poke ports is limited (≤3), lever-press and touchscreen-based tasks cannot be implemented, and they do not support aversive light stimulation for punishment or a dedicated behavioral camera for direct video monitoring. The ARTS system [7] is specialized for joystick-based tasks in rats that move along an inverted Y-shaped slot; therefore, its configuration differs substantially from conventional touchscreen- and lever-based operant paradigms.

In contrast, a key advantage of the Operant House is its compatibility with programmable, touchscreen-based task paradigms for higher cognitive testing that have been developed for non-human primate and rodent models over the past two decades [31]. By simply changing the program and the mask pattern of the LCD monitor, different touchscreen-based operant tasks can be implemented in the home cages while minimizing experimenter intervention. Conventional lever-press tasks can also be implemented by replacing the chamber wall. In a home-cage lever-press operant conditioning task, mice acquired the behavior rapidly within a single day (S13F Fig), and the time required to complete the first 30 lever presses was approximately eightfold shorter than that reported using a conventional non-home-cage operant apparatus [4] although differences in apparatus design and training procedures preclude a direct quantitative comparison. Furthermore, because the Operant House is equipped with an overhead camera, task performance and animal condition can be monitored directly and recorded for subsequent inspection; the recorded videos can also support downstream behavioral analyses (S2 Video) [30,36–38].

The Operant House uses open-source Python software that can run on either a laptop computer (version 1) or a low-cost single-board computer (version 2), such as a Raspberry Pi 4, and the source code, construction files, and documentation are freely available on the project website (https://shintaro18.github.io/). Importantly, behavioral validation should be distinguished from technical implementation: visual discrimination, lever pressing, DNMTP, and TCSD were evaluated in mice in the present study, and TUNL has been evaluated in a separate study [39], whereas the other available paradigms remain to be systematically validated in animals.

As proof of concept, we evaluated working-memory-related cognitive performance in mice using two tests in the Operant House: the DNMTP and TCSD tests. Spatial working memory in rodents is commonly assessed with the T-maze alternation test [40]. In this test, mice must choose between a previously visited arm and a goal arm, the latter offering a food reward. A simplified version of this task measures spontaneous alternations in open arms without blocking entries [41]. However, T-maze alternation tests may be confounded by spatial novelty preference, a passive form of short-term memory [42]. For example, T-maze rewarded alternation performance was unaffected by the loss of NMDA receptors in the prefrontal cortex, an area critical for working memory, whereas DMTP tasks with five visual cues and nose-poking revealed significant impairments [43]. Similarly, the DNMTP test in the Operant House, which utilizes three positions for visual cues and nose pokes, led to increased c-Fos expression in the prefrontal cortex following task completion. Furthermore, prefrontal cortex lesions impaired performance in both DNMTP and TCSD tasks (Fig 6O, 9C-H). Because the lesion experiments involved relatively small groups (n = 3–4 per group), these findings should be regarded as supportive evidence for prefrontal involvement and interpreted cautiously with respect to the magnitude of the lesion effects. Nevertheless, together with the c-Fos results, these findings support the involvement of the prefrontal cortex in performance of both tasks in the Operant House. The DNMTP task directly assesses working memory, whereas performance in the TCSD task likely reflects multiple cognitive processes, including working memory, behavioral flexibility, and response selection.

The behavioral performance obtained in the DNMTP and TCSD tasks using the Operant House was generally comparable to that previously reported using conventional non-home-cage operant conditioning chambers. For example, Kwak et al. used a similar DNMTP paradigm in a commercial operant conditioning apparatus (Campden Instruments Ltd, UK) and reported correct rates of approximately 85% and 67% at 3- and 9-s delays, respectively, in 10-week-old male C57BL/6J mice [3]. In comparison, 5-month-old female wild-type C57BL/6J mice tested in the Operant House achieved correct-response rates of approximately 70% at 3- and 12-s delays (Fig 7F). Although these values are broadly within a similar performance range, the two studies differed not only in delay duration and training procedures but also in the age and sex of the mice, precluding direct quantitative comparison of task performance.

Similarly, in the TCSD task, Clelland et al. performed an analogous paradigm in a conventional operant apparatus in which the distance between the two choice panels was either large or small, with the large-separation condition corresponding to that used in the present study [32]. Under this large-separation condition, 8-week-old female C57BL/6 mice required an average of 16 trials per reversal, whereas mice tested in the Operant House required only 14.8 trials on average (Fig 9E; averaged across three sessions). Importantly, Clelland et al. reported that habituation, shaping, and pre-training required approximately 26 days before TCSD testing [32], whereas the Operant House protocol required only 3 days for these preparatory procedures. These findings indicate similar reversal performance, together with a substantially shorter preparatory period in the Operant House protocol.

Two additional studies provide further points of comparison. In the lever-press paradigm, mice tested in the Operant House completed their first 30 lever presses approximately eightfold faster than mice tested using a conventional non-home-cage apparatus [4], although differences in apparatus design and training conditions should be considered. In addition, a conventional touchscreen-based location discrimination test detected working-memory impairments in 4- to 6-month-old male *App*^*NL-G-F*^ mice [30], consistent with the abnormalities detected in 5-month-old male *App*^*NL-G-F*^ mice using the TCSD task in the Operant House. This latter comparison is qualitative because the two studies used related but non-identical task designs.

Taken together, these comparisons with four previous studies indicate that the Operant House can produce behavioral performance broadly comparable to that obtained using conventional non-home-cage apparatuses, while shortening task acquisition or preparatory training in some paradigms. Because these were not head-to-head comparisons and the studies differed in task parameters, training criteria, age, sex, and genetic background, these findings suggest potential gains in experimental efficiency in some paradigms but should not be interpreted as evidence of general superiority of the Operant House over conventional systems.

A limitation of the present study is that behavioral testing in the Operant House was conducted under single-housing conditions. While this design enables automated and individually assigned training without interference from cage mates, prolonged single housing may influence animal welfare, stress, and behavior, and should therefore be taken into account when interpreting the results. Animal condition can be monitored through live and recorded video, but such monitoring should supplement rather than replace routine welfare assessments. Regular body-weight measurements and visual assessments of general appearance, grooming, posture, spontaneous activity, food and water intake, and signs of injury or distress are therefore important during prolonged individual housing. Because operant performance was reinforced by access to water, differences in motivational state related to thirst cannot be completely excluded. However, in six wild-type mice tested under the same programmed DNMTP water-access regimen, no significant group-level change in body weight was observed. Because actual water consumption was not directly measured and these body-weight data were obtained from independent experiments, individual or genotype-dependent differences in hydration or motivation remain possible. Future refinements of the protocol should nevertheless aim to minimize the duration of single housing and, where feasible, maintain sensory contact between animals while preserving the advantages of individualized home-cage testing.

A further limitation concerns the analyses restricted according to actual delay duration. Because actual delay duration was influenced by the animals’ behavior, selection based on this measure could retain different subsets of trials across experimental groups, particularly in the TCSD experiments. We therefore treated the delay-restricted analyses as sensitivity analyses rather than independent confirmatory tests; the primary conclusions are based on analyses including all trials.

In the present study, operant task sessions were conducted once a day, following conventional non-homecage operant conditioning paradigms. Future studies could examine whether optimizing session timing, trial number, or reward schedules during the animals’ active phase can shorten training while maintaining appropriate animal welfare.

The *App*^*NL-G-F*^ mouse, which is thought to more faithfully recapitulate human AD pathology because it lacks artifacts caused by protein overexpression, exhibits relatively mild neurological phenotypes, particularly at early stages. For example, most studies have reported normal performance in the Morris water maze [26,30,44] and context fear conditioning [27,28] at six months of age. In AD, however, working memory impairment is one of the earliest symptoms during the preclinical stage [37,38,45]. Studies using the Y-maze in six-month-old *App*^*NL-G-F*^ mice have yielded mixed results: some report impairments [26,27], whereas others do not [29]. Interestingly, studies showing no impairments [29] used male mice, while another study found deficits only in females [27]. Although the reasons remain unclear, AD is more common in women than in men, and the estimated lifetime risk has been reported to be approximately twice as high in women [46]. This epidemiological difference does not directly explain the sex-dependent phenotypes observed in the mouse model but provides a relevant context for further investigation. Furthermore, differences in the sex of the experimenter have been suggested as a factor influencing Y-maze performance [29]. Thus, the abnormalities detected exclusively in female *App*^*NL-G-F*^ mice by the DNMTP test in the Operant House, which is conducted without direct experimenter intervention, are particularly noteworthy. Working memory impairments have also been reported in male *App*^*NL-G-F*^ mice at 4–6 months of age using a non–home-cage–based location discrimination (LD) test, in which male animals (C57BL/6J) choose between two visual stimuli presented on the left and right sides of a touchscreen [30]. Consistent with this, the TCSD test in the Operant House, which requires mice to choose between two sides based on five visual stimuli, revealed abnormalities in male *App*^*NL-G-F*^ mice at 5 months of age. These findings suggest that the DNMTP and TCSD tasks may probe distinct aspects of working memory or partially dissociable cognitive domains, such as cognitive inflexibility. The DNMTP task places particular demands on retaining and updating information across a delay, whereas the TCSD task requires spatial discrimination among multiple visual stimuli and may place different demands on response selection and cognitive flexibility. The sex-dependent phenotypes may therefore reflect differences in the temporal or circuit-level progression of AD-related dysfunction between female and male *App*^*NL-G-F*^ mice, differences in the demands and sensitivity of the behavioral tasks, or a combination of these factors. Because neuropathological progression and circuit dysfunction were not directly compared between sexes in the present study, these possibilities remain speculative and require further experimental investigation.

Taken together, our results indicate that the Operant House enables automated detection of early cognitive deficits in *App*^*NL-G-F*^ mice within their individual home cages, including working-memory impairment detected by the DNMTP task. The Operant House may therefore provide an accessible and scalable platform for investigating subtle cognitive phenotypes in preclinical models of AD and other neurological and neuropsychiatric disorders.

## Supporting information

S1 Fig Main parts list. (PDF)

S2 Fig Tool list for fabrication. (PDF)

S3 Fig Exploded-view diagram of Operant House Version 2 (top) and summary of the differences between Versions 1 and 2 (bottom). (PDF)

S4 Fig Assembly instructions for the 3D-printed parts. (PDF)

S5 Fig A schematic illustration of the circuitry of the switching board that controls the LEDs and a servo. (PDF)

S6 Fig Assembly instructions for LED wiring. (PDF)

S7 Fig Assembly instruction for wiring the IR and green LEDs. (PDF)

S8 Fig Assembly instruction for servo, Arduino and ventilation fan wiring. (PDF)

S9 Fig Mounting instructions for the Raspberry Pi, Arduino, and switching board. (PDF)

S10 Fig Assembly instructions for the camera, ventilation fan, and LEDs. (PDF)

S11 Fig Wiring of each component and external appearance of the completed device. (PDF)

S12 Fig Setup instruction for the Raspberry Pi 4B. (PDF)

S13 Fig Retractable lever modules. (PDF)

S14 Fig Additional results from the DNMTP test in wild-type mice. (PDF)

S15 Fig Additional results from the DNMTP task in wild-type mice with prefrontal cortex lesions induced by ibotenic acid. (PDF)

S16 Fig Results of the DNMTP test using Operant House Version 2. (PDF)

S17 Fig Additional results of the DNMTP test in female *App*^*NL−G−F*^ mice. (PDF)

S18 Fig Male *App*^*NL−G−F*^ mice exhibited normal working memory in the DNMTP test at 5 months of age. (PDF)

S19 Fig Reduced presynaptic marker immunoreactivity within Aβ plaques in the mPFC of *App*^*NL-G-F*^ mice at 5 months of age. (PDF)

S20 Fig Increased variability in excitatory–inhibitory balance in *App*^*NL-G-F*^ mice at 5 months of age. (PDF)

S21 Fig No detectable alteration in hippocampal long-term potentiation (LTP) in 5-month-old *App*^*NL-G-F*^ mice. (PDF)

S1 Video Automated detection of nose pokes during the VD2 session using Operant House Version 2. (AVI)

S2 Video Analysis of behavioral patterns using the built-in camera of the Operant House and DeepLabCut. (AVI)
