# Supplementary material for: Operant House: A versatile open-source platform for automated operant conditioning testing of mice in home cages
Source: PLoS One. 2026 Oct 13;21(10):e0360626. doi: 10.1371/journal.pone.0360626 (PMC13660919; doi:10.1371/journal.pone.0360626)
Supplement: Supplementary file 1 [file pone.0360626.s001.pdf]

Usage	Name	Vendor	Cost ($US)
Main computer	Raspberry Pi 4, Model B, 8GB	Amazon	64.59
Data storage	SanDisk 400GB Ultra microSDXC	Amazon	39.99
Microcomputer	Arduino Uno REV3 (A000066)	Amazon	27.60
Touch & Operation monitor	Haiway 10.1-inch LCD HDMI Monitor (1366x768 pixel resolution)	Amazon	63.72
Behavior and nosepoke detection camera	ELP USB Camera (FoV: 157°) (ELP-USBFHD01M-L21)	Amazon	45.91
Illumination for light-cycle and punishment	5 mm white LED (LEDDC-5WHI)	DigiKey	0.08 x 4 = 0.32
Illumination for nosepoke detection	5 mm infrared LED (HR5P-N4FA-00000)	DigiKey	0.79 x 5 = 3.95
Illumination for reward cue	5 mm green LED (DC3.1V 20mA, 517nm, 1650 mcd, 30° view angle, clear; CHANZON)	Amazon	6.99
Chamber ventilation	40x40x10 mm brushless cooling fan (DC12V; UMLIFE)	Amazon	8.99
Water supply control	Water resistant servo (DS3225MG)	Amazon	16.98
Water supply	8 oz water bottle (PC8HT)	Ancare	6.60
Water supply	Ball point drinking tube (TD-100)	Ancare	1.62

S1 Fig. Main parts list
