# Supplementary material for: Operant House: A versatile open-source platform for automated operant conditioning testing of mice in home cages
Source: PLoS One. 2026 Oct 13;21(10):e0360626. doi: 10.1371/journal.pone.0360626 (PMC13660919; doi:10.1371/journal.pone.0360626)
Supplement: Supplementary file 2 [file pone.0360626.s002.pdf]

- Solder / Soldering iron
- Nippers
- Instant glue
- Epoxy glue
- Masking tape
- Double sided tape
- Hand drill & 5 mm drill bit
- Phillips-head screwdriver
- FDM 3D printer

S2 Fig. Tool list for fabrication.
