# Supplementary material for: Operant House: A versatile open-source platform for automated operant conditioning testing of mice in home cages
Source: PLoS One. 2026 Oct 13;21(10):e0360626. doi: 10.1371/journal.pone.0360626 (PMC13660919; doi:10.1371/journal.pone.0360626)
Supplement: Supplementary file 3 [file pone.0360626.s003.pdf]

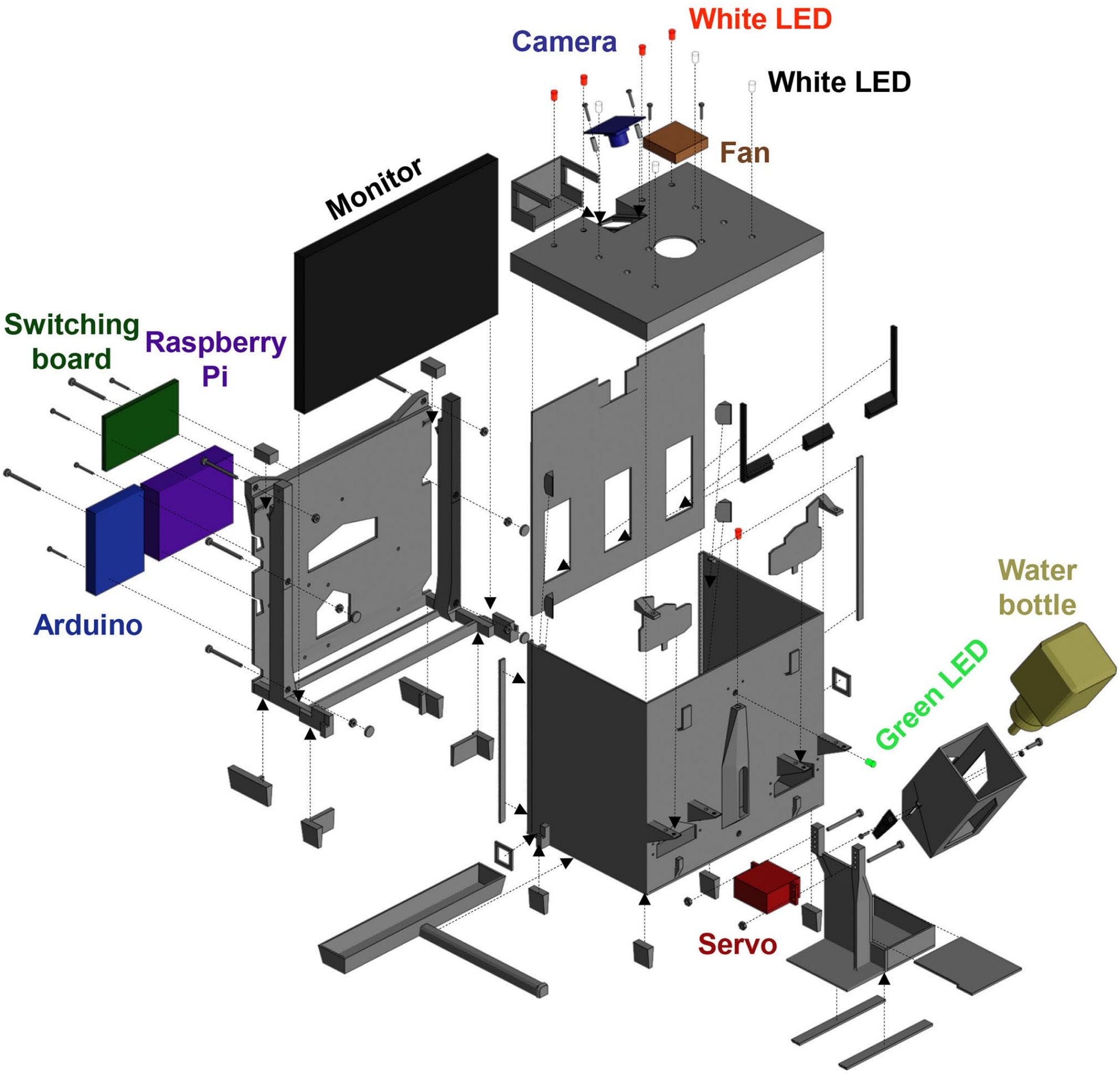

Feature	Version 1	Version 2
Main material	PVC	PLA
Control computer	Laptop computer	Raspberry Pi 4B or laptop computer
Nose-poke detection	IR sensor only	IR camera or IR sensor
Lever module	Not supported	Supported
Miniscope recording	Not supported	Supported

S3 Fig. Exploded-view diagram of Operant House Version 2 (top) and summary of the differences between Versions 1 and 2 (bottom).
