# Supplementary material for: Operant House: A versatile open-source platform for automated operant conditioning testing of mice in home cages
Source: PLoS One. 2026 Oct 13;21(10):e0360626. doi: 10.1371/journal.pone.0360626 (PMC13660919; doi:10.1371/journal.pone.0360626)
Supplement: Supplementary file 4 [file pone.0360626.s004.pdf]

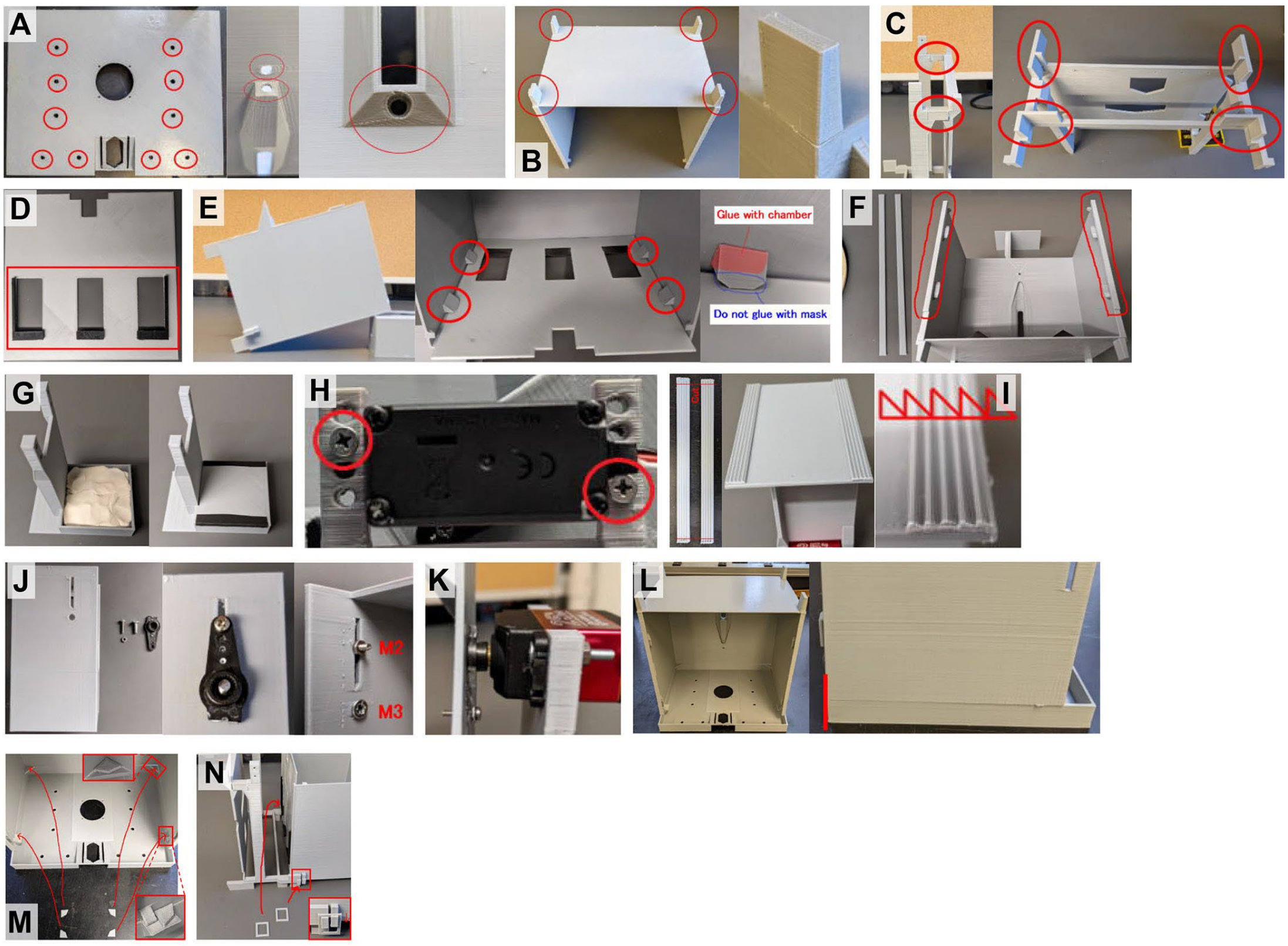

S4 Fig. Assembly instruction for the 3D-printed parts.

(**A**) Enlarge the LED mounting holes using a 5 mm drill bit. (**B**) Glue the legs to the underside of the chamber. (**C**) Glue the beams and legs to the chamber body. (**D**) Glue the mask hole bottoms to the touchscreen mask. (**E**) Tilt the chamber and place the mask on the front of the chamber. Glue four mask holders to the chamber to hold the mask in place. Do not glue the mask itself, as it must remain replaceable by sliding. (**F**) If a camera will be used for panel-touch detection, attach the spacers to the chamber using double-sided tape. (**G**) Fill the interior of the water arm with clay or another heavy material (e.g., wheel weights) to act as a counterweight, then attach the lid and secure it with tape. (**H**) Fix the servo motor to the water bottle arm using M3 × 20 mm screws and nuts. (**I**) Trim the ends of the water-arm stoppers with nippers. Glue them to the underside of the water arm, ensuring correct orientation before bonding. (**J**) Fix the servo arm to the bottle holder using M3 × 8 mm and M2 × 20 mm screws as shown (if an M3 × 8 mm screw is unavailable, cut an M3 × 20 mm screw to the required length using nippers). (**K**) Insert the servo arm onto the servo rotation axis and secure it with an M3 × 8 mm screw. (**L**) Place the chamber on the inverted roof. Ensure that the front sides of the chamber and roof are aligned, as indicated by the red line in the photograph. (**M**) Glue the stop blocks to the lid to prevent displacement. Do not bond the stop blocks directly to the chamber body so that the roof remains removable. (**N**) Verify that the square rocker can be inserted properly to connect the chamber and the main body.
