# Supplementary material for: Operant House: A versatile open-source platform for automated operant conditioning testing of mice in home cages
Source: PLoS One. 2026 Oct 13;21(10):e0360626. doi: 10.1371/journal.pone.0360626 (PMC13660919; doi:10.1371/journal.pone.0360626)
Supplement: Supplementary file 5 [file pone.0360626.s005.pdf]

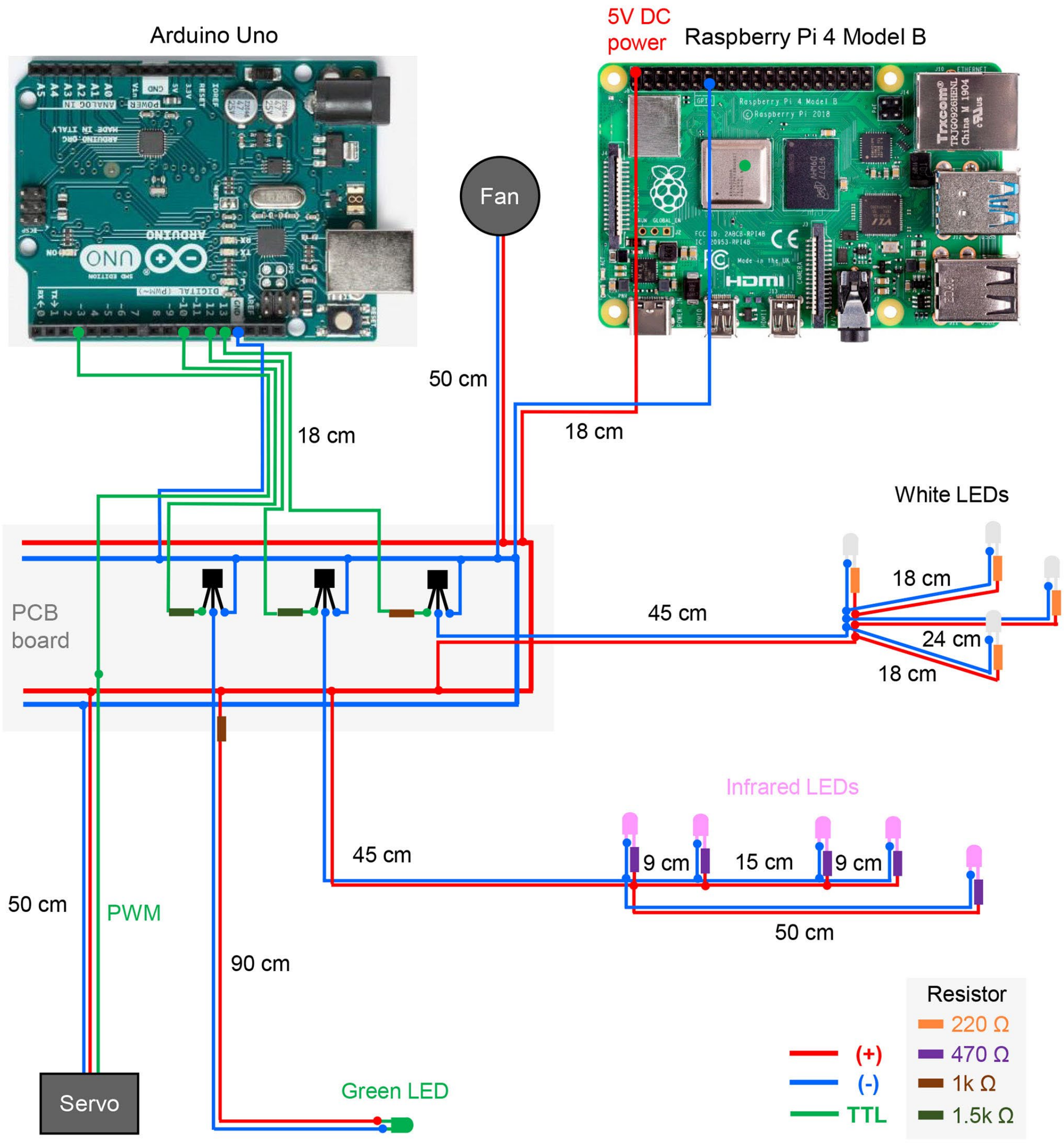

S5 Fig. A schematic illustration of the circuitry of the switching board that controls the LEDs and a servo.
