# Supplementary material for: Operant House: A versatile open-source platform for automated operant conditioning testing of mice in home cages
Source: PLoS One. 2026 Oct 13;21(10):e0360626. doi: 10.1371/journal.pone.0360626 (PMC13660919; doi:10.1371/journal.pone.0360626)
Supplement: Supplementary file 6 [file pone.0360626.s006.pdf]

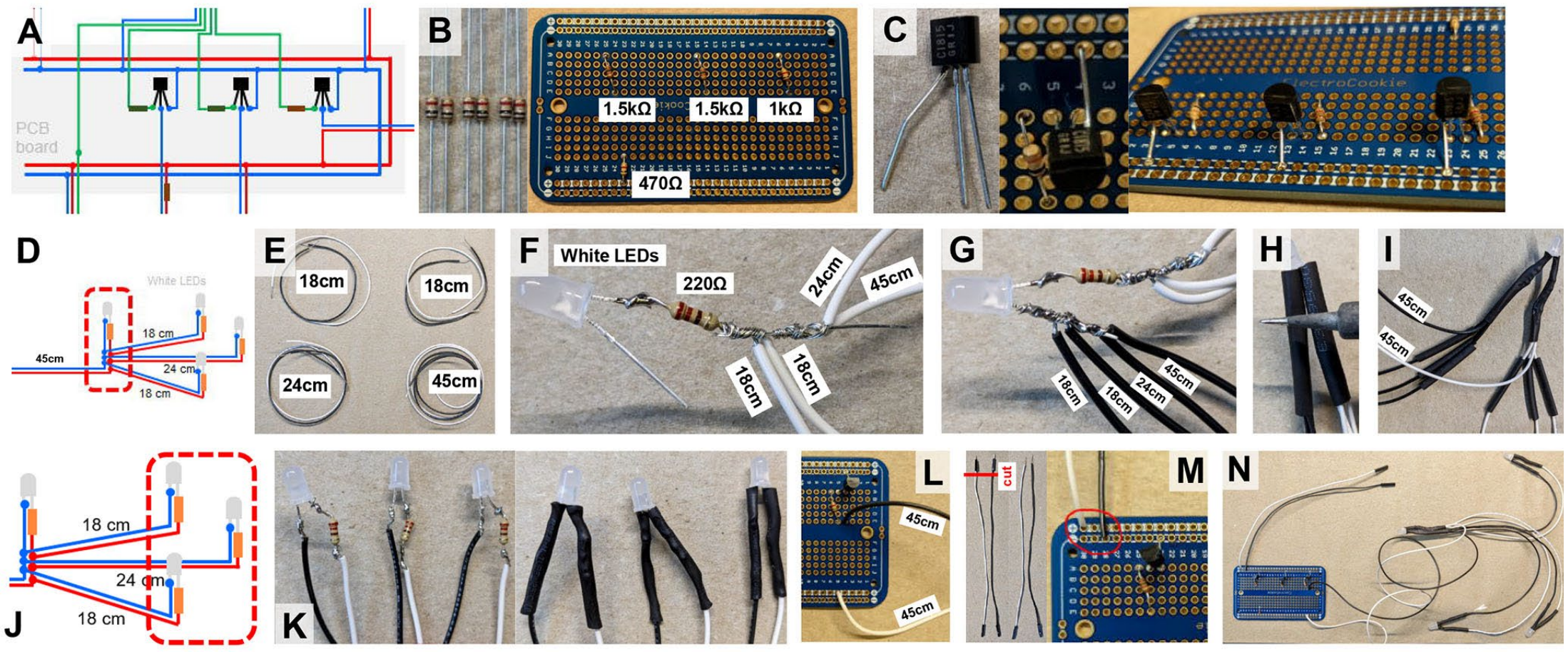

S6 Fig. Assembly instructions for LED wiring.

(**A**) Schematic diagram of the switching board. (**B**) Solder the resistors as shown. (**C**) Bend the emitter leads as illustrated and solder them onto the PCB. (**D**) Schematic diagram of the white LED circuit. (**E**) Cut the electrical wires to the specified lengths. (**F**) Twist a 220 Ω resistor onto the anode lead (longer lead) of the LED for temporary fixation and solder it. Then solder the prepared wires to the opposite lead of the resistor. (**G**) Solder the other wires to the cathode of the LED. (**H**) Cover the exposed joints with heat-shrink tubing and apply heat (e.g., using the shaft of a soldering iron or a heat gun) to shrink and secure the insulation. (**I**) Slide the 3 mm heat-shrink tubing onto the wires. (**J**) White LEDs to be wired. (**K**) Solder 220 Ω resistors to the white LEDs. Solder 18 or 24 cm wires to the opposite lead of the resistors and cover the joints with heat-shrink tubing. (**L**) Solder the 45 cm wires to the PCB as shown. (**M**) Prepare jumper wires with female connectors and solder them to the PCB. (**N**) Assembled appearance of the circuit.
