# Supplementary material for: Operant House: A versatile open-source platform for automated operant conditioning testing of mice in home cages
Source: PLoS One. 2026 Oct 13;21(10):e0360626. doi: 10.1371/journal.pone.0360626 (PMC13660919; doi:10.1371/journal.pone.0360626)
Supplement: Supplementary file 7 [file pone.0360626.s007.pdf]

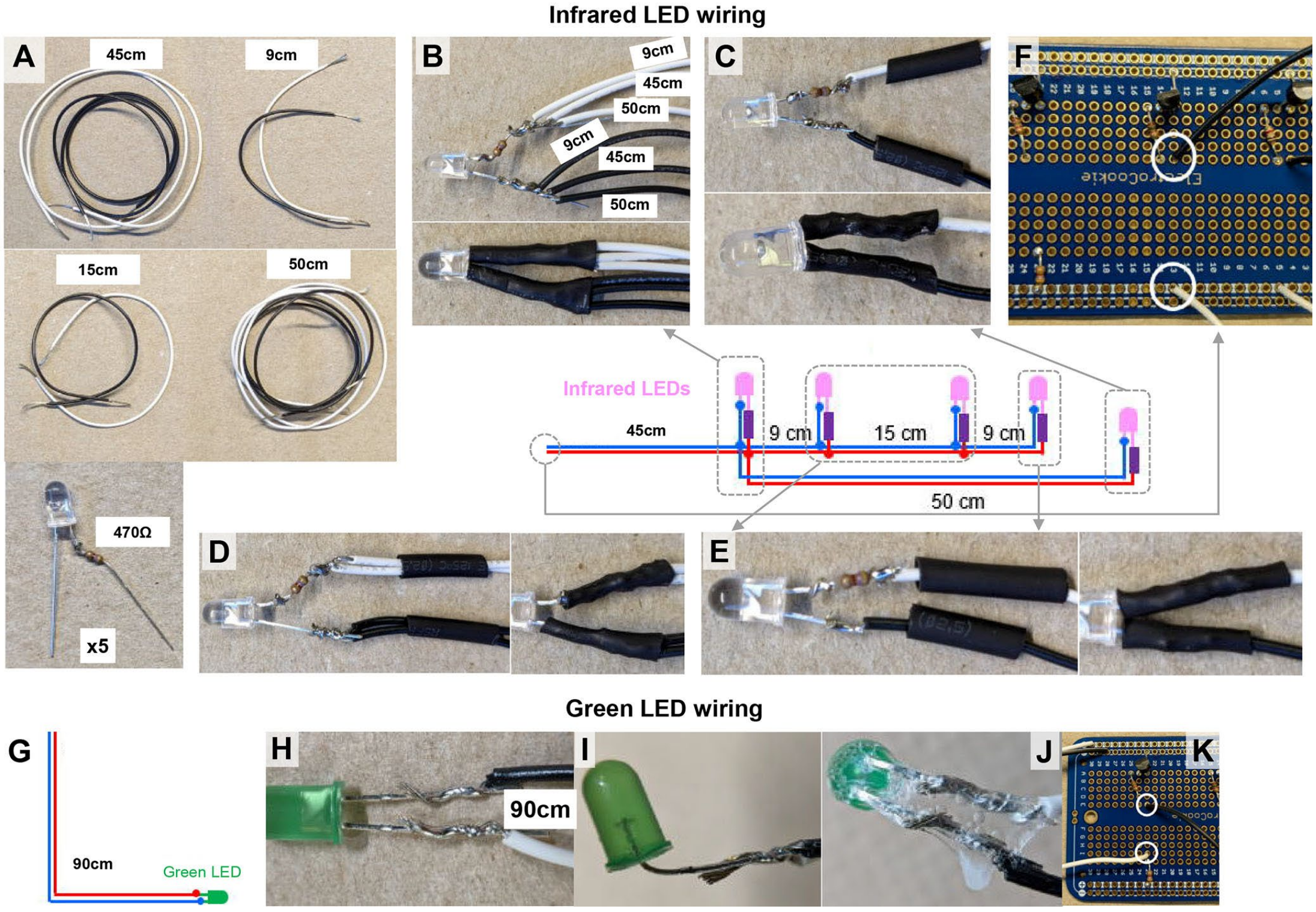

S7 Fig. Assembly instruction for wiring the IR and green LEDs.

(**A**) Prepare wires of the specified lengths and five IR LEDs, each soldered to a 470 Ω resistor. (**B–E**) Solder the LEDs and wires as specified. (**F**) Solder the 45 cm wires to the board. (**G**) Schematic diagram of the circuit. (**H**) Solder a green LED to a pair of 90 cm wires. (**I**) Bend the LED leads to approximately 80°. (**J**) Cover the leads and exposed wires with epoxy adhesive to provide waterproofing and mechanical reinforcement. (**K**) Solder the wires to the board.
