# Supplementary material for: Operant House: A versatile open-source platform for automated operant conditioning testing of mice in home cages
Source: PLoS One. 2026 Oct 13;21(10):e0360626. doi: 10.1371/journal.pone.0360626 (PMC13660919; doi:10.1371/journal.pone.0360626)
Supplement: Supplementary file 8 [file pone.0360626.s008.pdf]

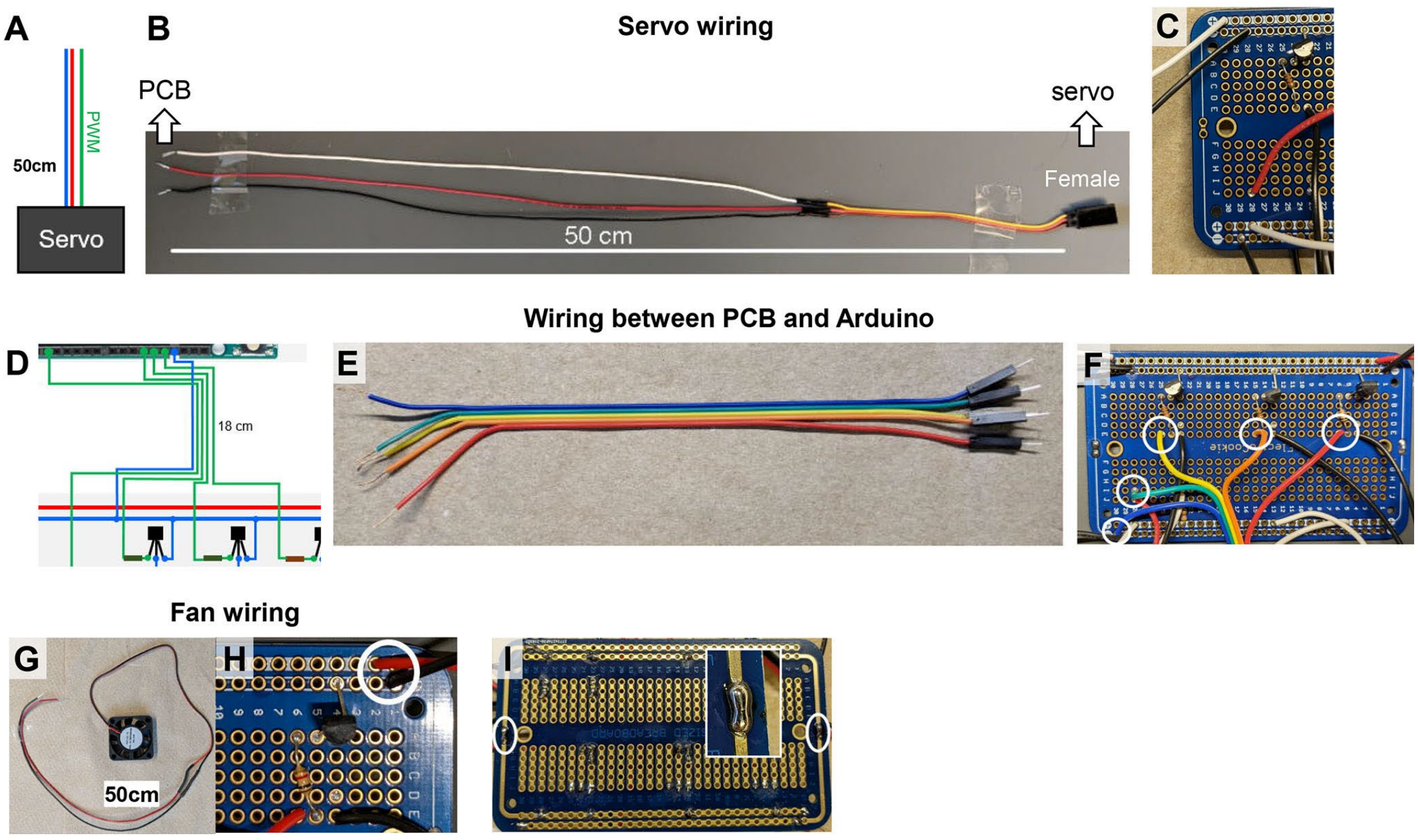

S8 Fig. Assembly instruction for servo, Arduino and ventilation fan wiring.

(**A**) Schematic diagram of the circuit. (**B**) Remove the male connector from the servo cable and extend the cable to 50 cm. (**C**) Solder the extended servo cable wires to the PCB. In the example shown, black = ground, white = 5 V, and red = PWM control signal. Refer to the manufacturer's manual or website to confirm the wiring color code. (**D**) Circuit diagram showing the connections between the Arduino and the PCB. (**E**) Prepare five jumper wires with male connectors. (**F**) Solder each jumper wire to the corresponding line on the board as shown. (**G**) Extend the fan wires to 50 cm. (**H**) Solder the fan wires to the power rails on the board. (**I**) If the board has separate power rails, bridge them with solder to supply power to both rails.
