# Supplementary material for: Operant House: A versatile open-source platform for automated operant conditioning testing of mice in home cages
Source: PLoS One. 2026 Oct 13;21(10):e0360626. doi: 10.1371/journal.pone.0360626 (PMC13660919; doi:10.1371/journal.pone.0360626)
Supplement: Supplementary file 9 [file pone.0360626.s009.pdf]

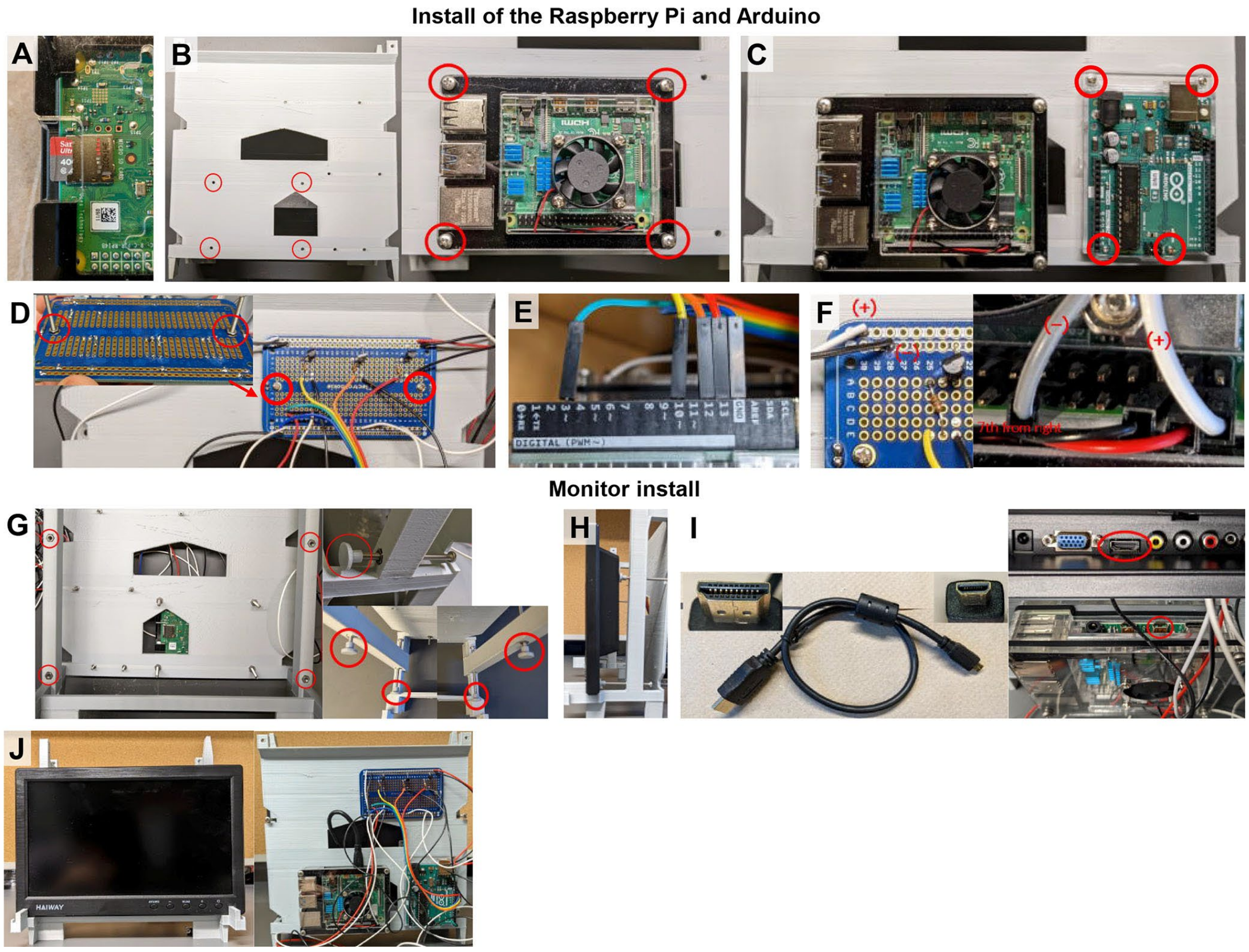

S9 Fig. Mounting instructions for the Raspberry Pi, Arduino, and switching board.

(**A**) Insert the SD card containing the pre-installed Raspberry Pi OS into the SD card slot of the Raspberry Pi. (**B**) Mount the Raspberry Pi onto the chamber body using M3 × 50 mm screws and M3 nuts. If the holes are undersized, enlarge them with a 3 mm drill. (**C**) Mount the Arduino onto the chamber body using M2 × 20 mm screws. (**D**) Insert the M2 × 20 mm screws through the switching board and secure them with nuts. Mount the board onto the chamber body and fasten it in place with additional nuts. (**E**) Connect jumper wires from the switching board to the Arduino as follows: GND – ground of the switching board; Ch13 – ceiling illumination; Ch12 – infrared LED; Ch11 – sync signal (if necessary); Ch10 – cue LED; Ch3 – servo motor (PWM). (**F**) Connect the power cable from the switching board to the Raspberry Pi (GND and 5 V output). (**G**) Insert M3 nuts into the recessed hollows on the chamber body. Thread M3 × 50 mm screws through the nuts and attach the monitor push adapters. (**H**) Mount the monitor and tighten the screws until the monitor is secured in the vertical position. (**I**) Connect the monitor to the Raspberry Pi using an HDMI cable (Mini HDMI to HDMI). (**J**) Appearance of the assembly after mounting.
