# Supplementary material for: Operant House: A versatile open-source platform for automated operant conditioning testing of mice in home cages
Source: PLoS One. 2026 Oct 13;21(10):e0360626. doi: 10.1371/journal.pone.0360626 (PMC13660919; doi:10.1371/journal.pone.0360626)
Supplement: Supplementary file 10 [file pone.0360626.s010.pdf]

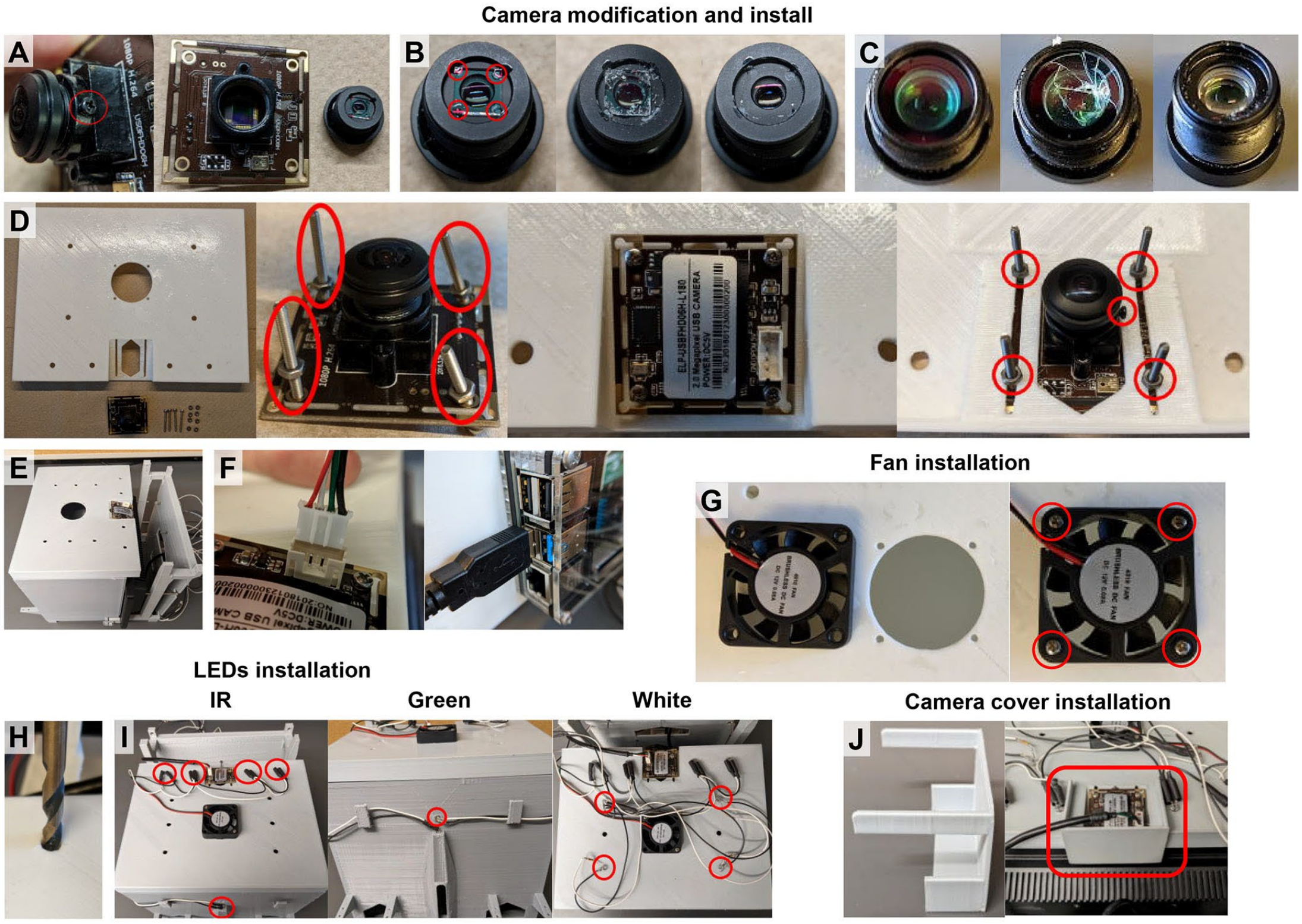

S10 Fig. Assembly instructions for the camera, ventilation fan, and LEDs.

(**A**) Loosen the side screw and rotate the lens unit to detach it. (**B**) The square glass element is an infrared filter. Break its four edges with nippers to detach it from the lens unit, then reattach the lens unit to the camera body. (**C**) If the IR filter is circular, gently crack it using the tips of the nippers and remove it. (**D**) Position the M2 × 20 mm screws and M3 nuts as shown (left, red circles). Then mount the camera onto the lid and secure it with M2 nuts (right). Reinsert the side screw. (**E**) Place the lid on the chamber together with the touchscreen mask and check for any interference between the camera and the mask. (**F**) Connect the camera to a USB 3.0 port (blue) on the Raspberry Pi. (**G**) Attach the ventilation fan to the lid using M2 × 20 mm screws. (**H**) Use a 5 mm drill bit to remove burrs from the holes to ensure proper fitting of the LEDs. (**I**) Insert each LED into the specified holes. If a hole is too large to secure the LED, apply a small amount of adhesive to the inner wall of the hole to reduce its diameter. (**J**) Test whether the camera cover fits properly on the roof. Do not apply adhesive, as the cover must remain removable.
