# Supplementary material for: Operant House: A versatile open-source platform for automated operant conditioning testing of mice in home cages
Source: PLoS One. 2026 Oct 13;21(10):e0360626. doi: 10.1371/journal.pone.0360626 (PMC13660919; doi:10.1371/journal.pone.0360626)
Supplement: Supplementary file 11 [file pone.0360626.s011.pdf]

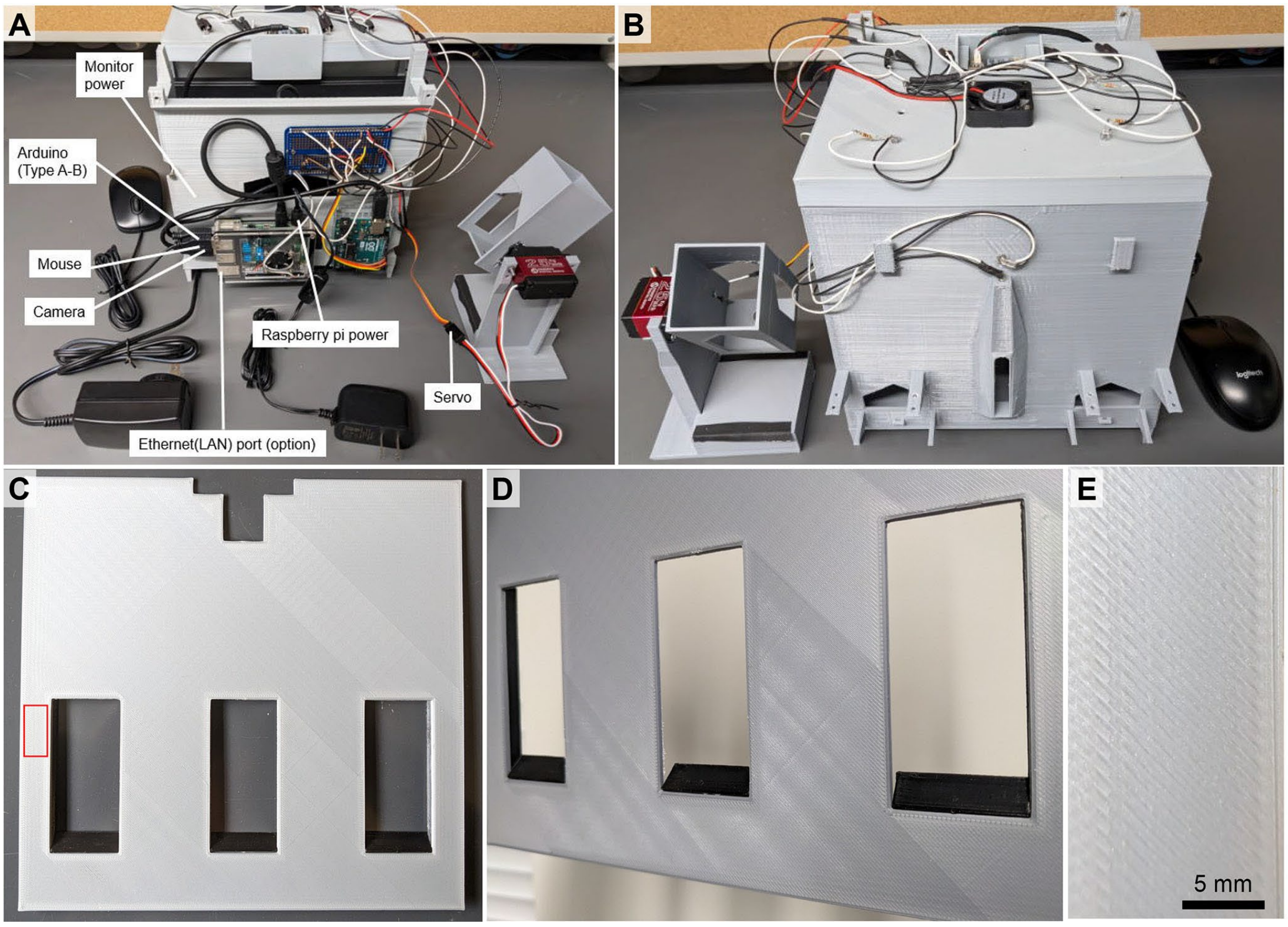

S11 Fig. Wiring of each component and external appearance of the completed device.
(**A**) Connect Raspberry Pi and Arduino with Type A to B USB cable. Connect USB mouse and the camera. Connect the power cable for the monitor and the Raspberry Pi. Connect the servo cable to the servo motor. Connect the ethernet cable to the Raspberry Pi. (**B**) Front view of the Operant House. (**C-D**) Appearance of the mask. The surface facing the inside of the chamber (**C**) and the black parts that function as a background for nosepoke detection with an IR camera. (**E**) Enlarged view of the mask surface outlined by the red rectangle in panel C.
