# Supplementary material for: Operant House: A versatile open-source platform for automated operant conditioning testing of mice in home cages
Source: PLoS One. 2026 Oct 13;21(10):e0360626. doi: 10.1371/journal.pone.0360626 (PMC13660919; doi:10.1371/journal.pone.0360626)
Supplement: Supplementary file 12 [file pone.0360626.s012.pdf]

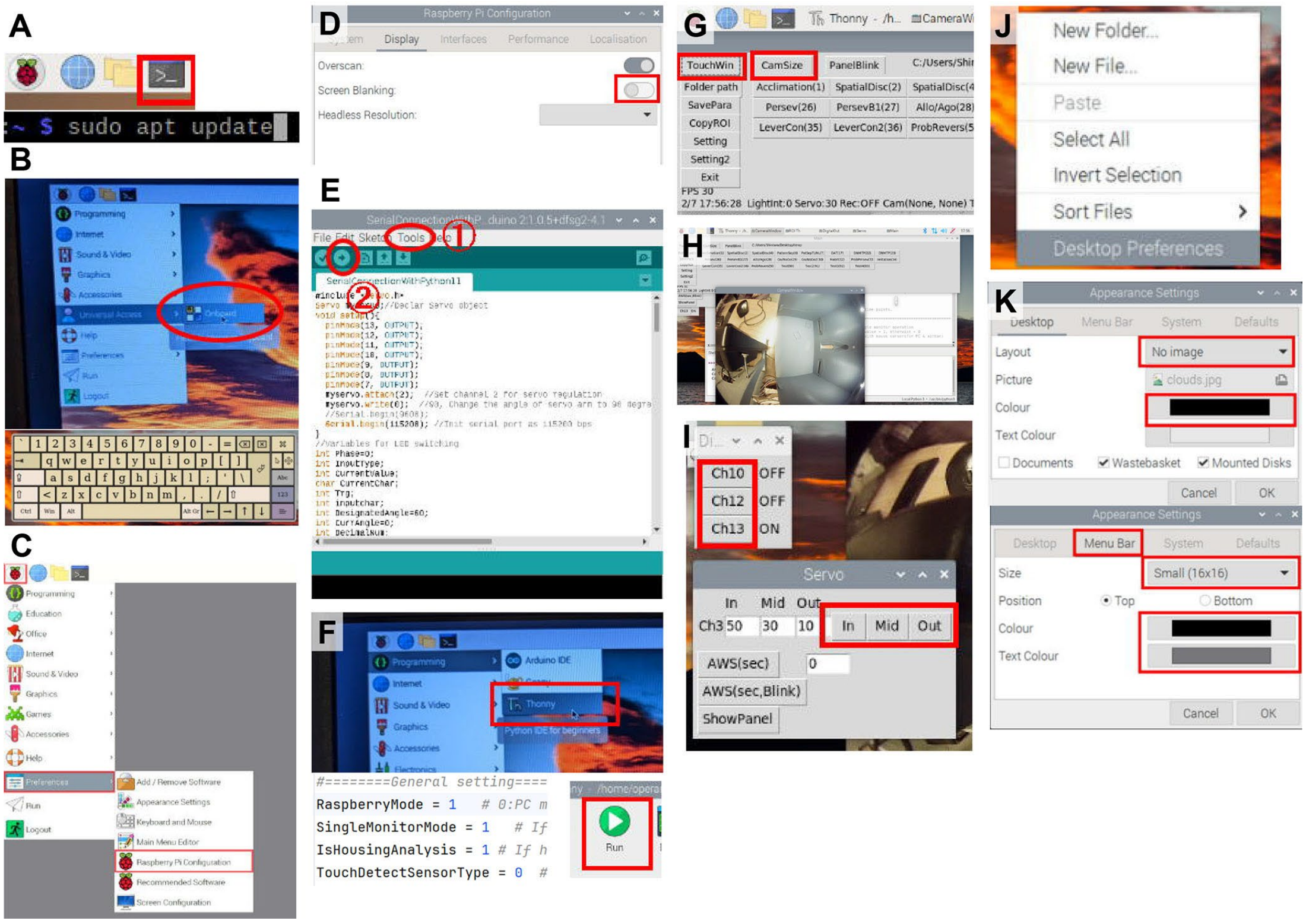

S12 Fig. Setup instruction for the Raspberry Pi 4B.

1. Install the OS.

   Install Raspberry Pi OS (32-bit, released on September 22, 2022) onto the SD card.

2. Initial connections and startup.

   Connect a keyboard and mouse. Establish a Wi-Fi connection; if Wi-Fi is not available, connect an Ethernet cable. Then turn on the power.

3. Install the software keyboard.

   Open a console window and enter `sudo apt update`, then press Enter (**A**). Next, run the following commands sequentially:

   ```
   sudo apt upgrade
   sudo apt install onboard
   ```

   The software keyboard can be launched from the Start menu (**B**).

4. Disable screen sleep.

   Open the Raspberry Pi Configuration tool (**C**) and turn off *Screen Blanking* (**D**).

5. Install OpenCV.

In the console, run the following command (due to its length, copy–paste from the online instructions is recommended):

```
sudo apt-get install build-essential cmake pkg-config libjpeg-dev libtiff5-dev libjasper-dev libpng-dev libavcodec-dev libavformat-dev libswscale-dev libv4l-dev libxvidcore-dev libx264-dev libfontconfig1-dev libcairo2-dev libgdk-pixbuf2.0-dev libpango1.0-dev libgtk2.0-dev libgtk-3-dev libatlas-base-dev gfortran libhdf5-dev libhdf5-serial-dev libhdf5-103 python3-pyqt5 python3-dev -y
```

Then run:

```
pip install opencv-python==4.5.3.56
pip install -U numpy
```

6. Install the Arduino IDE.

   Install the Arduino IDE with the following command:

   ```
   sudo apt install arduino
   ```

7. Install the Operant House software.

   Download the Operant House software from the project website and extract it to the desktop. Open the Arduino program located at

   ```
   OperantHouse/SerialConnectionWithPython*/SerialConnectionWithPython*.ino
   ```

   using the Arduino IDE. Select the appropriate port under **Tools → Port**, then upload the program by clicking the right-arrow button (**E**).

8. Run the Operant House software.

   Open Thonny (**F**) and confirm that the parameters are set as indicated in the general settings (**F**). Hide the touch window by clicking the TouchWin button, then enlarge the camera window by pressing the CamSize button (**G**). Verify that the camera is functioning properly (**H**). Test the ceiling, cue, and infrared LEDs, and confirm servo motor control using the test buttons (**I**).

9. Change the desktop background color.

   Right-click on the desktop, select Desktop Preferences (**J**), and adjust the settings as shown (**K**).

10. Set up remote desktop access.

    Install RealVNC and/or TeamViewer for remote desktop access. RealVNC is free for local area network (LAN) connections (e.g., when the Operant House and office computer are in the same building), but internet-based connections require a paid license. TeamViewer is also a paid service; however, the free license allows brief connections of a few minutes per access.
