# Supplementary material for: Operant House: A versatile open-source platform for automated operant conditioning testing of mice in home cages
Source: PLoS One. 2026 Oct 13;21(10):e0360626. doi: 10.1371/journal.pone.0360626 (PMC13660919; doi:10.1371/journal.pone.0360626)
Supplement: Supplementary file 13 [file pone.0360626.s013.pdf]

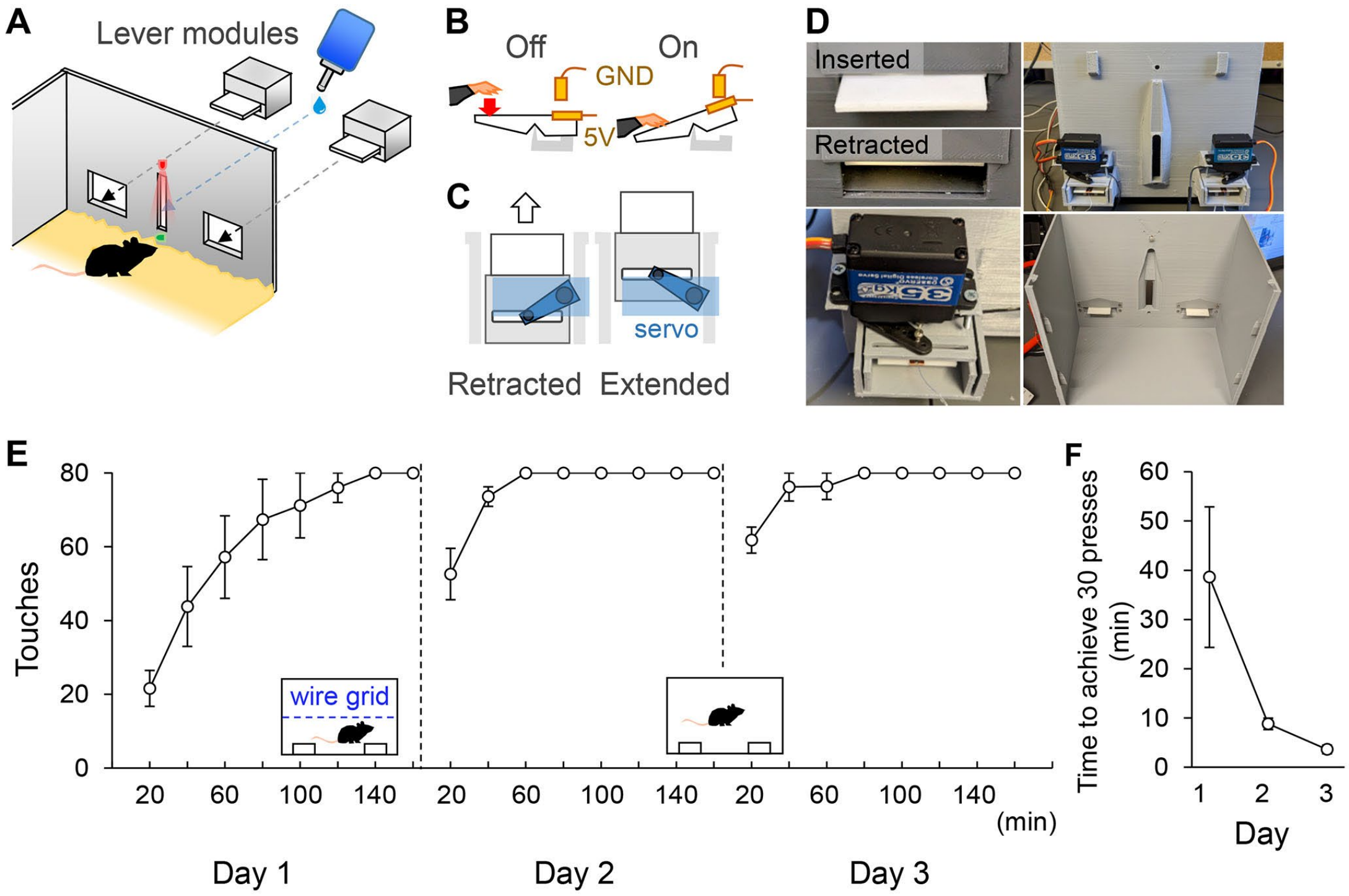

S13 Fig. Retractable lever modules.

(**A–C**) Design and schematic representation of the retractable lever module. Two or more lever modules can be installed in the default chamber (**A**). The lever operates using a seesaw-like mechanism and returns to its original position by gravity (**B**), requiring minimal force to press (< 75 mg). A servo motor allows the lever modules to slide back and forth (**C**). (**D**) Photographs of the lever modules: close-up views of the extended and retracted lever (upper left), an external view of the lever modules (upper right), a detailed view of the module exterior (bottom left), and a view of the levers from inside the chamber (bottom right). (**E, F**) Lever-press performance in wild-type mice (*n* = 5). The number of lever presses in each time bin (**E**) and the elapsed time to achieve 30 presses (**F**).
