# Supplementary material for: Operant House: A versatile open-source platform for automated operant conditioning testing of mice in home cages
Source: PLoS One. 2026 Oct 13;21(10):e0360626. doi: 10.1371/journal.pone.0360626 (PMC13660919; doi:10.1371/journal.pone.0360626)
Supplement: Supplementary file 14 [file pone.0360626.s014.pdf]

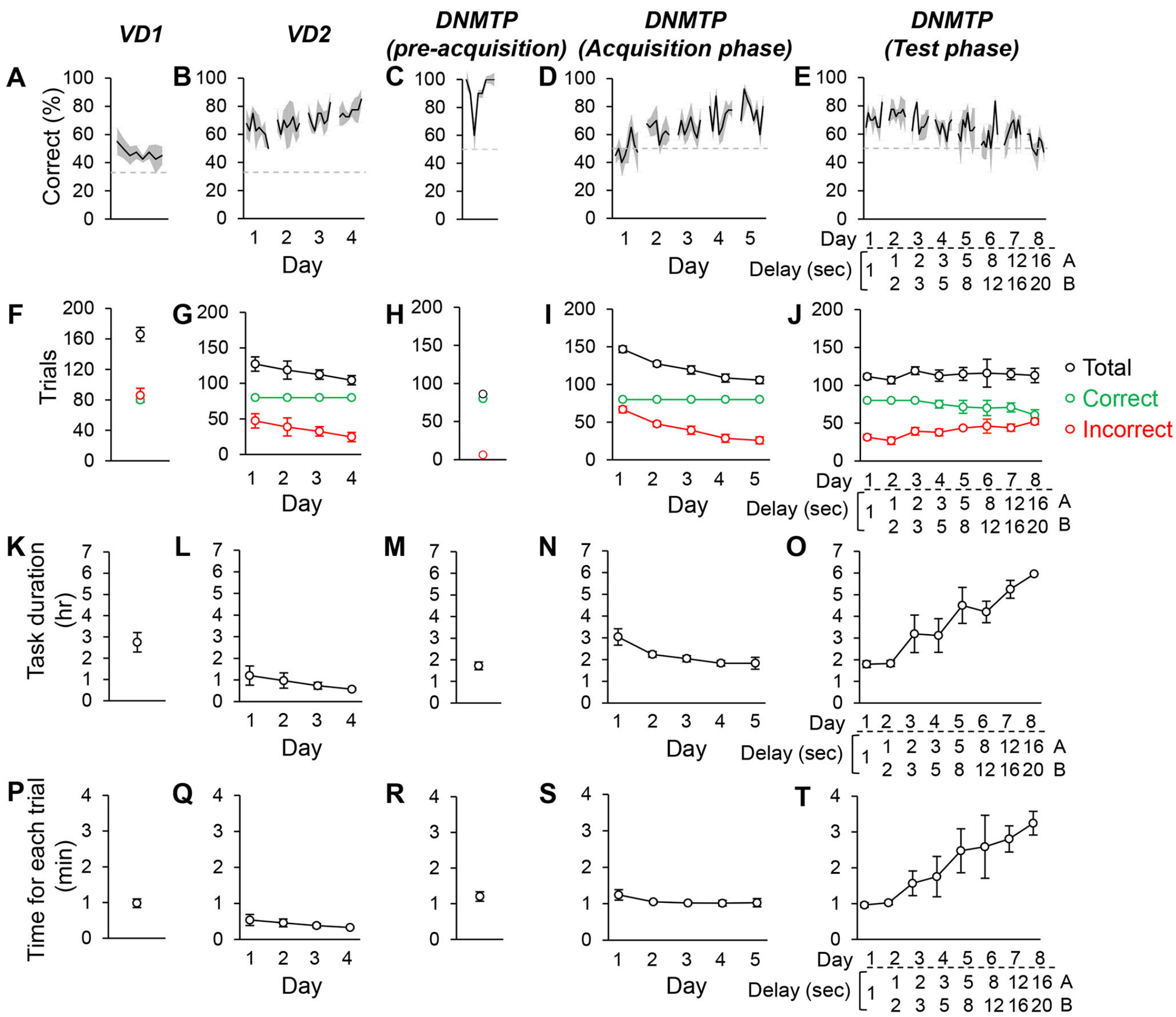

S14 Fig. Additional results from the DNMTP test in wild-type mice.

(**A-E**) Correct rates for each 10-trial bin across tasks. Graphs display results from the first 8 bins of each day. The average correct rate within each task and day remained relatively constant. (**F-J**) Total (black circles), correct (green), and incorrect (red) trial counts for each day. (**K-O**) Task durations for each day. (**P-T**) Average time elapsed per trial.
