# Supplementary material for: Operant House: A versatile open-source platform for automated operant conditioning testing of mice in home cages
Source: PLoS One. 2026 Oct 13;21(10):e0360626. doi: 10.1371/journal.pone.0360626 (PMC13660919; doi:10.1371/journal.pone.0360626)
Supplement: Supplementary file 15 [file pone.0360626.s015.pdf]

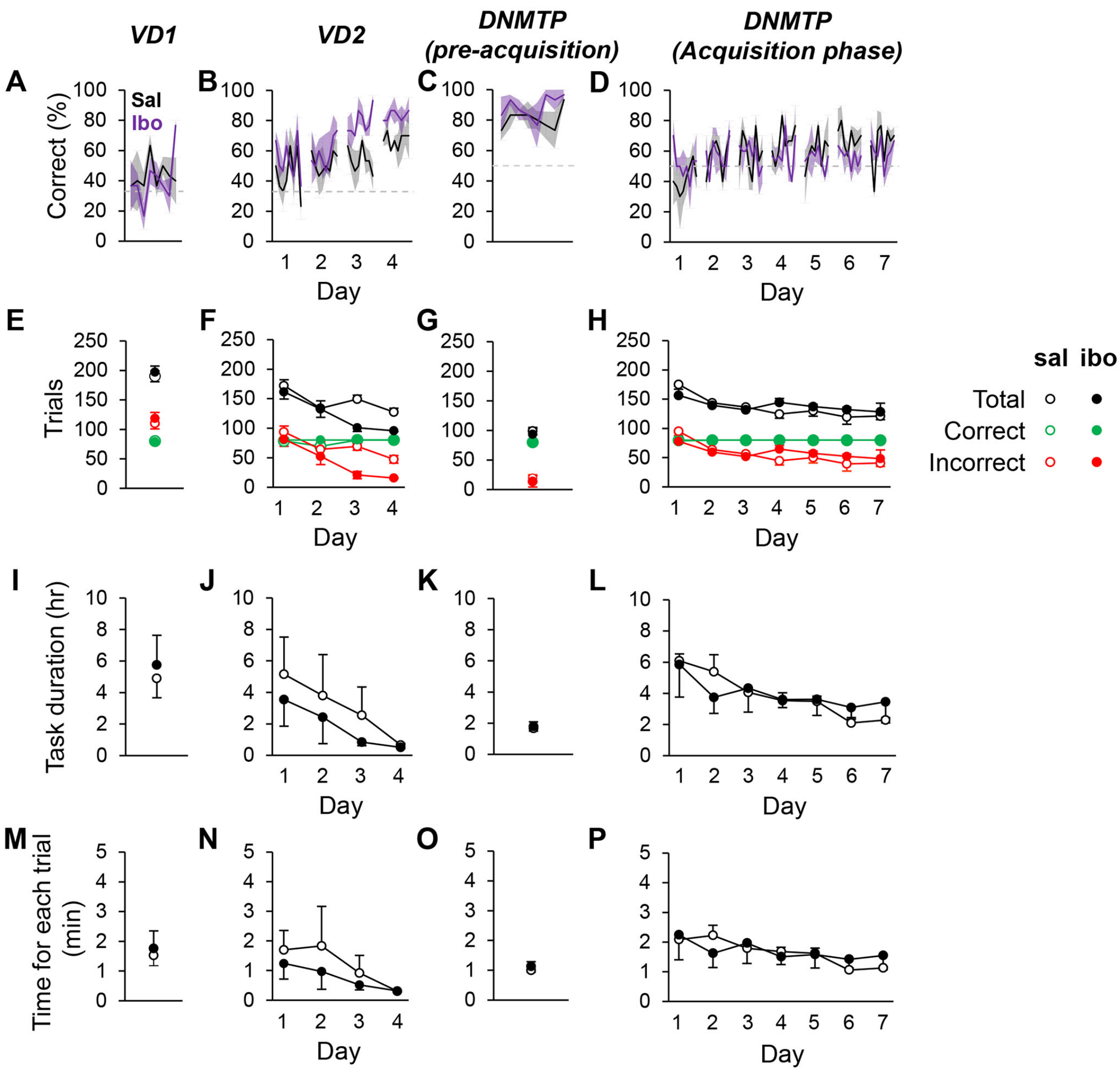

S15 Fig. Additional results from the DNMTP task in wild-type mice with prefrontal cortex lesions induced by ibotenic acid.

(**A-D**) Correct rates for each 10-trial bin across tasks in saline- and ibotenic acid-treated mice. Graphs show results from the first 8 bins of each day. (**E-H**) Total (black circles), correct (green), and incorrect (red) trial counts for each day. (**I-L**) Task duration for each day. (**M-P**) Average time elapsed per trial.
