# Supplementary material for: Operant House: A versatile open-source platform for automated operant conditioning testing of mice in home cages
Source: PLoS One. 2026 Oct 13;21(10):e0360626. doi: 10.1371/journal.pone.0360626 (PMC13660919; doi:10.1371/journal.pone.0360626)
Supplement: Supplementary file 16 [file pone.0360626.s016.pdf]

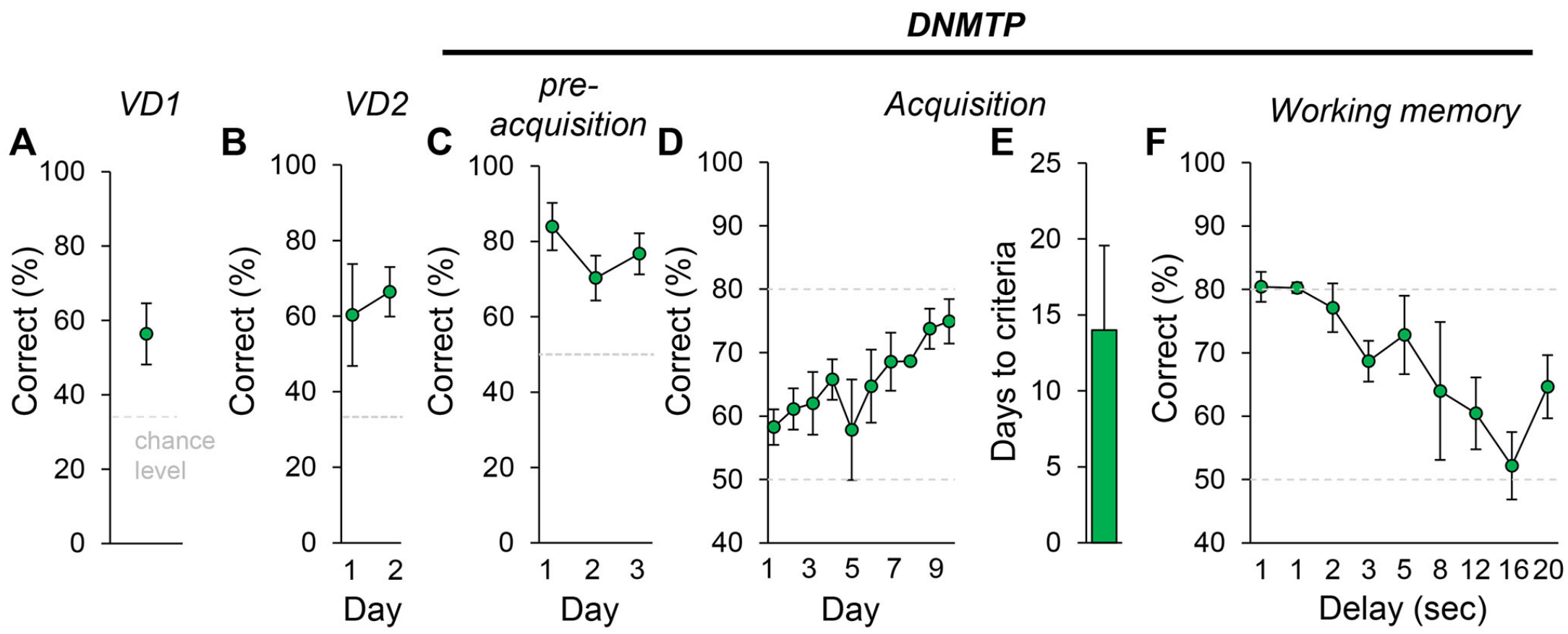

S16 Fig. Results of the DNMTP test using Operant House Version 2.

(**A-B**) Results of visual discrimination tasks. (**C**) Performance during the pre-acquisition phase of the DNMTP test. (**D-F**) Results of the acquisition and working memory phases of the DNMTP test. Data were obtained from wild-type mice (*n* = 3).
