# Supplementary material for: Operant House: A versatile open-source platform for automated operant conditioning testing of mice in home cages
Source: PLoS One. 2026 Oct 13;21(10):e0360626. doi: 10.1371/journal.pone.0360626 (PMC13660919; doi:10.1371/journal.pone.0360626)
Supplement: Supplementary file 17 [file pone.0360626.s017.pdf]

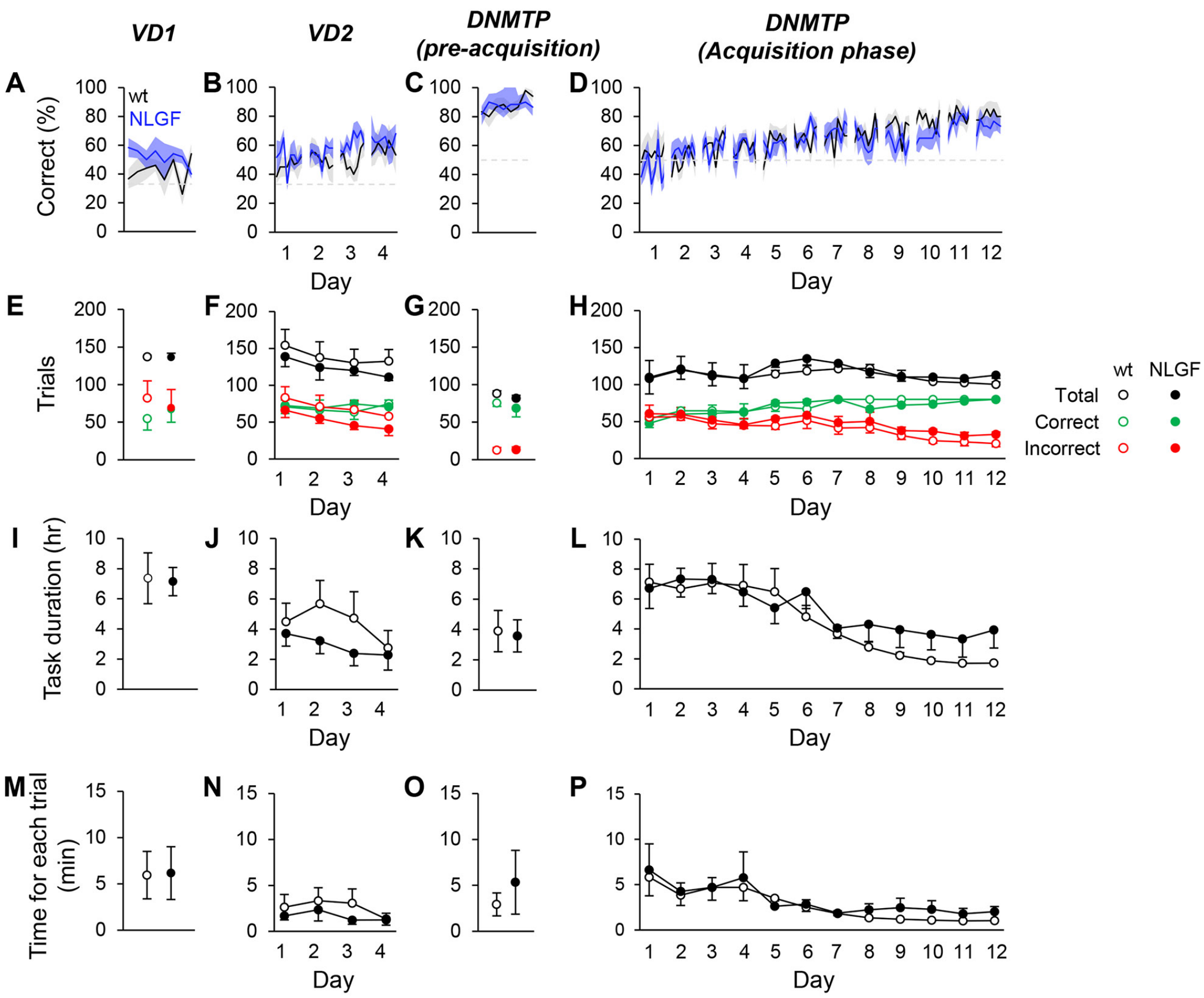

S17 Fig. Additional results of the DNMTP test in female *App*$^{NL-G-F}$ mice.

(**A-D**) Correct rates for each 10-trial bin across tasks. Results are shown for the first 8 bins recorded each day. (**E-H**) Total number of trials (black), correct trials (green), and incorrect trials (red) for each day. (**I-L**) Session duration for each day. (**M-P**) Average time elapsed per trial.
