# Supplementary material for: Operant House: A versatile open-source platform for automated operant conditioning testing of mice in home cages
Source: PLoS One. 2026 Oct 13;21(10):e0360626. doi: 10.1371/journal.pone.0360626 (PMC13660919; doi:10.1371/journal.pone.0360626)
Supplement: Supplementary file 18 [file pone.0360626.s018.pdf]

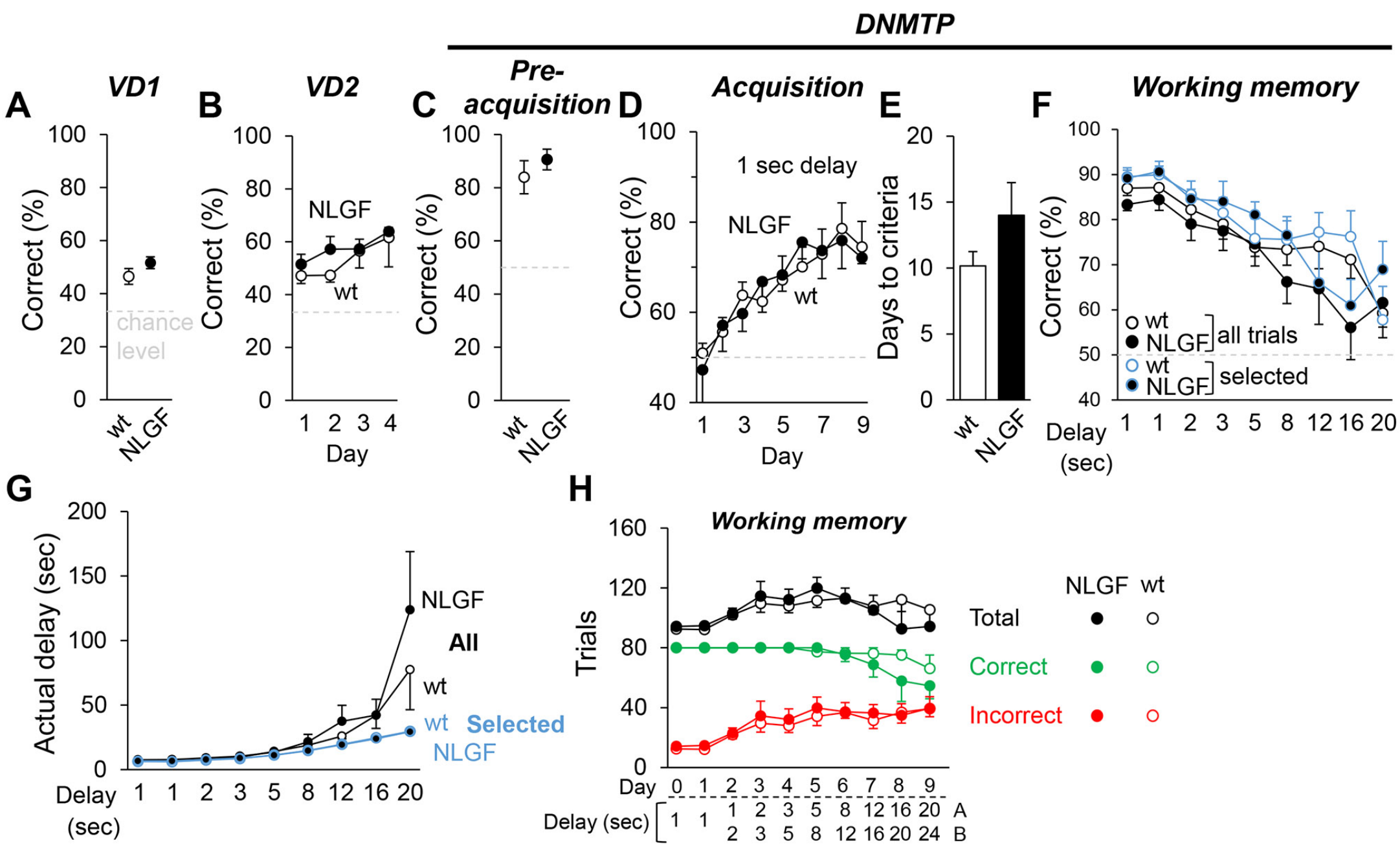

S18 Fig. Male App^{NL-G-F} mice exhibited normal working memory in the DNMTP test at 5 months of age.

(**A, B**) Results of VD1 and VD2 tasks in male homozygous App^{NL-G-F} mice at 5 months of age. (**C-F**) Result of the DNMTP test, including the pre-acquisition (**C**), acquisition (**D,E**), and working memory (**F**) phases. No significant differences were observed between genotypes. (**G**) Actual delay times in trials meeting the delay criterion were comparable between wild-type and App^{NL-G-F} mice. (**H**) Total numbers of each trial type across all trials. n = 6 for controls and n = 5 for homozygous mice.
