# Supplementary material for: Operant House: A versatile open-source platform for automated operant conditioning testing of mice in home cages
Source: PLoS One. 2026 Oct 13;21(10):e0360626. doi: 10.1371/journal.pone.0360626 (PMC13660919; doi:10.1371/journal.pone.0360626)
Supplement: Supplementary file 19 [file pone.0360626.s019.pdf]

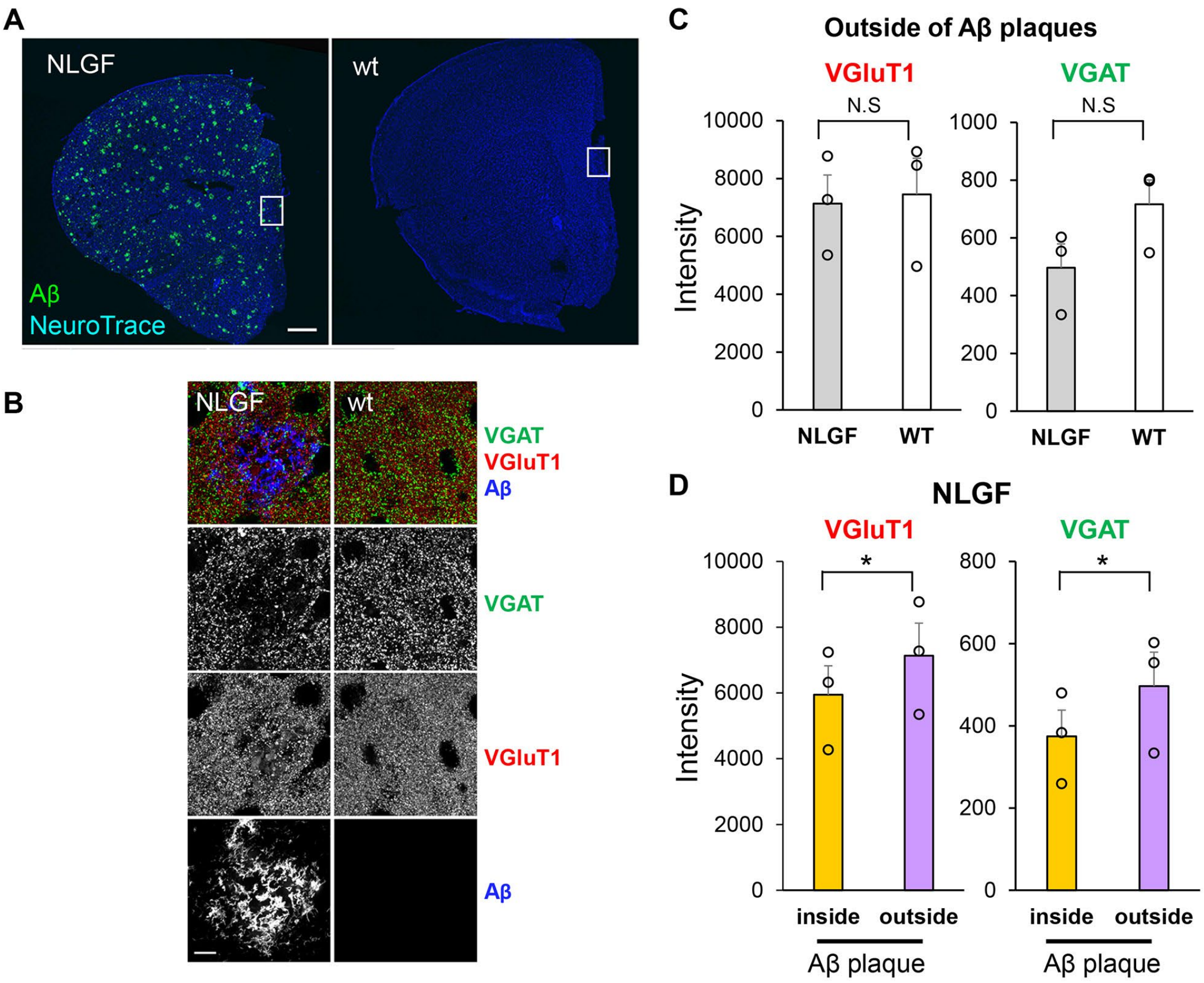

S19 Fig. Reduced presynaptic marker immunoreactivity within Aβ plaques in the mPFC of *App*$^{NL\text{-}G\text{-}F}$ mice at 5 months of age.

(**A**) Representative images showing amyloid β (Aβ) plaque accumulation in the mPFC of 5-month-old male *App*$^{NL\text{-}G\text{-}F}$ (NLGF) mice and their wild-type (wt) littermates. Neurons were stained with NeuroTrace. Scale bar, 500 µm. (**B**) Enlarged images of the regions indicated by squares in (A), immunostained for presynaptic markers VGAT (green), VGluT1 (red), and Aβ (blue). Scale bar, 10 µm. (**C**) Quantification of average fluorescence intensities for VGluT1 (left) and VGAT (right) in regions outside Aβ plaques (defined as clusters of Aβ immunoreactivity > 50 µm^2). For statistical analysis, measurements from 7–8 sections were averaged within each mouse, and mouse-level averages were compared between genotypes using Welch's *t*-test. Neither VGluT1 nor VGAT differed significantly between genotypes, although VGAT showed a tendency toward lower intensity in NLGF mice (Welch's *t*-test; VGluT1, $p = 0.8521$, Hedges' $g = -0.13$; VGAT, $p = 0.1357$, Hedges' $g = -1.21$). (**D**) Average fluorescence intensities for VGluT1 (left) and VGAT (right) inside (red) and outside (black) Aβ plaques in the mPFC of NLGF mice. For each section, the inside–outside difference was calculated, and these differences were averaged within each mouse before paired statistical comparison. Both VGluT1 and VGAT intensities were significantly lower inside than outside Aβ plaques (paired *t*-test; VGluT1, $p = 0.0211$, Hedges' *g*

= −2.21; VGAT, p = 0.0476, Hedges' g = −1.44). n = 3 mice per genotype for (**C**) and n = 3 NLGF mice for (**D**); 7–8 sections were analyzed per mouse. *$p < 0.05$.
