# Supplementary material for: Operant House: A versatile open-source platform for automated operant conditioning testing of mice in home cages
Source: PLoS One. 2026 Oct 13;21(10):e0360626. doi: 10.1371/journal.pone.0360626 (PMC13660919; doi:10.1371/journal.pone.0360626)
Supplement: Supplementary file 20 [file pone.0360626.s020.pdf]

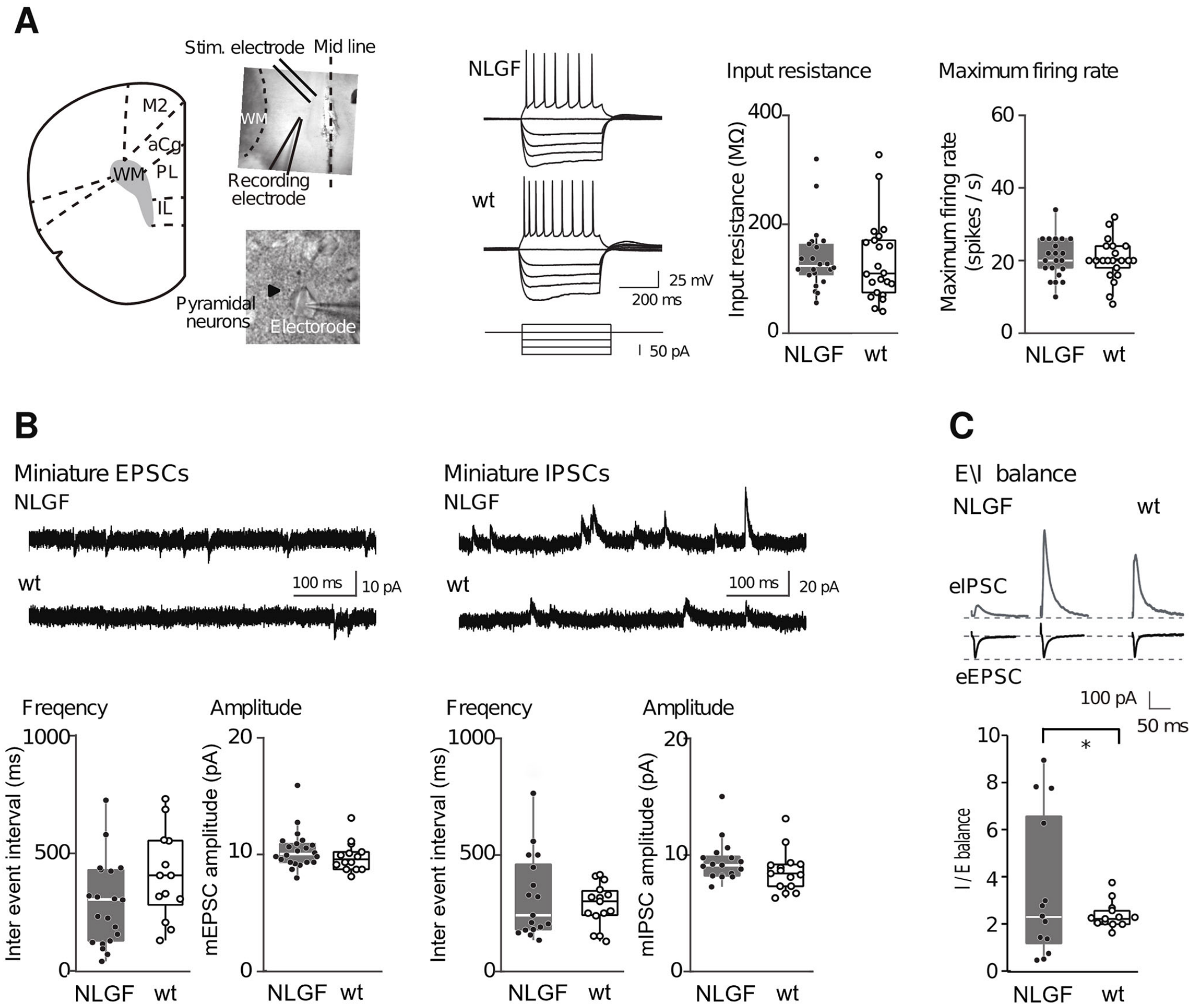

S20 Fig. Increased variability in excitatory–inhibitory balance in *App*$^{NL\text{-}G\text{-}F}$ mice at 5 months of age.

(**A**) Whole-cell patch-clamp recordings were performed from pyramidal neurons in acute mPFC slices prepared from 5-month-old male *App*$^{NL\text{-}G\text{-}F}$ (NLGF) and wild-type (wt) mice (left). Representative regular firing patterns evoked by current injections are shown (middle). Individual-cell measurements and their mean ± SEM for input resistance and maximum firing rates are shown (right). (**B**) Frequency and amplitude of miniature EPSCs (mEPSCs) and miniature IPSCs (mIPSCs) in pyramidal neurons. Representative traces are shown (top). Individual-cell measurements and their mean ± SEM for mEPSC and mIPSC inter-event intervals and amplitudes are shown. (**C**) Ratio of evoked IPSC amplitude to EPSC amplitude (I/E ratio), with stimulus intensity adjusted to evoke ~100 pA EPSCs. I/E ratios for individual cells are shown, together with mean ± SEM. Increased cell-to-cell variability in the I/E ratio was observed in *App*$^{NL\text{-}G\text{-}F}$ mice using a heteroscedastic linear mixed-effects model that accounted for clustering of multiple cells within individual mice (natural-log-transformed I/E ratios; likelihood-ratio test,

$\chi^2(1) = 23.31$, $p < 0.0001$; n = 13 cells from 3 mice per genotype [wild-type: 6, 4, and 3 cells per mouse; *App*$^{NL\text{-}G\text{-}F}$: 3, 5, and 5 cells per mouse]).
