# Supplementary material for: Operant House: A versatile open-source platform for automated operant conditioning testing of mice in home cages
Source: PLoS One. 2026 Oct 13;21(10):e0360626. doi: 10.1371/journal.pone.0360626 (PMC13660919; doi:10.1371/journal.pone.0360626)
Supplement: Supplementary file 21 [file pone.0360626.s021.pdf]

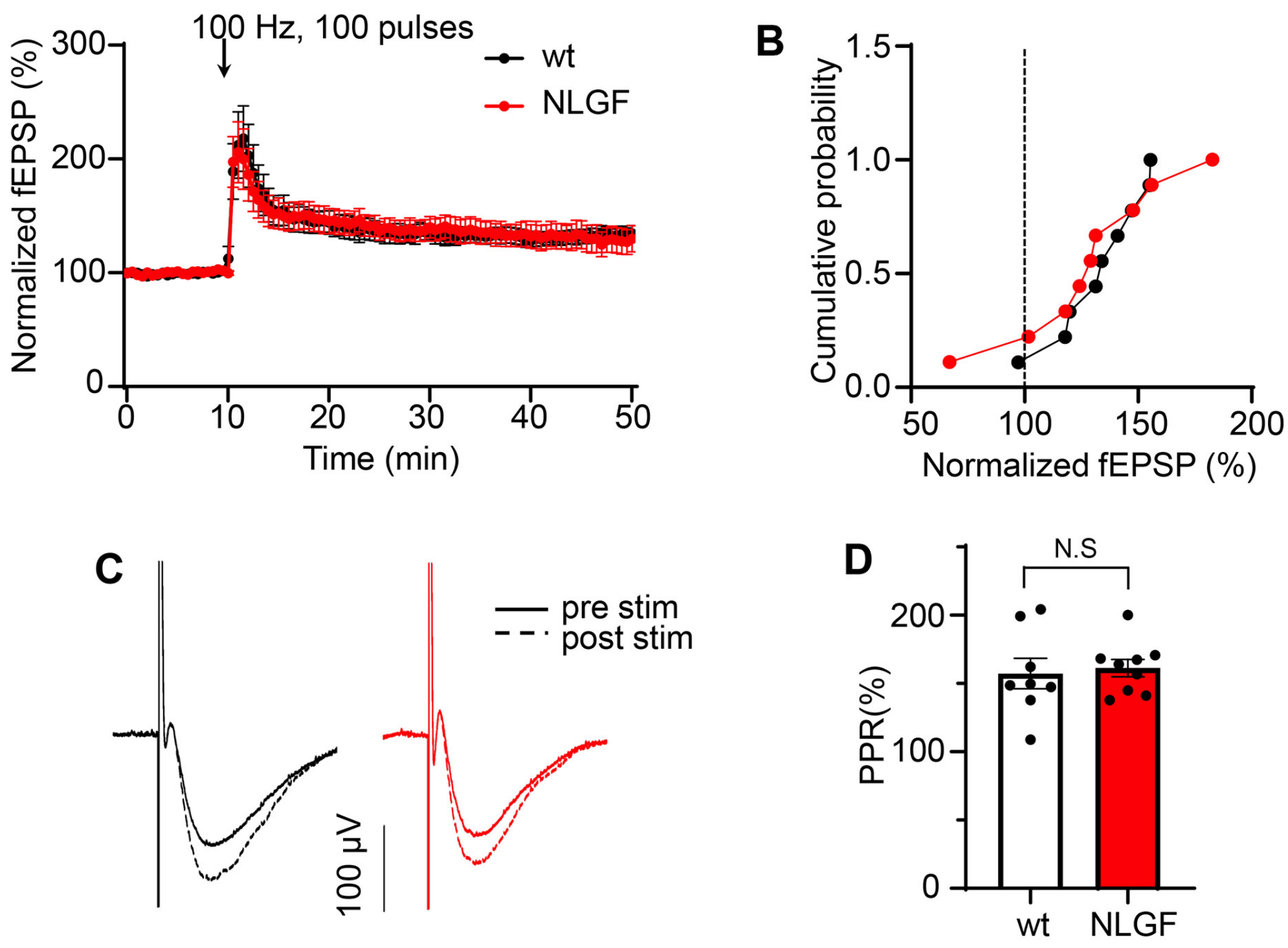

S21 Fig. No detectable alteration in hippocampal long-term potentiation (LTP) in 5-month-old *App*$^{NL\text{-}G\text{-}F}$ mice.

(**A**) Representative LTP traces recorded in the CA1 region following tetanic stimulation (100 pulses at 100 Hz) of Schaffer collaterals. (**B**) LTP magnitude measured after induction. Individual-slice measurements and their mean ± SEM are shown. LTP magnitude did not differ significantly between genotypes using a heteroscedastic linear mixed-effects model that accounted for clustering of multiple slice recordings within individual mice ($F_{1,4} = 0.059$, $p = 0.821$). (**C**) Representative fEPSP traces recorded before and after LTP induction. (**D**) Paired-pulse ratios (PPRs) of fEPSPs after LTP induction. Individual-slice measurements and their mean ± SEM are shown. PPR was calculated as the ratio of the amplitude of the second fEPSP to that of the first, using an inter-stimulus interval of 50 ms. PPR did not differ significantly between genotypes using a heteroscedastic linear mixed-effects model that accounted for clustering of multiple slice recordings within individual mice ($F_{1,4} = 0.289$, $p = 0.620$). For both analyses, genotype was included as a fixed effect and mouse identity as a random intercept. $n = 9$ slice recordings from 3 mice per genotype (3 slices from each mouse).
