# Supplementary material for: Operant House: A versatile open-source platform for automated operant conditioning testing of mice in home cages
Source: PLoS One. 2026 Oct 13;21(10):e0360626. doi: 10.1371/journal.pone.0360626 (PMC13660919; doi:10.1371/journal.pone.0360626)
Supplement: Supplementary file 22 [file pone.0360626.s024.pdf]

## Point-by-point responses to Reviewers' comments

We thank the reviewers for their time and constructive feedback, and we are encouraged by their indication that our manuscript will be acceptable once the remaining issues are addressed. In the revised version, we have also replaced two of the main figures and expanded the number of Supporting Information figures from 7 to 21 to provide more detailed schematics and additional analyses. Below, we provide detailed, point-by-point responses to each comment. Newly added text in the revised manuscript is shown in blue in the marked-up copy.

**Reviewer #1**:

"*The authors of this manuscript developed the "Operant House," a novel, low-cost, and programmable device designed to improve operant conditioning experiments in mice. The device contains a touchscreen, retractable levers and a water reward system, making it suitable for automated behavioral testing in home cages. In addition, the authors validate the system using two working memory assessments, the delayed non-match-to-position test and the two-choice spatial discrimination test by comparing behavior performance between wildtype mouse and APPNL-G-F Knock in mouse. The findings underscore the Operant House's potential benefits for studying cognitive functions in preclinical research. However, I recommended as rejection for the following reasons.*

*The primary concern is the manuscript attempts to cover two distinct themes within a single paper, the development of the Operant House system and its application in Alzheimer's disease research. However, the extensive scope results in a lack of necessary detail and references for both aspects. The technical development of the Operant House would benefit from additional methodological details, comparisons with existing systems, and citations supporting its design choices and advantages.*"

We thank the reviewer for this thoughtful assessment. We agree that the primary purpose of this manuscript is to describe the Operant House, a custom-built, low-cost, and programmable device designed for automated operant conditioning tests in individually housed mice within their home cages. Commercial operant conditioning systems are generally expensive, and large-scale experiments involving many mice require substantial time and financial resources. In contrast, existing do-it-yourself systems often lack flexibility in the range of operant tasks they can support. The Operant House can be readily constructed using 3D-printed parts and supports a variety of operant tasks using either a touchscreen or levers, while enabling parallel testing of many mice in their own home cages. We aim to make this device broadly available to the research community so that it can be widely adopted for assessing cognitive function in animals using operant tasks.

We also acknowledge that our effort to illustrate the utility of the Operant House by including experiments in an Alzheimer's disease model may have blurred the manuscript's primary focus. Following the reviewer's suggestion, in the revised manuscript we have moved findings unrelated to the behavioral experiments (e.g., electrophysiology and immunohistochemistry in the AD model) to the Supplementary Information. In addition, we have substantially expanded the technical description of the Operant House in the main text, figures, and Supplement. The revision now includes:

- A complete bill of materials for all 3D-printed and electronic components, and step-by-step assembly procedures.
- A detailed specification of chamber dimensions, including the overall cage size and the positions and dimensions of the apertures on the touchscreen mask. We emphasize that these dimensions are identical to those described in Clelland et al., *Science*, 2009, in which the two-choice spatial discrimination (TCSD) task was performed in mice, thereby ensuring that compatible operant tasks can be executed in a fully automated, home-cage environment.

"*For instance, there is commercial touch screen system shows similarity compared to Operant House (https://lafayettelifesciences.com/listing/mice-touch-chambers-components), it also provides several protocol for various purpose, which was published or reviewed in many articles, such as "Daniel Palmer, Julie R. Dumont, Tyler D. Dexter, Marco A.M. Prado, Elizabeth Finger, Timothy J. Bussey, Lisa M. Saksida, Touchscreen cognitive testing: Cross-species translation and co-clinical trials in neurodegenerative and neuropsychiatric disease, Neurobiology of Learning and Memory.*"

We thank the reviewer for pointing out commercially available touchscreen systems and related literature. As the reviewer notes, many commercial and DIY operant conditioning chambers (operant boxes) already exist. However, these systems are generally not designed for home-cage analysis: animals are housed in their standard cages and are transferred to an operant box only for scheduled sessions (typically 30–120 minutes). After each session, the animals are returned to their home cages, and this process is repeated daily throughout the behavioral paradigm. In contrast, the Operant House integrates housing and operant testing within the same chamber, allowing subjects to remain in place continuously (24 hours/day, 7 days/week) until the behavioral paradigm is completed. To make this decisive difference explicit, we have cited the reference suggested by the reviewer and stressed the major difference in the Discussion and have also added a schematic illustrating the concept of "home-cage analysis" in the revised manuscript.

"*It is crucial to establish the validity and reliability of Operant House before applying it to hypothesis-driven experiments, such as those involving APP-NL-G-F knock-in mice. To ensure methodological rigor, a more structured validation process should be conducted, including comprehensive benchmarking against established methods, replication studies, and thorough assessments of reproducibility*."

We appreciate the emphasis on rigorous validation. In this study, our DNMTP and TCSD tasks closely followed protocols established for non–home-cage operant boxes (Kwak *et al.*, *Exp. Neurobiol.*, 2015; Clelland *et al.*, *Science*, 2009). Using the Operant House, we reproduced the key outcomes reported in these

prior studies for both tasks. Notably, the Operant House yielded much faster acquisition than typical non–home-cage systems. For example, in TCSD, acclimation/shaping/pre-training that usually require ~26 days were completed in only ~3 days in our setup. As the reviewer suggests, we now explicitly discuss these differences from the earlier studies in the Discussion of the revised manuscript. To support reliability and reproducibility, we have also expanded the technical documentation (main text and Supplement), including a complete bill of materials and assembly steps, so that other laboratories can independently build and evaluate the system. Comprehensive, multi-site benchmarking is an important next step; here, our goal was to establish validity by replicating well-characterized tasks and outcomes while documenting the system in sufficient detail to enable independent replication.

"*For example, the authors could first validate the system using wild-type animals alone, while also increasing the sample size per sex and age group to assess potential sex- and age-related effects on task performance.*"

Following the reviewer's suggestion, we validated the DNMTP task in wild-type male and female mice. Statistical analyses detected no significant sex differences at any stage of the paradigm, indicating that sex did not affect task performance in wild-type animals. We did not examine age effects in the present study; this is an important direction that we plan to address in future experiments. In the revised manuscript, we have added the female and male wild-type results to Figure 7 and Supplementary Figure 18, respectively.

"*Meanwhile, the application to Alzheimer's disease research lacks sufficient introduction of prior studies, mechanistic interpretations, and broader implications for cognitive assessments in disease models.* "

As noted above, the primary goal of this manuscript is to introduce the Operant House and to demonstrate its performance. We used the Alzheimer's disease (AD) model only to illustrate utility. In line with the reviewer's advice, we have added a concise background on AD and AD mouse models relevant to our behavioral assays. To maintain focus, results not directly tied to the behavioral experiments (e.g., electrophysiology and immunohistochemistry in the AD model) have been moved to the Supplementary Information.

"*The difference between APP-NL-G-F Knock in mouse and wild type mouse in synaptic activity is really interesting, however, the linker between this to the other part of paper is almost none. To improve clarity and depth, I recommend splitting the manuscript into two separate papers, ensuring that both the technical and experimental aspects receive the necessary depth of analysis.*"

Data from the APP-NL-G-F model are presented solely to validate the Operant House by testing tasks—DNMTP and TCSD—that are known to engage prefrontal functions and are relevant to working-memory deficits in AD models. We have revised the main text to make this positioning explicit and have relocated several datasets to the Supplement to avoid confusing the manuscript's focus. We believe this streamlined

structure clarifies the relationship between the technical platform and its disease-model demonstration, while preserving the manuscript's primary emphasis on the Operant House itself.

**Reviewer #2:**

"*The authors' team proposed the work titled "A Versatile Open-Source Platform for Automated Operant Conditioning Testing of Mice in Home Cages". There are some specific comments in the following before a possible publication.*

*1. What is its edge or motivation of the proposed method? What is the remaining challenge?*"

We appreciate the request for a clearer statement of the platform's motivation and advantages. While we outlined these points in the original Introduction, we have now expanded and clarified them and added a schematic comparing workflows in the revised manuscript.

**Motivation and edge:**

Operant conditioning is a well-established and powerful approach for assessing cognitive function in mice; however, conventional operant-box workflows are labor-intensive, time-consuming, and costly. In typical setups, mice are maintained in standard home cages and transferred to an operant box only for scheduled sessions (usually 30–120 min). For cohorts of 10–20 animals, running 3–4 sessions per day over several weeks demands substantial expert labor. In contrast, the Operant House integrates the operant interface into the home cage, making human intervention minimal and enabling continuous 24/7 testing with multiple units in parallel at low per-unit cost. Although home-cage–based operant systems have been reported, they are often limited in the range of operant paradigms they can implement and/or are too expensive to deploy at scale. Operant House addresses both constraints: it is low-cost and open-source (all design files, 3D-print parts, electronics, and source code are provided) and supports a broad repertoire of tasks.

In our hands, Operant House also accelerates acquisition relative to non–home-cage systems. For example, in TCSD, the acclimation/shaping/pre-training phases that typically require ~26 days were completed in ~3 days with Operant House. We have emphasized these points and provided a schematic to clearly illustrate the workflow and time/cost advantages in the revision.

**Remaining challenges**:

1. Turnkey availability. Many users prefer off-the-shelf systems rather than self-assembly. While we provide the full bill of materials, STL files (model data for 3D printing), wiring diagrams, and firmware/software openly, broader dissemination would benefit from a low-cost manufacturing/distribution pipeline (e.g., partner vendors or institutional core facilities) to deliver assembled units.
2. Task repertoire and analytics. Operant House currently implements 11 operant tasks by default, covering most standard paradigms. Going forward, we aim to expand the library and leverage the on-board video stream for AI-based behavioral analysis, enabling richer, higher-throughput cognitive readouts.

We have incorporated these clarifications and plans into the Introduction and Discussion of the revised manuscript.

"*2. More advanced methods using deep learning and AI in detection and recognition are suggested for discussion, e.g., SpectralGPT (DOI:10.1109/TPAMI.2024.3362475), Cross-city matters (doi.org/10.1016/j.rse.2023.113856)*"

We appreciate the suggestion to discuss advanced AI methods (e.g., SpectralGPT, Cross-city matters). While our long-term goal is indeed to assess cognition under task-free, freely moving conditions, operant learning remains indispensable at present because of its extensive validation in the literature. The Operant House is therefore valuable as a platform that greatly streamlines operant testing.

Importantly, Operant House continuously records behavior during tasks, which makes it straightforward to combine with deep-learning–based analytics to enrich cognitive readouts (as the reviewer suggests). In the revised manuscript, we now discuss how video streams from Operant House could be analyzed using modern DL approaches (including foundation-model–style pipelines and cross-domain transfer), and how such integration could further improve the fidelity and throughput of cognitive assessment.

"3. *Please clarify the contributions, why this proposed method is important? What is the main difference from existing ones*."

Several systems have been developed to enable automated operant conditioning in or near the home cage, but each has important limitations. As outlined in the original Introduction, the CombiCage (Remmelink et al., *Sci Rep* 2017), which connects a home cage to a commercial operant chamber dedicated to the 5-choice serial reaction time task, is relatively large and expensive, making simultaneous testing of multiple mice logistically and financially challenging. IntelliCage (Kiryk et al., *Behav Brain Res* 2020) allows operant-like testing in group-housed mice with individual RFID tags in a single large cage and is useful for assessing group-level behavior, but the presence of cage mates can influence individual performance. Thus, there is a need for cost-effective and efficient systems that enable 24/7, minimal-intervention experiments and parallel scaling of automated operant conditioning in individually housed mice within their home cages.

Below, we position Operant House among existing low-cost, home-cage–based systems:

- ArduiPod Box (Pineño, *Behav Res* 2014): A home-cage–mountable system using an iPod Touch and Arduino. By default, it implements a simple operant task in which the animal touches the screen according to the color of the panel to receive a water reward.
- Psibox (Francis & Kanold, *Front Neural Circuits* 2017): A home-cage–mountable system using a computer, a National Instruments data acquisition (DAQ) board, and a custom printed circuit board. Since its interface is restricted to an auditory speaker and a water-bottle spout, the system supports only a simple operant task in which the animal must lick the water-bottle spout in response to an auditory tone.
- ROBucket (Devarakonda et al., *Behav Res* 2016) and DIY-NAMIC (Lee et al., *eNeuro* 2020): These systems use dedicated cages equipped with Arduino-based hardware to support more complex operant paradigms. However, their input interfaces are fixed: the number (≤3) and spatial

arrangement of nose-poke ports cannot be changed, touchscreen and lever-press tasks cannot be implemented, and they lacks illumination for delivering punishment cues and do not support a dedicated behavior camera for direct video monitoring.

- ARTS (Poddar et al., *PLOS ONE* 2013): A rack-based system in which a single computer and National Instruments DAQ board control operant tasks in multiple cages. It supports unique, complex joystick-based tasks, it does not accommodate touchscreen or lever-press paradigms, substantially lack its compatibility with the majority of tasks implemented in conventional operant devices.

By contrast, one major advantage of Operant House is that it supports computer-automated touchscreen batteries for higher-order cognitive testing that have been developed over the past two decades for human, non-human primate and rodent models. Operant House is structurally modular: by changing only the LCD monitor program and mask pattern, a wide variety of visually guided operant tasks can be implemented. By swapping the chamber walls, conventional lever-press tasks can also be performed easily, and even in its default configuration, the system includes 11 standard operant tasks. In addition, an integrated IR camera and house light are built into the unit, enabling quantitative analysis of posture dynamics and behavioral patterns (e.g., grooming, rearing, scratching) using deep-learning–based tools such as DeepLabCut.
In the revised manuscript, we have added a concise description of these points to the Discussion to clearly distinguish Operant House from existing systems and to clarify its specific contributions and importance.

"*4. More ablation analysis should be given.*"

Following the reviewer's suggestion, we performed a prefrontal cortex lesion experiment using ibotenic acid in wild-type animals and confirmed a marked impairment on the TCSD task. These new results are included as Figure 9 in the revised manuscript.

"*5. How about the computational complexity or running time?*"

Operant House requires modest computational resources. Nose-poke detection is implemented by defining regions of interest and thresholding pixel-intensity changes within those regions. The program runs at 30 fps, enabling 30 fps video recording concurrently. Even when all software functions are active (simultaneous video acquisition and nose-poke detection), remote desktop access (e.g., TeamViewer, RealVNC, Chrome Remote Desktop) remains responsive. We have added these implementation and performance details to the revised manuscript.

**Reviewer #3**:
"*The paper is very well written and detailed. The methods and results are properly presented, including figures. However, I think that the content of the paper is not suited to be published as it is, for the following reasons.*

We appreciate the reviewer's careful reading and constructive suggestions. Below we address each point.

*1) Based on the presentation, it seems that the initial study had very different aims, mainly experimental, while it is now presented in a form of a validation of a open hardware / open source behavioral platform. The authors present experiments that are not even related to behavioral results, and are far beyond the declared scope of the manuscript. For this reason, I believe that the authors should remove immunostaining and electrophysiology analyses from the manuscript, and focuses only on the validation of the behavioral setup. Adding these is confounding and cannot support the efficacy/flexibility of the device (any device is compatible with such ex vivo investigations).*"

We apologize for the confusion caused by the original presentation. The primary aim of this manuscript is to introduce the Operant House, which enables fully automated operant tasks within individually housed home cages. The AD model data were included solely to demonstrate utility, not to extend the scope beyond behavioral validation. Following the reviewer's advice, in the revised manuscript we have moved the immunostaining and electrophysiology results to Supplementary Information and expanded the description of the device and its behavioral validation in the main text.

"*2) Behavioral data are clear and rigorous. However, since these data are presented for validating the device, these should be discussed deeply in relation to literature based on different devices.*"

We agree. The Discussion now explicitly compares our DNMTP and TCSD results obtained with Operant House to prior literature using non–home-cage operant boxes, highlighting agreements and differences (e.g., acquisition times, training schedules, and performance metrics).

"*In addition, more information should be provided on the protocol results (I mean on the functioning of the device and its measurements, not limited to final behavioral parameters). Since the focus of the manuscript is on technical description and validation of the device, these data should not be supplementary.*"

We have added a new section detailing device functioning and measurements (e.g., task state logic, event timing/latencies, trial structure, readout definitions) and incorporated these into the main text with a new figure (Figure 4) rather than leaving them in the Supplement.

"*3) Description of the device should not be in the methods section but in the results. In addition, I believe that more details on the assembly and a table with parts costs and providers are warranted. I visited the web site and it is very complete (although a bit "old style"), I really appreciate the work done, but I believe that the paper should present part of those information in the main text as well.*"

We thank reviewers for visiting our website and for the positive feedback. In the revised manuscript, the device description has been moved to the Results, and we have added assembly details, a bill of materials with costs and vendors, and related schematics in the main text and Supplementary Figures S1–S12.

"*4) The device is presented as "all integrated into an individually housed home cage", and seems to represent a final solution to the problem of performing behavioral tasks in home cages. This also emerges*

*from the introductory discussion of several other solutions available in the literature. However, it is clearly not true: the device is just a custom operant chamber, separated from house cages, so that the mouse must be transferred there for behavioral experiments (thus leading to the whole series of stress-related effects that are well known). This aspect should be clarified, and only the real positive aspects of the device should be stated.*"

We regret the misunderstanding—this is not the case. Operant House is integrated into the individually housed home cage; mice are placed in the Operant House at the start of the paradigm and remain there continuously (often for weeks) until completion, eliminating the repeated transfers between a housing cage and a separate operant box. This design reduces experimenter workload and prevents transfer-related stress. To avoid further confusion, we have added a conceptual schematic (Figure 1) and improved the textual explanation of the home-cage automation principle.

"*Related to this point, is pointless to mention the compatibility of the system with techniques (such as miniscope) without explaining why another similar device should not be compatible, or how the system can facilitate such integration.*"

We now describe how Operant House facilitates miniscope integration (e.g., dedicated mounting points, unobstructed headstage clearance, and cable/tether routing). We have also provided step-by-step instructions and 3D-printed adapter model files for UCLA Miniscope v3/v4 (cable preparation, slip-ring modification, and computer settings) on our website and reference this in the revised text: https://operanthouse.sakura.ne.jp/office1001099.html. Additionally, we expanded the comparison with previously reported home-cage operant systems to clarify practical integration differences.

"*5) as for the LTP experiments (that however are pointless here), the authors should provide statistical tests*"

We have now included the Kolmogorov–Smirnov test for the LTP datasets and added the corresponding statistics to the figure legend in the Supplement.
