# Supplementary material for: Operant House: A versatile open-source platform for automated operant conditioning testing of mice in home cages
Source: PLoS One. 2026 Oct 13;21(10):e0360626. doi: 10.1371/journal.pone.0360626 (PMC13660919; doi:10.1371/journal.pone.0360626)
Supplement: Supplementary file 23 [file pone.0360626.s025.pdf]

Point-by-point responses to Reviewers' comments

We thank the reviewers for their time and constructive feedback. We are encouraged by their positive assessment of our manuscript and by the indication that it will be suitable for publication once the remaining issues are addressed. Below, we provide detailed, point-by-point responses to each comment. Newly added text in the revised manuscript is shown in blue in the marked-up copy.

Reviewer #1:

"*This is an excellent manuscript describing "Operant House," a flexible and low-cost platform for automated operant conditioning in individually housed mice.*
I appreciate the versatility, the clear documentation, and the fact that several different cognitive tasks can be conducted without handling or transferring animals.
This approach is likely to reduce stress, increase throughput, and broaden accessibility for labs without specialized behavioral facilities.

The manuscript is written clearly, with extensive methodological detail suitable for replication. Parts lists and the tailored website will help to reproduce the system.
Statistical analyses are appropriate, the validation experiments are convincing, and the proof-of-concept AD model data (now in the supplementary information) is presented in a balanced way. It is also evident that the manuscript has significantly benefited from previous review rounds.

I did not identify any major flaws or substantive weaknesses. A few very minor typographical or formatting inconsistencies remain, but these do not detract from the science. Overall, this work represents a valuable contribution to the field of automated behavioral testing and will be of interest to groups studying cognition, neuropsychiatric disease models, and high-throughput behavioral pipelines.

In my opinion, the manuscript is suitable for publication in its current form, and I encourage acceptance."

We greatly appreciate these positive and encouraging comments.

Minor inconsistencies:

"*Typo in the abstract: "within in their home cages-duplicated `in'*."
"*Acronym: PL/IL vs PI/IL not consistent.*"

We have corrected these typographical and formatting inconsistencies in the revised manuscript.

Reviewer #2:

"The manuscript describes a new behavioral setup which combines housing and an operant learning chamber using LCD screens (giving CS), nose poke through a wall with openings (CR), water delivery (US, reward) and LED lights (US, punishment). The authors shows that different behavior protocols can be performed in this setup. I think the work is interesting and this could be a ground work for interesting future development, but the manuscript needs some additional work to optimize the dissemination of the tool and ideas."

"I wrote my comments before seeing that it had already be reviewed (I modified it slightly afterwards). I got to similar conclusions that earlier reviewers: both the setup description and the AD-related experiments (so good to see acceptable control group in a AD experiment!) are interesting but should be in two different papers. This will also make the review much easier, and more importantly for the authors and the community: it would make the AD experiments much more visible."

"I disagree with the comment to put the setup description in the material and methods, but agree with most other comments..."

We thank Reviewer #2 for the positive assessment of our study and for appreciating not only the Operant House system itself but also the analyses using the Alzheimer's disease model. We agree that the Alzheimer's disease-related results are of interest in their own right. However, we would respectfully prefer to retain these data in the present manuscript, because we believe that inclusion of the behavioral analyses in the Alzheimer's disease model is important for demonstrating the utility of the Operant House in a biologically and medically relevant context.

In the previous round of revision, one reviewer also commented that the manuscript was somewhat difficult to follow because it combined the development and design of the Operant House with the analyses of the Alzheimer's disease model. In response to that concern, in the revised manuscript we moved sections not directly related to the development and design of the Operant House, such as the electrophysiological and immunohistochemical analyses in the Alzheimer's disease model, to the Supplemental Information. At the same time, we substantially expanded the descriptions related to the development and design of the Operant House in the main text, figures, and supplementary materials. We hope that these revisions have made the main focus of the manuscript clearer and improved its overall readability.

We believe that retaining the behavioral analyses of the Alzheimer's disease model strengthens the manuscript by providing a concrete demonstration of the usefulness of the Operant House. For this reason, we feel that presenting both the methodological advance and its application within a single manuscript provides a stronger and more informative account of the work.

Because Reviewer #1 did not raise the concern that the manuscript should be divided into two separate papers, we would respectfully leave the final decision on this point to the Editor.

"…I would also not ask for additional experiments, but in absence of a direct comparison of this setup against a previously published one, with experiments done in parallel, the discussion should be a bit more convincing."

We thank the reviewer for this helpful suggestion. In response, we have added a new section to the revised main text addressing differences in performance between the Operant House and conventional operant chambers. Specifically, for the lever-press task, we have added a description of how the results shown in Fig S13 compare with those reported in previous studies, in order to better clarify the characteristics and performance of the present system.

"An additional comment compared to earlier review that is essential: the ethical aspects of the setup are critical. In the setup, animal are housed in isolation, with food in the bedding for weeks, this will not be possible in Europe and the authors need to discuss both the stressful effect on their experiments, and ways to change the protocol.
NB : Directive 2010/63/EU (Council of the Europe Union 2010): "Animals, except those which are naturally solitary, shall be socially housed in stable groups of compatible individuals. In cases where single housing is allowed in accordance with article 33(3) the duration shall be limited to the minimum period necessary and visual, auditory, olfactory and/or tactile contact shall be maintained." This can be kept in the discussion for now, but protocol adjustment to minimise single housing should be a objective for future work."

We thank the reviewer for raising this essential point. We agree that single housing is an important welfare issue and should be scientifically justified, limited to the minimum duration necessary, and refined where possible. In the present study, mice were singly housed to allow individual behavioral testing without interference from cage mates. We acknowledge that prolonged single housing may affect stress and behavior, and we have added this as a limitation and welfare consideration in the revised Discussion. We also state that minimizing the duration of single housing and preserving sensory contact where feasible should be important goals for future refinement of the Operant House protocol.

"The authors need to convince me a bit more that the "Operant House not only reproduces behavioral performance obtained in conventional non–home-cage apparatuses but also enhances experimental efficiency by shortening the overall duration of behavioral paradigms." I would like a bit more focus on that aspect (while minimizing single housing). This would be needed to really convince people to use the setup. They only discuss two other papers doing similar experiments without mentioning mice genetic background or age. This would need a more systematic description and comparison.

Following Reviewer #2's suggestion, we have expanded the revised manuscript to include a more systematic comparison with four previous studies using conventional non-home-cage operant conditioning apparatuses. Where available, we now include information such as sex, age, and genetic background in order to make the comparison with the present study clearer and more informative.

“The second part of the paper about the AppNL-G-F mice should be moved to another paper, I think. I encourage the authors to make two companion papers: one about the new protocol and setup, one about the AD related experiment. I was pleased to see AD experiments where the control group makes sense, thank you for that. I did not review the non-behavioral part of the manuscript as it is out of my expertise, and I believe it deserves its own paper.”

As discussed above, although we greatly appreciate Reviewer #2’s positive assessment of both the Operant House system itself and the analyses using the Alzheimer’s disease model, we would respectfully prefer to retain these data in the present manuscript.

“Major point: Open source and open data aspects
Please indicate license of the software and hardware if you want to call it "open source."

We thank the reviewer for this important comment. The Operant House will be released under the MIT License, and we have now clearly indicated this in the main source code repository and on the Operant House website.

“Instruction and data should be provided in a long-term archived version; websites tend to disappear. Please use a data repository to host the information for the long term (zenodo for example). I would also encourage the author to move the description into a community friendly tool with version control, like Codeberg or Gitlab, or a similar tool, so people can give feedback there. (This should be very easy as the current documentation on the website looks like a markdown-based wiki).”

We thank the reviewer for this helpful advice. Because we have been using GitHub for development and version control, we have now moved the Operant House documentation site from a private server to GitHub Pages, which we believe provides a more stable, transparent, and community-friendly platform for long-term access and user feedback.

“As helper for open hardware (please only use if you think it can help you, not because I mention it), you may have a look at the OSHWA certification program for open hardware (https://certification.oshwa.org/) and the hardware documentation guide I put together (https://open-make.github.io/Hardware-template-guide/).”

We thank the reviewer for introducing us to OSHWA and for sharing these helpful resources. We believe that the philosophy of the OSHWA certification program, particularly its support for researcher-led open-hardware development in academia, closely aligns with the goals of the Operant House project. Accordingly, we have submitted the Operant House project for OSHWA certification.

“You mentioned data is available, but it is not. Please give access to all csv files and the analysis code (you can use zenodo for that too).”

In response, we have now made the relevant CSV files, analysis code, and associated videos publicly available in a GitHub repository, and we have added the corresponding link to the revised manuscript. The repository URL is as follows:
https://github.com/Shintaro18/DataForOperantHousePaper_PlosOne

Minor points:

“The first part of the papers sounds too much like an advertisement, as if Fig.1 A. In particular, you should mention the disadvantage of a home cage setup early on and discuss it (abstract and introduction): single housing, no involvement of the human who could detect stress,...”

We thank Reviewer #2 for this important comment. We understand the concern that the early part of the manuscript may have overemphasized the advantages of the Operant House without sufficiently acknowledging the limitations and practical considerations associated with home-cage behavioral testing. In response, we have revised the Abstract, Introduction, and Discussion to provide a more balanced description of the system.

Specifically, we now state earlier in the manuscript that, although home-cage operant testing offers advantages such as reduced handling and improved experimental efficiency, it also requires careful consideration of animal welfare, particularly with respect to individual housing and the potential effects of social isolation. We have also clarified that the Operant House enables live monitoring of the entire chamber and retrospective inspection of recorded task videos, which facilitate assessment of the animals’ health status and possible signs of stress during experiments. In addition, we have expanded the Discussion to more explicitly address these limitations and the importance of further refinement to minimize the burden associated with single housing.

“The electrophysiology and immunostaining experiments are not mentioned in the abstract, title or introduction. Authors should either rewrite those or take out these experiments out of the paper and keep it for another paper more related to the biology experiments (see main comment).”

In response, we have added a description of the electrophysiological and immunohistochemical analyses to the Abstract.

“Please put the material and method section before the result section.”

Following the reviewer’s suggestion, we have moved the Materials and Methods section to precede the Results section.

“Indicate the condition of housing first, indicate when light was turned on/off and indicate the number of animals per cage, and size of the cage before the experiment started.”

The light on/off schedule is described in the final part of the section entitled “Automated Operant Conditioning Using the Operant House.” We have added the other requested information to the Materials and Methods section of the revised manuscript, including the housing conditions prior to the experiment, the number of animals per cage, and cage size.

“Explain better what light conditions and water restriction were used during the operant house. Make a better figure of the habituation time that includes access to water and link it in the text, I could not follow: do the mice have no water during the first 12h?

Please explain protocols without referring to other protocols. You may tell differences after the initial description, but this is more for the result/discussion section than the material and method.”

In response, we have added a clearer description of the habituation procedure, light conditions, and water restriction schedule to the Materials and Methods section of the revised manuscript. We have also added the water restriction schedule to Figs. 5A and 8A.

“I do not understand ‘the stimulus water supply was not provided’”.

We thank the reviewer for pointing this out. “Stimulus water supply” referred to the “stimulus reward” shown in Figs. 5B and 8B. Because this terminology was inconsistent in the original manuscript, we have now unified it throughout the revised manuscript as “stimulus reward.”

“Add a table for version 1 and version 2 differences in protocols.”

In response, we have included in Fig. S3 of the revised manuscript a table summarizing the differences in the protocols between Operant House Version 1 and Version 2.

"To prevent the mice from acclimating to a specific pattern of changes, two delay schedules, A and B, were randomly assigned to each trial." Please explain what schedules is it, and what do you mean with "randomly assigned": each mouse got a different series, did you use a randomisation table, was randomisation computer generated?”

We thank the reviewer for pointing out that this description was unclear. In the working memory task, two different delay durations were used within a given session; for example, on day 4 of the working memory phase, the delay was either 5 s or 8 s on each trial. The sequence of these delays was determined by a computer-generated randomization table based on a specified seed value. To clarify this procedure, we have added a concrete example of the delay pattern used on day 4 of the working memory phase to the right side of Fig. 5F.

Because the Operant House allows the seed value to be specified, the same delay sequence can be reproduced exactly when the same seed is used. In the present experiments, all mice were tested using the same seed value on each day and therefore experienced identical delay patterns. The seed values used were as follows: day 1, 1; day 2, 2; day 3, 3; day 4, 4; day 5, 5; day 6, 6; day 7, 7; and day 8, 8. We have also added an explanation of this point to the Materials and Methods section of the revised manuscript.

"Please indicate the number of animals and number of cohorts for the experiments."

The number of animals is indicated in the figure legends, and we have also clarified in the revised manuscript that all experiments were conducted in a single cohort.

"Results. Explain why did you choose midnight as the start of the operant time, this is not when the animals are most active?"

Because mice are nocturnal and show high levels of activity during the dark phase, we initiated the task at midnight, which corresponds to 3 h after the onset of the dark phase, in order to ensure that all animals were reliably awake when the session began.

"Give a better image of the masking wall and refer to the image in the text, also indicate the material used for the wall."

In response, we have added to Fig. S11 both an overall photograph of the masking wall and a magnified image showing its surface texture more clearly. We have also added a description of the masking wall, including the material used, in the main text.

"accuracy 100% (comparison between automated detection and human counting during the first 30 min of the VD2 session)" please give us data, was it counted by 2 humans? If not, please rephrase."

We thank the reviewer for this helpful comment. We apologize that this description was unclear. The comparison was based on manual counting by a single observer, not by two independent observers. In this analysis, the number of nose pokes detected automatically matched the number counted manually during the first 30 min of the VD2 session. To avoid overstating this validation, we have revised the wording in the manuscript accordingly. The corresponding video data have also been made publicly available in our GitHub repository.

"Implemented operant tasks" this list is longer than what is presented in the paper. Maybe add these other tasks in this paper if you split the manuscript."

As discussed above, we would prefer to retain the Alzheimer's disease model data in the present manuscript. While space limitations do not allow us to present experimental data for all operant tasks that have already

been implemented, we believe that listing these successfully implemented tasks is nevertheless useful for illustrating the versatility of the Operant House.

"'Water intake: 25-35 µl per lick.' how was this calculated?"

The estimated water intake per lick was calculated by dividing the decrease in water-bottle weight measured before and after the task session by the total number of licks recorded during the session. Because the bottle tip contains two metal balls that keep the outlet closed except during licking, we consider the contribution of evaporation to be negligible.

"longer actual delay time: it is not clear why this was specified in the AD experiments and not before. This should be part of the description of the behavior and not come at an extra element with the experiment with transgenic animals. "we selected trials whose actual delay time was within 1.3 times the specified delay time plus 10 seconds": this is part of the material and method section, explain why that selection, and when did you make it."

We thank the reviewer for this important comment. As pointed out, control of the actual delay duration was also necessary in the DNMTP experiment using ibotenic acid-treated mice, as in the other DNMTP experiments. In response, we have added a new panel showing the actual delay duration (Fig. 6P), and we have added the performance calculated using only trials that met the actual-delay criterion to Fig. 6O (blue outlines).

During this reanalysis, we also found an error in data processing. Although the Materials and Methods section stated that performance was analyzed using trials assigned to delay A, the previous graph had inadvertently been generated using the average of delay A and delay B. We have now corrected this and replaced the graph with the values obtained from delay A only (Fig. 6O, black outlines).

Following the reviewer's suggestion, we have also added an explanation of the trial-selection procedure based on actual delay duration to the end of the DNMTP section in the Materials and Methods. The criterion of programmed delay × 1.3 + 10 s was adopted empirically because trials exceeding this threshold often reflected poor task engagement by the animals. This criterion was defined after we noticed, during the $App^{NL\text{-}G\text{-}F}$ mouse experiments, that the actual delay duration was longer in the mutant group than in the control group; however, we did not compare multiple alternative thresholds.

"Although not statistically significant (genotype: F1,10 = 1.117, p = 0.315), AppNL-G-F mice tended to have longer actual delay times compared to wild-type mice (Fig 7G)": if this is not significant, there is no reason to mention it."

Although the mean actual delay duration in the $App^{NL\text{-}G\text{-}F}$ group was nearly threefold longer than that in the wild-type group, the difference was not statistically significant. In accordance with the reviewer's suggestion, we have removed this statement from the revised manuscript.

"Discussion:

Please discuss the results, not putative expansion of the system you do not have or show data of (even when this is asked by other reviewers :)).

"quantitative analysis of posture dynamics": either show you can do it, or do not mention it. I would guess that a camera on top is not sufficient to do that."

In response, we have added new data demonstrating that the positions of the nose and hindquarters can be tracked using DeepLabCut (Fig. S21). On this basis, we retained the corresponding statement in the Discussion.

"Additionally, the software includes a TTL signal output and timestamp functionality, enabling seamless integration with external devices for experiments involving optogenetics.": please indicate time precision in ms if you want to discuss this."

We thank the reviewer for this helpful comment. We agree that timing precision should be stated explicitly if TTL output is discussed. Because this value was not measured directly for the complete Operant House system, we have revised the manuscript to clarify that the cited precision is based on previously reported performance of the underlying platform, rather than on direct validation of the full setup in the present study.

"Please compare habituation time in hours too (in your setup 1 day is 24 hours, what is in Clelland et al ?"

Unfortunately, a comparison of habituation time in hours was not possible because Clelland et al. did not report the relevant procedural details. That study stated only that mice were pre-trained through several iterative stages, referring to Morton et al. (Nat. Methods, 2006), which likewise did not provide a detailed description of the duration of the habituation or pre-training sessions.

"Statistics:

Please give more information on statistics:

The representation and analysis of the data suppose the data is normally distributed, while it probably is not, at least it cannot be for 9A and 9B (as you may have mice never reaching the criteria). Please use boxplots or jitter points, and other tests (unless you can prove the data is normally distributed). Indicate how scores are calculated and give sample sizes (number of mice, number of trials): it should be one score per mice (not one score per trial), but this is not explained."

In Fig. 9, all mice reached the criterion and proceeded to the TCSD task. Because the sample sizes were small, we did not perform formal tests for normality. In response to the reviewer's suggestion, we therefore changed the statistical analyses for two-group comparisons from the t-test to the Mann–Whitney U test, and from the paired t-test to the Wilcoxon signed-rank test. For the two-way repeated-measures ANOVA, we

retained this analysis because it was the most suitable method for assessing the effects of genotype and session in these repeated-measures data.

We have also clarified in the Materials and Methods section ("Data analysis and statistics") that the scores used for the operant tasks were calculated on a per-session basis, not on a per-trial basis. Thus, each mouse contributed one score per day. The number of mice is indicated in the figure legends, and the numbers of trials are provided in Figs. 7H, 9F, 10G, S14F–J, S15E–H, S17E–H, and S18H.

Regarding data presentation, we added plots of individual data points to the quantitative graphs for the immunohistochemical analyses in Fig. S19. For the operant-task graphs, we retained the current format, as boxplots or jitter plots would become visually crowded given the number of groups and sessions and might reduce clarity. Because the underlying data are publicly available in our GitHub repository, readers interested in the detailed distribution of the data may access them there.

"You also often test the data multiple times without mentioning having a bonferroni correction. Be a bit more straight on your tests: for each experiment have one statistical test that matters first, and for any other test, you may not mention any statistics. Selecting part of the data for the analysis a posteriori is a p-hacking strategy."

We thank the reviewer for this important comment. In the DNMTP and TCSD tasks, Bonferroni correction was applied when repeated comparisons were performed, and this is stated in both the figure legends and the Materials and Methods section.

Regarding the concern about multiple testing, our *a priori* hypothesis was specifically that $App^{NL\text{-}G\text{-}F}$ mice would show impaired working memory relative to wild-type mice. Accordingly, the key analysis was focused on the working memory phase, whereas the earlier phases primarily served as shaping steps to habituate the mice to the apparatus and train them in the task rules. We therefore do not think it would be appropriate to combine all phases into a single statistical framework, as this would test a different and much broader hypothesis than the one addressed in this study. In addition, because the acquisition phase differed in duration across animals, it was not technically feasible to include all phases from VD1 through the working memory phase in a single repeated-measures ANOVA. We have clarified this rationale in the revised manuscript.

Additional change not directly requested by the reviewers

In Fig. S16, we found that, for one of the mice used in the DNMTP task, the touch-detection sensitivity for panel responses in Operant House version 2 had been lowered during the course of the experiment. We therefore replaced this figure with the corresponding dataset excluding this mouse. This change does not affect the results, interpretation, or conclusions of the manuscript.
