# Supplementary material for: Operant House: A versatile open-source platform for automated operant conditioning testing of mice in home cages
Source: PLoS One. 2026 Oct 13;21(10):e0360626. doi: 10.1371/journal.pone.0360626 (PMC13660919; doi:10.1371/journal.pone.0360626)
Supplement: Supplementary file 24 [file pone.0360626.s026.docx]

**Point-by-point responses to reviewers’ comments**

We thank the reviewers for their time and constructive feedback, and we are encouraged that the remaining issues are relatively minor and can be addressed in the present revision. Below, we provide detailed, point-by-point responses to each comment. Newly added text in the revised manuscript is shown in blue in the marked-up copy.

**Reviewer #2:**

**1. “***The manuscript has been only slightly changed. Please note I did not perform a review of the non-behavioral aspects of this paper. I leave it to the editor to state whether this part was reviewed enough from the other reviewers, as authors do not want to take them out.*

*Please add a section about data and code availability with correct links, and provide an archive of them (via zenodo or GIN). Please also add these links in the website. I could also not find the MIT licence, please make it more visible.*

*A lot of “response to the review” did not seem to have made their way into the manuscript, I am puzzled whether this is the latest version. In general, the response given are satisfactory, but I do not see the changes in the manuscript.*”

We thank the reviewer for these comments. We apologize that the extent of the revisions made in response to the previous review was not sufficiently apparent. The revisions were indicated in blue in the marked-up version but were not visible in the clean version, and the distinction between the two files may not have been sufficiently clear. To avoid any further confusion, we have carefully rechecked the current manuscript against every item in our previous point-by-point response and have confirmed that all of the revisions described there are included in the manuscript.

As stated in the previous version of the manuscript, the Python code (OperantHouse1.04.py) was publicly available at https://shintaro18.github.io/office1001.html, together with the files required for 3D printing and the complete parts list. The header of the Python code file, OperantHouse1.04.py, already contained the full MIT License notice. However, we agree that the licensing information was not sufficiently prominent on the website itself. To make it immediately visible, we have now added the statement “Software available on this website is released under the MIT License” to the copyright section of the website and have included a direct link to the MIT License.

In response to the reviewer’s request in the previous review round, the data underlying the study had also been uploaded to the same website. Upon rechecking the website, we found that some files were not accessible as intended because of a technical issue. We sincerely apologize for this problem. We have corrected the issue, tested all download links, and confirmed that the data and associated files are now accessible.

In addition, in accordance with the reviewer’s present request, we have deposited a permanent, versioned archive containing the original raw output files, processed datasets underlying the figures and statistical analyses, Python source code, 3D-printing files, parts list, and associated documentation in Zenodo ([doi.org/10.5281/zenodo.22019760](https://doi.org/10.5281/zenodo.21811532)). The permanent archive link has also been added to the project website.

Finally, we have added a dedicated “Data and Code Availability” section to the revised manuscript. This section provides direct links to both the project website and the permanent Zenodo archive and clearly specifies the locations of the underlying data, source code, 3D-printing files, parts list, and licensing information.

**2.** ”*One exception where I want to insist: in the discussion, the authors should be focusing on discussing the results and not bragging about what else is possible with the hardware. They may mention other paradigms briefly, but it should not take more than two sentences (it is a whole paragraph).*”

We thank the reviewer for this comment. We apologize if the paragraph gave the impression that we intended to promote or overstate the capabilities of the hardware. This was not our intention. The paragraph was originally included to clarify the broader applicability of the system and its distinction from existing approaches in response to comments raised during the earlier review process.

Nevertheless, we agree that the Discussion should focus primarily on the findings of the present study. Accordingly, we have substantially shortened the relevant paragraph in the Discussion to two sentences, as suggested by the reviewer. We have also revised the Results and Discussion to clearly distinguish among tasks that were experimentally demonstrated in the present study, tasks that have been evaluated in a separate study, and other paradigms that have been implemented and technically tested but have not yet undergone systematic behavioral validation in animals.

**3.** ”*Please also look at each answer and see why the manuscript does not contain answers you mention (for example discussion of performance compared to other 4 studies: still find only 2, only age of animals was added: please also discuss this: is it the same, is it better, why ? This is not the only example, please revisit).*”

We thank the reviewer for pointing this out. We have carefully re-examined the entire manuscript together with our previous point-by-point response to confirm that each change described in the response is clearly reflected in the manuscript.

Regarding the comparison with four previous studies, all four studies were included in the previously revised manuscript, although we recognize that the relevant comparisons were distributed between the Results and Discussion and may therefore not have been sufficiently easy to identify. Specifically, in the Discussion, we compared performance in the working-memory task conducted in Operant House with that reported for a conventional non-home-cage touchscreen-based delayed non-matching-to-position (DNMTP) task by Kwak et al. (2015). We also compared our results with those obtained using a conventional touchscreen-based location-discrimination task by Clelland et al. (2009), including the available information on the age and sex of the animals. In addition, we discussed the results of a location-discrimination (LD) task performed in a conventional non-home-cage touchscreen apparatus by Saifullah et al. (2020) in relation to performance in the corresponding task in Operant House. Finally, in the Results, we compared acquisition of the lever-press task in Operant House with that reported using a conventional non-home-cage lever-press apparatus by Yu et al. (2009). In accordance with the reviewer’s previous suggestion, we included the age, sex, and genetic background of the animals wherever this information was available. However, we agree that listing these experimental characteristics alone does not make sufficiently clear whether the behavioral performance was comparable to, or more efficient than, that reported previously. We have therefore revised the relevant sections to state more explicitly, for each comparison, whether the final behavioral performance was comparable, whether task acquisition or completion was faster, and which methodological differences limit direct quantitative comparison.

In brief, the revised text now clarifies that Operant House produced behavioral performance broadly comparable to that reported using conventional non-home-cage apparatuses, while shortening task acquisition or preparatory training in some paradigms. At the same time, we have avoided claiming superiority where differences in task design, training criteria, mouse strain, age, sex, or reporting methods preclude a rigorous direct comparison.

To make these comparisons easier to locate, we have consolidated and more clearly signposted the relevant text in the revised manuscript and have explicitly described the paradigm, apparatus, available animal characteristics, training duration, and principal performance measures for the relevant comparisons. We have also reviewed all other responses from the previous round and confirmed that the corresponding revisions are present and clearly identifiable in the marked-up manuscript.

**4.** “*The data provided is not the raw data and the claim from the authors that new graphs can be made out of may be inaccurate (I do not have time to look into details here). Please provide raw data as you get it out of the hardware, this is important for future users (so they know what to expect) and describe the analysis, that I supposed was made in excel? This can be made in the github repository. I would also look again at the statistics when I get access to the data*.”

We thank the reviewer for this important comment. In the previous revision, we made available the processed numerical data used to generate the graphs in the manuscript. These files allow the figures to be reproduced and alternative graphs to be generated from the processed values, but we agree that they are not the original files directly produced by the Operant House hardware. We apologize that this distinction was not sufficiently clear.

In response to the reviewer’s request, we have now uploaded the original raw output files generated directly by Operant House for all experiments reported in the manuscript, except for the video files, to both the GitHub repository (https://github.com/Shintaro18/DataForOperantHousePaper_PlosOne) and the permanent Zenodo archive ([doi.org/10.5281/zenodo.22019760](https://doi.org/10.5281/zenodo.21811532)). These raw files include, for example, the original Touch*.txt and Touch*.csv files generated during behavioral testing.

The Operant House software has been updated several times during the course of this study, and the format of the raw output files consequently differs among software versions. Different file formats therefore require the corresponding analysis program or Excel spreadsheet. To make the relationship between the raw data and the appropriate analysis method clear, we have organized the repository by data format and placed the corresponding analysis program or spreadsheet in the same folder as each set of raw data. We have also added a README file that specifies, for each experiment:

1. the format and contents of the raw output files;
2. the corresponding analysis program or spreadsheet;
3. the manuscript figure and panel to which each dataset corresponds.

For datasets analyzed using the dedicated Windows programs, users can run the corresponding executable file and drag and drop the relevant raw data file, such as a Touch*.txt or Touch*.csv file, into the program. The program filenames and the README clearly indicate which raw-data format is supported by each program. Where Excel was used for subsequent calculations or graph preparation, we have provided the corresponding spreadsheets, including the formulas used to derive the plotted values. We have also expanded the “Data Analysis and statistics” subsection of the revised manuscript to describe this workflow.

The video files generated during behavioral testing were not included in the GitHub repository because each file is typically approximately 300 MB and the complete set is very large. These videos were used for routine monitoring and were not used to derive the quantitative outcomes reported in the manuscript. To show future users the format and quality of the video output, we have provided a representative video file in the permanent archive ([doi.org/10.5281/zenodo.22019760](https://doi.org/10.5281/zenodo.21811532)).

Finally, we have rechecked the processed datasets against the raw output files and re-examined all statistical analyses. Where appropriate, the statistical methods were revised to account explicitly for repeated measurements, data distribution, and multiple comparisons, as detailed in our responses to Reviewer #3, and effect-size estimates have been added where applicable. The numerical source data underlying each figure, together with the files used for statistical analysis, are now included in the archive. We have also added clearer descriptions of the statistical procedures to the Methods and have provided the software and settings used for each analysis. We hope that these additions will allow the reviewer and future users to inspect the original hardware output and reproduce the data-processing and statistical-analysis workflow.

**Reviewer #3:**

“*This manuscript presents the development of the Operant House, a low-cost and automated home-cage system for operant conditioning in mice. This is a well-designed study that addresses an important challenge in behavioral neuroscience. The system is affordable, flexible, and has the potential to make advanced behavioral testing available to more laboratories. The manuscript is clearly organized, and the revisions have improved several parts of the paper, especially the description of animal welfare and experimental methods. The work is suitable for publication after a few minor issues are addressed.*”

**1.** “*The statistical analyses appear appropriate, but some important details are still missing. The authors should report the sample size for every experiment more consistently in the Results or figure legends. It would also help to explain how normality was assessed before using parametric tests, why outliers were removed with the Smirnov-Grubbs test, and to include effect sizes where possible. These additions would make the statistical analysis more complete.*”

We thank the reviewer for these helpful suggestions. We have carefully rechecked all experiments and revised the Methods and figure legends to report the number of animals and, where applicable, the number of sections, cells, or recordings contributing to each analysis. For the principal behavioral analyses, the individual animal was treated as the experimental unit, and repeated observations within animals were analyzed using mixed-effects models.

We comprehensively re-examined the statistical analyses to better account for the distribution and repeated-measures structure of the data. Correct/incorrect responses in the operant tasks are now analyzed using generalized linear mixed-effects models (GLMMs) with a binomial error distribution and logit link function, with genotype or treatment condition and session (or other repeated-measure factors, as appropriate) as fixed effects and mouse identity as a random effect. *Post hoc* comparisons are adjusted using the Holm method, and odds ratios (ORs) are reported as effect-size estimates. Simulation-based model diagnostics were performed to assess residual uniformity, dispersion, and potential outliers.

For continuous outcomes involving repeated measurements, we now use linear mixed-effects models (LMMs), with group, the repeated-measure factor, and their interaction as fixed effects and mouse identity as a random effect. Model assumptions were evaluated after fitting the models by examining residual distributions, including assessment of residual normality using the Shapiro–Wilk test. Where necessary, data were transformed to satisfy model assumptions. *Post hoc* comparisons were adjusted using the Holm method, and Hedges’ *g* is reported as an effect-size estimate for pairwise comparisons where applicable.

We also reconsidered our previous use of the Smirnov–Grubbs test for outlier exclusion. In the revised mixed-effects analyses, no observations were excluded solely on the basis of statistical outlier testing; potential outliers were instead evaluated as part of the model-diagnostic procedure. The revised “Data analysis and statistics” subsection now describes these procedures explicitly.

Finally, we clarified the unit of analysis for the supportive histological and electrophysiological experiments in Figs. S19–S21. For Fig. S19, section-level measurements were averaged within each mouse, and the mouse was treated as the experimental unit. For the electrophysiological analyses in Figs. S20 and S21, individual recorded neurons or slice recordings were treated as observational units, and the numbers of both animals and recordings are explicitly reported in the corresponding figure legends. In Fig. S20, Levene’s test was used specifically to assess cell-to-cell variability, which is interpreted as cellular heterogeneity rather than as an animal-level estimate of genotype variance.

Thus, the revised manuscript now reports sample sizes and units of analysis explicitly, uses statistical models appropriate to the data structure, describes model diagnostics and multiple-comparison procedures, and provides effect-size estimates where applicable.

**2.** “*The authors state that the Operant House can support many behavioral tasks. While this is likely true, only the DNMTP and TCSD tasks are fully validated in this study. The Discussion should make a clearer distinction between tasks that have been experimentally tested and those that are expected to work but have not yet been validated.*”

We thank the reviewer for this important comment. We agree that the previous wording did not distinguish sufficiently clearly between tasks that have been implemented and technically tested in the Operant House software and tasks that have been evaluated experimentally in animals.

All of the tasks listed in the manuscript have been implemented in the source code available on the project website, and we have confirmed that the corresponding programs operate as intended at the software and hardware levels. However, as the reviewer correctly notes, technical implementation does not by itself constitute behavioral validation.

In the present study, the visual discrimination, lever-press, DNMTP, and TCSD tasks were experimentally demonstrated in mice, with the DNMTP and TCSD tasks being used to characterize cognitive phenotypes in *App^NL-G-F^* mice. During the review of this manuscript, a separate study using the Operant House to perform a trial-unique nonmatching-to-location (TUNL) task was also accepted for publication. Thus, the TUNL task has now been evaluated experimentally in a separate study, although it was not validated as part of the present manuscript.

In accordance with the reviewer’s suggestion, we have revised the Discussion to distinguish explicitly among: (1) tasks experimentally examined in the present study; (2) the TUNL task, which has been evaluated in a separate study; and (3) other tasks that have been implemented and technically tested but have not yet undergone systematic behavioral validation in animals. We have also revised statements that could have implied that all available tasks had been behaviorally validated. The revised text now states clearly that the unvalidated tasks represent potential applications of the system that will require task-specific experimental validation.

**3.** “*The different findings between female and male AppNL-G-F mice are interesting. The Discussion would benefit from a little more explanation of why these sex differences may exist and whether they could reflect differences in disease progression or task demands*.”

We thank the reviewer for this thoughtful comment. We agree that the sex-dependent behavioral phenotypes observed in *App^NL-G-F^* mice are interesting and warrant further consideration. In the previous version of the Discussion, we compared our findings with earlier studies reporting sex-dependent differences in the behavioral phenotypes of *App^NL-G-F^* mice and discussed the possibility that the DNMTP and TCSD tasks assess partially distinct cognitive processes.

To address the reviewer’s comment more directly, we have now expanded this discussion to clarify that the observed differences could reflect either sex-dependent trajectories of disease-related circuit dysfunction or differences in the cognitive and behavioral demands of the two tasks. The DNMTP task requires animals to retain and update information across a delay, whereas the TCSD task requires discrimination among spatially distributed visual stimuli and may place different demands on cognitive flexibility and response selection. However, because the present study was not designed to compare neuropathological progression or circuit dysfunction between female and male mice, we cannot distinguish among these possibilities. We have therefore presented these interpretations cautiously and have explicitly identified them as hypotheses requiring further investigation.

**4.** “*The manuscript discusses the limitations of single housing, which is appreciated. Adding a brief description of body weight monitoring, health checks, or other welfare measures during the experiments would further strengthen this section.*”

We thank the reviewer for this helpful suggestion. We agree that the procedures used to monitor animal welfare during prolonged individual housing should be described more clearly.

During the experiments, body weight was measured daily, and the general condition of each animal was assessed by visual inspection. The health checks included assessment of appearance and grooming, posture, spontaneous activity, apparent food and water intake, and any signs of injury or distress. Body weight was used as an important objective indicator for detecting possible deterioration in health. Animals showing substantial weight loss, abnormal behavior, or other signs of poor health would have been promptly evaluated and, when appropriate, removed from the experiment in accordance with the predetermined humane-endpoint criteria. No animals met these exclusion or humane-endpoint criteria during the experiments.

We have added these welfare-monitoring procedures to the Materials and Methods. We have also added a brief statement to the Discussion emphasizing that, when Operant House is used under individually housed conditions, regular body-weight measurements and visual health assessments are important for identifying potential welfare problems at an early stage.

**5.** “*This manuscript presents a useful and well-validated behavioral platform that could be valuable for many neuroscience laboratories. The study is carefully performed, and the main conclusions are supported by the data. The remaining concerns are relatively small and mainly involve improving the reporting of statistical methods, clarifying some methodological choices, and slightly strengthening the Discussion.*”

We sincerely thank the reviewer for this encouraging assessment of our work. We greatly appreciate the reviewer’s careful and constructive evaluation throughout the review process. The comments have helped us improve the clarity of the statistical reporting, methodological descriptions, and Discussion.

**Reviewer #4:**

**1. “***The authors present an automated operant conditioning testing platform, and the experimental procedures are described in a detailed and accessible manner. However, I have a concern regarding how the relevant prior literature is framed.*

*The following two studies appear to be directly relevant to the present work:*

*・PLoS One. 2013 Dec 6;8(12). “A fully automated high-throughput training system for rodents.” PMID: 24349451*

*・Front Neural Circuits. 2017 Mar 1;11:10. “Automated Operant Conditioning in the Mouse Home Cage.” PMID: 28298887*

*Although these papers are cited in the Discussion section, I think they should also be introduced in the Introduction. Since readers generally encounter the rationale and background of a research article through the Introduction, directly relevant prior studies should be presented there to clarify what has already been established, what approaches have previously been developed, and what limitations or gaps remain. This would allow the novelty and significance of the present Operant House platform to be evaluated more clearly.*”

We thank the reviewer for this helpful suggestion. We agree that these two directly relevant studies should be introduced in the Introduction, rather than being discussed only later in the manuscript. Accordingly, we have now cited and described both studies in the revised Introduction. We have also clarified what these earlier automated operant-conditioning systems established, as well as the remaining practical and methodological gaps that motivated the development of Operant House. Specifically, the revised text now places Operant House in the context of previous automated and home-cage-based systems by describing both their important contributions and the practical limitations that may restrict broader adoption or flexibility across behavioral paradigms. We then explain how Operant House was designed to address these remaining needs through its combination of low cost, modular hardware, open-source software, compatibility with multiple behavioral paradigms, and suitability for automated testing in individual home cages. We believe that this revised framing places the contribution of Operant House more clearly in the context of prior work without overstating its novelty.

**Reviewer #5:**

**1.** “*Author has addressed all previous comments in a manner that is in my opinion acceptable. Congratulations on the paper and hope the design can be of use for many other labs.*”

We sincerely thank the reviewer for this encouraging comment and for the constructive suggestions provided during the review process. We are very pleased that the reviewer considers our responses and revisions acceptable, and we share the hope that Operant House will be useful to many laboratories.
