# Supplementary material for: Operant House: A versatile open-source platform for automated operant conditioning testing of mice in home cages
Source: PLoS One. 2026 Oct 13;21(10):e0360626. doi: 10.1371/journal.pone.0360626 (PMC13660919; doi:10.1371/journal.pone.0360626)
Supplement: Supplementary file 25 [file pone.0360626.s027.docx]

**Point-by-point responses to Reviewers’ comments**

We thank the Academic Editor and Reviewers for careful review of our revised manuscript and for identifying the remaining issues. These points correspond to the comments raised by Reviewer #3 and are addressed individually below. We believe that these constructive comments have helped us to improve the clarity of the manuscript and the rigor with which the behavioral and electrophysiological findings are interpreted.

**Reviewer #3:**

**1. TCSD interpretation** “*The authors should be more cautious in describing TCSD deficits as working-memory impairment, since the task also involves behavioral flexibility and response selection.*”

We agree with the Reviewer. We have revised the manuscript throughout to distinguish the interpretation of the DNMTP and TCSD tasks more clearly. The DNMTP task is described as directly assessing working memory, whereas TCSD performance is now described as reflecting multiple cognitive processes, including working-memory-related processes, behavioral flexibility, and response selection.

Accordingly, we have revised the Abstract, Introduction, Results, Discussion, figure titles, and concluding statements where necessary. For example, the Discussion now states:

“The DNMTP task directly assesses working memory, whereas performance in the TCSD task likely reflects multiple cognitive processes, including working memory, behavioral flexibility, and response selection.”

Similarly, the Results now describe the phenotype of male *App^NL-G-F^* mice as “impaired TCSD performance, consistent with deficits in working-memory-related processes and/or behavioral flexibility and response selection,” rather than simply as working-memory impairment. The title of Fig. 10 has also been revised to state that male *App^NL-G-F^* mice exhibit “impaired performance in the TCSD task.”

Finally, the overall conclusion now refers to the detection of “early cognitive deficits” in *App^NL-G-F^* mice, while specifically identifying working-memory impairment as the phenotype detected by the DNMTP task.

**2. Small lesion groups** ” *The lesion experiments have very small sample sizes (n = 3–4 per group). The conclusions about the role of the PFC should therefore be presented more cautiously.*”

We agree that the relatively small group sizes in the lesion experiments limit the strength and precision of the conclusions that can be drawn regarding prefrontal cortex involvement.

We have therefore revised the Discussion to state explicitly:

“Because the lesion experiments involved relatively small groups (n = 3–4 per group), these findings should be regarded as supportive evidence for prefrontal involvement and interpreted cautiously with respect to the magnitude of the lesion effects.”

We have also moderated the corresponding language in the Results. For the TCSD experiment, we now describe the lesion phenotype as “impaired TCSD performance” and conclude that the findings “support a contribution of the prefrontal cortex to TCSD performance,” rather than interpreting the lesion effect as definitive evidence of working-memory impairment.

Similarly, for the DNMTP experiment, the delay-restricted analysis is now described as a sensitivity analysis supporting the primary all-trial analysis, rather than as independently confirming a lesion-induced working-memory deficit.

**3. Electrophysiology** “*Several neurons were recorded from the same animals, which may raise concerns about statistical independence. The authors should clearly report the number of animals and cells per animal and explain how this was handled.*”

We thank the Reviewer for raising this important statistical issue. We agree that multiple cells or slices obtained from the same animal cannot be regarded as fully independent biological replicates.

We therefore reanalyzed all genotype comparisons in the electrophysiological experiments shown in S20 and S21 using heteroscedastic linear mixed-effects models. Individual cells (S20) or slice recordings (S21) were retained as observational units, while mouse identity was included as a random intercept to account explicitly for the clustering of multiple recordings within the same animal. Genotype was included as a fixed effect, and genotype-specific residual variances were allowed using the varIdent variance structure in the nlme package. Thus, the individual recordings contribute information to the analysis, while their non-independence within each animal is explicitly accounted for.

For S20, the numbers of recordings from individual animals are now reported explicitly in the figure legend. The recording numbers were as follows:

- S20A: wild-type, 22 cells from 3 mice (12, 2, and 8 cells per mouse); *App^NL-G-F^*, 22 cells from 3 mice (11, 6, and 5 cells per mouse).
- S20B, mEPSCs: wild-type, 14 cells from 4 mice (3, 3, 3, and 5 cells per mouse); *App^NL-G-F^*, 22 cells from 3 mice (8, 9, and 5 cells per mouse).
- S20B, mIPSCs: wild-type, 15 cells from 4 mice (3, 4, 3, and 5 cells per mouse); *App^NL-G-F^*, 17 cells from 3 mice (2, 9, and 6 cells per mouse).
- S20C, I/E ratio: 13 cells from 3 mice per genotype (wild-type: 6, 4, and 3 cells per mouse; *App^NL-G-F^*: 3, 5, and 5 cells per mouse).

For the previously reported difference in cell-to-cell variability in the I/E ratio, we additionally compared a homoscedastic model with a heteroscedastic model allowing genotype-specific residual variances. Because residuals from the untransformed I/E-ratio data were non-normally distributed, the data were natural-log transformed before the final analysis. The estimated residual cell-to-cell variability in the I/E ratio was greater in *App^NL-G-F^* mice, and the heteroscedastic model provided a significantly better fit than the homoscedastic model (likelihood-ratio test, χ²(1) = 23.31, *p* < 0.0001).

We also reanalyzed mIPSC frequency using the mixed-effects framework. After transformation required to satisfy the residual-normality assumption, the previously observed genotype difference in variability was no longer significant. We therefore removed the previous claim of increased mIPSC-frequency variability from the revised manuscript.

For S21, LTP magnitude and paired-pulse ratio were also reanalyzed using heteroscedastic linear mixed-effects models with mouse identity included as a random intercept. For each genotype, 9 slice recordings were obtained from 3 mice, with 3 slices recorded from each mouse. Neither LTP magnitude (*F*_1, 4_ = 0.059, *p* = 0.821) nor paired-pulse ratio (*F*_1, 4_ = 0.289, *p* = 0.620) differed significantly between genotypes.

The Methods, Results, and S20/S21 figure legends have been revised accordingly. We have also clarified that individual-cell and individual-slice measurements are retained as observational units, whereas genotype-level statistical inference is performed using mixed-effects models that explicitly account for clustering of multiple recordings within individual animals.

**4. Delay-time selection** “*Selecting trials based on the actual delay achieved may introduce bias. The authors should report how many trials were excluded and discuss this limitation.*”

We agree with the Reviewer that restricting analyses according to actual delay duration may introduce selection bias because actual delay duration itself can be influenced by the animal’s behavioral state.

We have therefore revised the manuscript in three ways.

First, we now explicitly distinguish the primary analyses, which include all trials, from the delay-restricted analyses, which are treated as sensitivity analyses. The same delay criterion was applied to all experimental groups and was based solely on actual delay duration, irrespective of whether the trial response was correct or incorrect.

Second, we now report the exact numbers and proportions of retained and excluded trials for each delay-restricted analysis:

- **DNMTP lesion experiment (Fig. 6)**: 1,231 of 1,783 saline trials (69.0%) and 1,309 of 2,013 IBO trials (65.0%) were retained; 552 (31.0%) and 704 (35.0%) trials, respectively, were excluded.
- **DNMTP *App^NL-G-F^* experiment (Fig. 7)**: 2,756 of 3,403 wild-type trials (81.0%) and 2,909 of 3,700 *App^NL-G-F^* trials (78.6%) were retained; 647 (19.0%) and 791 (21.4%) trials, respectively, were excluded.
- **TCSD lesion experiment (Fig. 9)**: 445 of 1,514 saline trials (29.4%) and 210 of 1,398 IBO trials (15.0%) were retained; 1,069 (70.6%) and 1,188 (85.0%) trials, respectively, were excluded.
- **TCSD *App^NL-G-F^* experiment (Fig. 10)**: 3,728 of 5,019 wild-type trials (74.3%) and 2,696 of 4,531 *App^NL-G-F^* trials (59.5%) were retained; 1,291 (25.7%) and 1,835 (40.5%) trials, respectively, were excluded.

Third, we have added an explicit limitation to the Discussion. We note that selection based on actual delay duration could retain different subsets of trials across experimental groups, particularly in the TCSD experiments, in which the proportions of retained trials differed substantially between groups. We therefore interpret the delay-restricted analyses only as sensitivity analyses used to determine whether the principal findings persisted when variability in actual delay duration was reduced; they are not treated as independent confirmatory analyses. The primary conclusions are based on analyses including all trials.

**5. Water restriction** “*Since motivation can affect operant performance, body-weight and water-intake data should be reported or discussed more clearly.*”

We agree that motivational state is an important consideration in water-reinforced operant tasks.

We have therefore clarified the water-access protocol and animal monitoring procedures in the Methods. For the DNMTP and TCSD paradigms, cumulative water-access duration was monitored during each daily session. If water-access duration was below the predefined level (DNMTP: 160 s; TCSD: 300 s), supplemental water access was provided immediately after the session. We also clarify that the programmed variable was water-access duration, and that the actual volume of water consumed by each mouse was not directly measured.

Body weight was measured daily as part of routine welfare monitoring during behavioral testing, together with visual assessment of general appearance, grooming, posture, spontaneous activity, apparent food and water intake, and signs of injury or distress.

In addition, we recovered paired body-weight measurements from six wild-type mice tested in independent Operant House version 2 experiments using the same programmed DNMTP water-access regimen (up to 80 water-access periods of 2 s each; maximum programmed access duration, 160 s/day). Mean body weight was 22.47 ± 0.52 g before testing and 21.89 ± 1.11 g after testing, corresponding to no significant group-level change (paired *t*-test, *t*(5) = −0.65, *p* = 0.545). These data have now been added to the Results.

We have also added a limitation to the Discussion. Specifically, because task performance was reinforced by access to water, we acknowledge that differences in motivational state related to thirst cannot be completely excluded. Moreover, because actual water consumption was not directly measured and the available paired body-weight data were obtained from independent experiments rather than from all principal experimental cohorts, individual or genotype-dependent differences in hydration or motivation remain possible.

**Reviewer #4:** “All comments have been addressed.”

We thank Reviewer #4 for the careful evaluation of our revised manuscript and are grateful that the Reviewer considers the previous concerns to have been adequately addressed. No additional changes were required in response to this comment.
