# Supplementary material for: Operant House: A versatile open-source platform for automated operant conditioning testing of mice in home cages
Source: PLoS One. 2026 Oct 13;21(10):e0360626. doi: 10.1371/journal.pone.0360626 (PMC13660919; doi:10.1371/journal.pone.0360626)
Supplement: Peer review history

# Author comment: Author response to Decision Letter 0

**DOI:** 10.1371/journal.pone.0360626.r001

11 Dec 2025

Attachment Submitted filename: Response to reviewer_final.pdf

# Peer review: Decision Letter 0

**DOI:** 10.1371/journal.pone.0360626.r002

**Authors:** Mario Treviño Villegas

18 Jan 2026

Dear Dr. Yuzaki,

Thank you for submitting your manuscript to PLOS ONE. After careful consideration, we feel that it has merit but does not fully meet PLOS ONE’s publication criteria as it currently stands. Therefore, we invite you to submit a revised version of the manuscript that addresses the points raised during the review process.

==============================

Two reviewers have evaluated your work and expressed enthusiasm for it. However, one has raised major concerns that require careful consideration.

Please address all comments point by point in your response.

==============================

Please submit your revised manuscript by Mar 04 2026 11:59PM. If you will need more time than this to complete your revisions, please reply to this message or contact the journal office at plosone@plos.org. When you're ready to submit your revision, log on to https://www.editorialmanager.com/pone/ and select the 'Submissions Needing Revision' folder to locate your manuscript file.

- A letter that responds to each point raised by the academic editor and reviewer(s). You should upload this letter as a separate file labeled 'Response to Reviewers'.
- A marked-up copy of your manuscript that highlights changes made to the original version. You should upload this as a separate file labeled 'Revised Manuscript with Track Changes'.
- An unmarked version of your revised paper without tracked changes. You should upload this as a separate file labeled 'Manuscript'.

If you would like to make changes to your financial disclosure, please include your updated statement in your cover letter. Guidelines for resubmitting your figure files are available below the reviewer comments at the end of this letter.

If applicable, we recommend that you deposit your laboratory protocols in protocols.io to enhance the reproducibility of your results. Protocols.io assigns your protocol its own identifier (DOI) so that it can be cited independently in the future. For instructions see: https://journals.plos.org/plosone/s/submission-guidelines#loc-laboratory-protocols. Additionally, PLOS ONE offers an option for publishing peer-reviewed Lab Protocol articles, which describe protocols hosted on protocols.io. Read more information on sharing protocols at https://plos.org/protocols?utm_medium=editorial-email&utm_source=authorletters&utm_campaign=protocols.

We look forward to receiving your revised manuscript.

Kind regards,

Mario Treviño Villegas, Ph.D

Academic Editor

PLOS One

Journal Requirements:

When submitting your revision, we need you to address these additional requirements.

1. Please ensure that your manuscript meets PLOS ONE's style requirements, including those for file naming. The PLOS ONE style templates can be found at

https://journals.plos.org/plosone/s/file?id=wjVg/PLOSOne_formatting_sample_main_body.pdf and

https://journals.plos.org/plosone/s/file?id=ba62/PLOSOne_formatting_sample_title_authors_affiliations.pdf

2. Please note that PLOS One has specific guidelines on code sharing for submissions in which author-generated code underpins the findings in the manuscript. In these cases, we expect all author-generated code to be made available without restrictions upon publication of the work. Please review our guidelines at https://journals.plos.org/plosone/s/materials-and-software-sharing#loc-sharing-code and ensure that your code is shared in a way that follows best practice and facilitates reproducibility and reuse.

3.  Please update your submission to use the PLOS LaTeX template. The template and more information on our requirements for LaTeX submissions can be found at http://journals.plos.org/plosone/s/latex.

4. Thank you for stating in your Funding Statement:

“This work was supported by the MEXT KAKENHI (Grant Numbers 22K21353, 25H01011 to M.Y. and 22H05158 to A.I-I.) and AMED (Grant Numbers JP24zf0127010 to M.Y).”

Please provide an amended statement that declares *all* the funding or sources of support (whether external or internal to your organization) received during this study, as detailed online in our guide for authors at http://journals.plos.org/plosone/s/submit-now. Please also include the statement “There was no additional external funding received for this study.” in your updated Funding Statement.

Please include your amended Funding Statement within your cover letter. We will change the online submission form on your behalf.

5. Thank you for stating the following financial disclosure:

“This work was supported by the MEXT KAKENHI (Grant Numbers 22K21353, 25H01011 to M.Y. and 22H05158 to A.I-I.) and AMED (Grant Numbers JP24zf0127010 to M.Y).”

Please state what role the funders took in the study. If the funders had no role, please state: "The funders had no role in study design, data collection and analysis, decision to publish, or preparation of the manuscript."

If this statement is not correct you must amend it as needed.

Please include this amended Role of Funder statement in your cover letter; we will change the online submission form on your behalf.

6. Thank you for stating the following in the Acknowledgments Section of your manuscript:

“We extend our gratitude to Drs. Takashi Saito, Takaomi C. Saido, and Yoichiro Abe for generously providing us with AppNL-G-F mice. This work was supported by the MEXT KAKENHI (Grant Numbers 22K21353, 25H01011 to M.Y. and 22H05158 to A.I-I.) and AMED (Grant Numbers JP24zf0127010 to M.Y).”

We note that you have provided funding information that is currently declared in your Funding Statement. However, funding information should not appear in the Acknowledgments section or other areas of your manuscript. We will only publish funding information present in the Funding Statement section of the online submission form.

Please remove any funding-related text from the manuscript and let us know how you would like to update your Funding Statement. Currently, your Funding Statement reads as follows:

“This work was supported by the MEXT KAKENHI (Grant Numbers 22K21353, 25H01011 to M.Y. and 22H05158 to A.I-I.) and AMED (Grant Numbers JP24zf0127010 to M.Y).”

Please include your amended statements within your cover letter; we will change the online submission form on your behalf.

7. Please note that your Data Availability Statement is currently missing [the repository name and/or the DOI/accession number of each dataset OR a direct link to access each database]. If your manuscript is accepted for publication, you will be asked to provide these details on a very short timeline. We therefore suggest that you provide this information now, though we will not hold up the peer review process if you are unable.

8. We note that Figures 1, 2, 3, 4, S5, S11, S12 and S13 in your submission contain copyrighted images. All PLOS content is published under the Creative Commons Attribution License (CC BY 4.0), which means that the manuscript, images, and Supporting Information files will be freely available online, and any third party is permitted to access, download, copy, distribute, and use these materials in any way, even commercially, with proper attribution. For more information, see our copyright guidelines: http://journals.plos.org/plosone/s/licenses-and-copyright.

We require you to either (1) present written permission from the copyright holder to publish these figures specifically under the CC BY 4.0 license, or (2) remove the figures from your submission:

a. You may seek permission from the original copyright holder of Figures 1, 2, 3, 4, S5, S11, S12 and S13 to publish the content specifically under the CC BY 4.0 license.

We recommend that you contact the original copyright holder with the Content Permission Form (http://journals.plos.org/plosone/s/file?id=7c09/content-permission-form.pdf) and the following text:

“I request permission for the open-access journal PLOS ONE to publish XXX under the Creative Commons Attribution License (CCAL) CC BY 4.0 (http://creativecommons.org/licenses/by/4.0/). Please be aware that this license allows unrestricted use and distribution, even commercially, by third parties. Please reply and provide explicit written permission to publish XXX under a CC BY license and complete the attached form.”

Please upload the completed Content Permission Form or other proof of granted permissions as an "Other" file with your submission.

In the figure caption of the copyrighted figure, please include the following text: “Reprinted from [ref] under a CC BY license, with permission from [name of publisher], original copyright [original copyright year].”

b. If you are unable to obtain permission from the original copyright holder to publish these figures under the CC BY 4.0 license or if the copyright holder’s requirements are incompatible with the CC BY 4.0 license, please either i) remove the figure or ii) supply a replacement figure that complies with the CC BY 4.0 license. Please check copyright information on all replacement figures and update the figure caption with source information. If applicable, please specify in the figure caption text when a figure is similar but not identical to the original image and is therefore for illustrative purposes only.

9. Please include captions for your Supporting Information files at the end of your manuscript, and update any in-text citations to match accordingly. Please see our Supporting Information guidelines for more information: http://journals.plos.org/plosone/s/supporting-information.

10. If the reviewer comments include a recommendation to cite specific previously published works, please review and evaluate these publications to determine whether they are relevant and should be cited. There is no requirement to cite these works unless the editor has indicated otherwise.

[Note: HTML markup is below. Please do not edit.]

Reviewers' comments:

Reviewer's Responses to Questions

**Comments to the Author**

1. Is the manuscript technically sound, and do the data support the conclusions?

Reviewer #1: Yes

Reviewer #2: Partly

**********

2. Has the statistical analysis been performed appropriately and rigorously?

Reviewer #1: Yes

Reviewer #2: No

**********

3. Have the authors made all data underlying the findings in their manuscript fully available?

The PLOS Data policy

Reviewer #1: Yes

Reviewer #2: No

**********

4. Is the manuscript presented in an intelligible fashion and written in standard English?

Reviewer #1: Yes

Reviewer #2: Yes

**********

Reviewer #1: This is an excellent manuscript describing “Operant House,” a flexible and low-cost platform for automated operant conditioning in individually housed mice.

I appreciate the versatility, the clear documentation, and the fact that several different cognitive tasks can be conducted without handling or transferring animals.

This approach is likely to reduce stress, increase throughput, and broaden accessibility for labs without specialized behavioral facilities.

The manuscript is written clearly, with extensive methodological detail suitable for replication. Parts lists and the tailored website will help to reproduce the system.

Statistical analyses are appropriate, the validation experiments are convincing, and the proof-of-concept AD model data (now in the supplementary information) is presented in a balanced way. It is also evident that the manuscript has significantly benefited from previous review rounds.

I did not identify any major flaws or substantive weaknesses. A few very minor typographical or formatting inconsistencies remain, but these do not detract from the science. Overall, this work represents a valuable contribution to the field of automated behavioral testing and will be of interest to groups studying cognition, neuropsychiatric disease models, and high-throughput behavioral pipelines.

In my opinion, the manuscript is suitable for publication in its current form, and I encourage acceptance.

Minor inconsistencies:

Typo in the abstract: “within in their home cages - ”duplicated “in”.

Acronym: PL/IL vs PI/IL not consistent

Reviewer #2: Dear Authors,

The manuscript describes a new behavioral setup which combines housing and an operant learning chamber using LCD screens (giving CS), nose poke through a wall with openings (CR),water delivery (US, reward) and LED lights (US, punishment). The authors shows that different behavior protocols can be performed in this setup. I think the work is interesting and this could be a ground work for interesting future development, but the manuscript needs some additional work to optimize the dissemination of the tool and ideas.

I wrote my comments before seeing that it had already be reviewed (I modified it slightly afterwards). I got to similar conclusions that earlier reviewers: both the setup description and the AD-related experiments (so good to see acceptable control group in a AD experiment !) are interesting but should be in two different papers. This will also make the review much easier, and more importantly for the authors and the community: it would make the AD experiments much more visible.

I disagree with the comment to put the setup description in the material and methods, but agree with most other comments. I would also not ask for additional experiments, but in absence of a direct comparison of this setup against a previously published one, with experiments done in parallel, the discussion should be a bit more convincing.

An additional comment compared to earlier review that is essential: the ethical aspects of the setup are critical. In the setup, animal are housed in isolation, with food in the bedding for weeks, this will not be possible in Europe and the authors need to discuss both the stressful effect on their experiments, and ways to change the protocol.

NB : Directive 2010/63/EU (Council of the Europe Union 2010): “Animals, except those which are naturally solitary, shall be socially housed in stable groups of compatible individuals. In cases where single housing is allowed in accordance with article 33(3) the duration shall be limited to the minimum period necessary and visual, auditory, olfactory and/or tactile contact shall be maintained.” This can be kept in the discussion for now, but protocol adjustment to minimise single housing should be a objective for future work.

The authors needs to convince me a bit more that the “Operant House not only reproduces behavioral performance obtained in conventional non–home-cage apparatuses but also enhances experimental efficiency by shortening the overall duration of behavioral paradigms.“. I would like a bit more focus on that aspect (while minimizing single housing). This would be needed to really convince people to use the setup. They only discuss two other papers doing similar experiments without mentioning mice genetic background or age. This would need a more systematic description and comparison.

The second part of the paper about the AppNL-G-F mice should be moved to another paper, I think. I encourage the authors to make two companion paper : one about the new protocol and setup, one about the AD related experiment. I was pleased to see AD experiments where the control group makes sense, thank you for that. I did not review the non-behavioral part of the manuscript as it is out of my expertise and I believe it deserves its own paper.

Major point : Open source and open data aspects

Please indicate license of the software and hardware if you want to call it "open source".

Instruction and data should be provided in a long term archived version, websites tend to disappear. Please use a data repository to host the information for the long term (zenodo for example). I would also encourage the author to move the description into a community friendly tool with version control, like Codeberg or Gitlab, or a similar tool, so people can give feedback there. (This should be very easy as the current documentation on the website looks like a markdown based wiki)

As helper for open hardware (please only use if you think it can help you, not because I mention it), you may have a look at the OSHWA certification program for open hardware (https://certification.oshwa.org/) and the hardware documentation guide I put together (https://open-make.github.io/Hardware-template-guide/).

You mentioned data is available, but it is not. Please give access to all csv files and the analysis code (you can use zenodo for that too).

-----

Minor points :

The first part of the papers sounds too much like an advertisement, as if Fig.1 A. In particular, you should mention the disadvantage of an home cage setup early on and discuss it (abstract and introduction) : single housing, no involvement of the human who could detect stress,...

The electrophysiology and immunstaining experiments are not mentioned in the abstract, title or introduction. Authors should either rewrite those or take out these experiments out of the paper and keep it for another paper more related to the biology experiments (see main comment).

Please put the material and method section before the result section. Indicate the condition of housing first, indicate when light was turned on/off and indicate the number of animal per cage, and size of the cage before the experiment started.

Explain better what light conditions and water restriction were used during the operant house. Make a better figure of the habituation time that includes access to water and link it in the text, I could not follow: do the mice have no water during the first 12h ?

Please explain protocolse without referring to other protocols. You may tell differences after the initial description but this is more for the result/discussion section than the material and method. (I do not understand "the stimulus water supply was not provided." )

Add a table for version 1 and version 2 differences in protocols.

"To prevent the mice from acclimating to a specific pattern of changes, two delay schedules, A and B, were randomly assigned to each trial." Please explain what schedules is it, and what do you mean with "randomly assigned": each mouse got a different series, did you use a randomisation table, was randomisation computer generated ?

Please indicate the number of animals and number of cohorts for the experiments.

Results

Explain why did you choose midnight as the start of the operant time, this is not when the animals are most active ?

Give a better image of the masking wall and refer to the image in the text, also indicate the material used for the wall.

"accuracy 100% (comparison between automated detection and human counting during the first 30 min of the VD2

session)" please gives us data, was it counted by 2 humans ? If not, please rephrase.

"Implemented operant tasks" this list is longer than what is presented in the paper. Maybe add these other tasks in this paper if you split the manuscript.

"Water intake: 25-35 µl per lick." how was this calculated ?

longer actual delay time: it is not clear why this was specified in the AD experiments and not before. This should be part of the description of the behavior and not come at an extra element with the experiment with transgenic animals. “we selected trials whose actual delay time was within 1.3 times the specified delay time plus 10 seconds“: this is part of the material and method section, explain why that selection, and when did you make it.

“Although not statistically significant (genotype: F1,10 = 1.117, p = 0.315), AppNL-G-F mice tended to have longer actual delay times compared to wild-type mice (Fig 7G)”: if this is not significant, there is no reason to mention it.

Discussion:

Please discuss the results, not putative expansion of the system you do not have or show data of (even when this is asked by other reviewers :) ).

“quantitative analysis of posture dynamics“: either show you can do it, or do not mention it. I would guess that a camera on top is not sufficient to do that.

“Additionally, the software includes a TTL signal output and timestamp functionality, enabling seamless integration with external devices for experiments involving optogenetics. “ : please indicate time precision in ms if you want to discuss this.

Please compare habituation time in hours too (in your setup 1 day is 24 hours, what is in Clelland et al ?)

Statistics:

Please give more information on statistics:

The representation and analysis of the data suppose the data is normally distributed, while it probably is not, at least it cannot be for 9A and 9B (as you may have mice never reaching the criteria). Please use boxplots or jitter points, and other tests (unless you can prove the data is normally distributed). Indicate how scores are calculated and give sample sizes (number of mice, number of trials): it should be one score per mice (not one score per trial), but this is not explained.

You also often test the data multiple times without mentioning having a bonferroni correction. Be a bit more straight on your tests: for each experiment have one statistical test that matters first, and for any other test, you may not mention any statistics. Selecting part of the data for the analysis a posteriori is a p-hacking strategy.

**********

what does this mean?). If published, this will include your full peer review and any attached files.

If you choose “no”, your identity will remain anonymous but your review may still be made public.

**Do you want your identity to be public for this peer review?**  For information about this choice, including consent withdrawal, please see our Privacy Policy

Reviewer #1: No

Reviewer #2: **Yes:**  Colomb, Julien

**********

[NOTE: If reviewer comments were submitted as an attachment file, they will be attached to this email and accessible via the submission site. Please log into your account, locate the manuscript record, and check for the action link "View Attachments". If this link does not appear, there are no attachment files.]

To ensure your figures meet our technical requirements, please review our figure guidelines: https://journals.plos.org/plosone/s/figures

You may also use PLOS’s free figure tool, NAAS, to help you prepare publication quality figures: https://journals.plos.org/plosone/s/figures#loc-tools-for-figure-preparation.

NAAS will assess whether your figures meet our technical requirements by comparing each figure against our figure specifications.

# Author comment: Author response to Decision Letter 1

**DOI:** 10.1371/journal.pone.0360626.r003

11 Jun 2026

Point-by-point responses to Reviewers’ comments

We thank the reviewers for their time and constructive feedback. We are encouraged by their positive assessment of our manuscript and by the indication that it will be suitable for publication once the remaining issues are addressed. Below, we provide detailed, point-by-point responses to each comment. Newly added text in the revised manuscript is shown in blue in the marked-up copy.

Reviewer #1:

“This is an excellent manuscript describing “Operant House,” a flexible and low-cost platform for automated operant conditioning in individually housed mice.

I appreciate the versatility, the clear documentation, and the fact that several different cognitive tasks can be conducted without handling or transferring animals.

This approach is likely to reduce stress, increase throughput, and broaden accessibility for labs without specialized behavioral facilities.

The manuscript is written clearly, with extensive methodological detail suitable for replication. Parts lists and the tailored website will help to reproduce the system.

Statistical analyses are appropriate, the validation experiments are convincing, and the proof-of-concept AD model data (now in the supplementary information) is presented in a balanced way. It is also evident that the manuscript has significantly benefited from previous review rounds.

I did not identify any major flaws or substantive weaknesses. A few very minor typographical or formatting inconsistencies remain, but these do not detract from the science. Overall, this work represents a valuable contribution to the field of automated behavioral testing and will be of interest to groups studying cognition, neuropsychiatric disease models, and high-throughput behavioral pipelines.

In my opinion, the manuscript is suitable for publication in its current form, and I encourage acceptance.”

We greatly appreciate these positive and encouraging comments.

Minor inconsistencies:

“Typo in the abstract: “within in their home cages-duplicated `in’.”

“Acronym: PL/IL vs PI/IL not consistent.”

We have corrected these typographical and formatting inconsistencies in the revised manuscript.

Reviewer #2:

“The manuscript describes a new behavioral setup which combines housing and an operant learning chamber using LCD screens (giving CS), nose poke through a wall with openings (CR), water delivery (US, reward) and LED lights (US, punishment). The authors shows that different behavior protocols can be performed in this setup. I think the work is interesting and this could be a ground work for interesting future development, but the manuscript needs some additional work to optimize the dissemination of the tool and ideas.”

“I wrote my comments before seeing that it had already be reviewed (I modified it slightly afterwards). I got to similar conclusions that earlier reviewers: both the setup description and the AD-related experiments (so good to see acceptable control group in a AD experiment!) are interesting but should be in two different papers. This will also make the review much easier, and more importantly for the authors and the community: it would make the AD experiments much more visible.”

“I disagree with the comment to put the setup description in the material and methods, but agree with most other comments...”

We thank Reviewer #2 for the positive assessment of our study and for appreciating not only the Operant House system itself but also the analyses using the Alzheimer’s disease model. We agree that the Alzheimer’s disease-related results are of interest in their own right. However, we would respectfully prefer to retain these data in the present manuscript, because we believe that inclusion of the behavioral analyses in the Alzheimer’s disease model is important for demonstrating the utility of the Operant House in a biologically and medically relevant context.

In the previous round of revision, one reviewer also commented that the manuscript was somewhat difficult to follow because it combined the development and design of the Operant House with the analyses of the Alzheimer’s disease model. In response to that concern, in the revised manuscript we moved sections not directly related to the development and design of the Operant House, such as the electrophysiological and immunohistochemical analyses in the Alzheimer’s disease model, to the Supplemental Information. At the same time, we substantially expanded the descriptions related to the development and design of the Operant House in the main text, figures, and supplementary materials. We hope that these revisions have made the main focus of the manuscript clearer and improved its overall readability.

We believe that retaining the behavioral analyses of the Alzheimer’s disease model strengthens the manuscript by providing a concrete demonstration of the usefulness of the Operant House. For this reason, we feel that presenting both the methodological advance and its application within a single manuscript provides a stronger and more informative account of the work.

Because Reviewer #1 did not raise the concern that the manuscript should be divided into two separate papers, we would respectfully leave the final decision on this point to the Editor.

“…I would also not ask for additional experiments, but in absence of a direct comparison of this setup against a previously published one, with experiments done in parallel, the discussion should be a bit more convincing.”

We thank the reviewer for this helpful suggestion. In response, we have added a new section to the revised main text addressing differences in performance between the Operant House and conventional operant chambers. Specifically, for the lever-press task, we have added a description of how the results shown in Fig S13 compare with those reported in previous studies, in order to better clarify the characteristics and performance of the present system.

“An additional comment compared to earlier review that is essential: the ethical aspects of the setup are critical. In the setup, animal are housed in isolation, with food in the bedding for weeks, this will not be possible in Europe and the authors need to discuss both the stressful effect on their experiments, and ways to change the protocol.

NB : Directive 2010/63/EU (Council of the Europe Union 2010): “Animals, except those which are naturally solitary, shall be socially housed in stable groups of compatible individuals. In cases where single housing is allowed in accordance with article 33(3) the duration shall be limited to the minimum period necessary and visual, auditory, olfactory and/or tactile contact shall be maintained.” This can be kept in the discussion for now, but protocol adjustment to minimise single housing should be a objective for future work.”

We thank the reviewer for raising this essential point. We agree that single housing is an important welfare issue and should be scientifically justified, limited to the minimum duration necessary, and refined where possible. In the present study, mice were singly housed to allow individual behavioral testing without interference from cage mates. We acknowledge that prolonged single housing may affect stress and behavior, and we have added this as a limitation and welfare consideration in the revised Discussion. We also state that minimizing the duration of single housing and preserving sensory contact where feasible should be important goals for future refinement of the Operant House protocol.

“The authors need to convince me a bit more that the “Operant House not only reproduces behavioral performance obtained in conventional non–home-cage apparatuses but also enhances experimental efficiency by shortening the overall duration of behavioral paradigms.” I would like a bit more focus on that aspect (while minimizing single housing). This would be needed to really convince people to use the setup. They only discuss two other papers doing similar experiments without mentioning mice genetic background or age. This would need a more systematic description and comparison.

Following Reviewer #2’s suggestion, we have expanded the revised manuscript to include a more systematic comparison with four previous studies using conventional non-home-cage operant conditioning apparatuses. Where available, we now include information such as sex, age, and genetic background in order to make the comparison with the present study clearer and more informative.

“The second part of the paper about the AppNL-G-F mice should be moved to another paper, I think. I encourage the authors to make two companion papers: one about the new protocol and setup, one about the AD related experiment. I was pleased to see AD experiments where the control group makes sense, thank you for that. I did not review the non-behavioral part of the manuscript as it is out of my expertise, and I believe it deserves its own paper.”

As discussed above, although we greatly appreciate Reviewer #2’s positive assessment of both the Operant House system itself and the analyses using the Alzheimer’s disease model, we would respectfully prefer to retain these data in the present manuscript.

“Major point: Open source and open data aspects

Please indicate license of the software and hardware if you want to call it "open source."

We thank the reviewer for this important comment. The Operant House will be released under the MIT License, and we have now clearly indicated this in the main source code repository and on the Operant House website.

“Instruction and data should be provided in a long-term archived version; websites tend to disappear. Please use a data repository to host the information for the long term (zenodo for example). I would also encourage the author to move the description into a community friendly tool with version control, like Codeberg or Gitlab, or a similar tool, so people can give feedback there. (This should be very easy as the current documentation on the website looks like a markdown-based wiki).”

We thank the reviewer for this helpful advice. Because we have been using GitHub for development and version control, we have now moved the Operant House documentation site from a private server to GitHub Pages, which we believe provides a more stable, transparent, and community-friendly platform for long-term access and user feedback.

“As helper for open hardware (please only use if you think it can help you, not because I mention it), you may have a look at the OSHWA certification program for open hardware (https://certification.oshwa.org/) and the hardware documentation guide I put together (https://open-make.github.io/Hardware-template-guide/).”

We thank the reviewer for introducing us to OSHWA and for sharing these helpful resources. We believe that the philosophy of the OSHWA certification program, particularly its support for researcher-led open-hardware development in academia, closely aligns with the goals of the Operant House project. Accordingly, we have submitted the Operant House project for OSHWA certification.

“You mentioned data is available, but it is not. Please give access to all csv files and the analysis code (you can use zenodo for that too).”

In response, we have now made the relevant CSV files, analysis code, and associated videos publicly available in a GitHub repository, and we have added the corresponding link to the revised manuscript. The repository URL is as follows:

https://github.com/Shintaro18/DataForOperantHousePaper_PlosOne

Minor points:

“The first part of the papers sounds too much like an advertisement, as if Fig.1 A. In particular, you should mention the disadvantage of a home cage setup early on and discuss it (abstract and introduction): single housing, no involvement of the human who could detect stress,...”

We thank Reviewer #2 for this important comment. We understand the concern that the early part of the manuscript may have overemphasized the advantages of the Operant House without sufficiently acknowledging the limitations and practical considerations associated with home-cage behavioral testing. In response, we have revised the Abstract, Introduction, and Discussion to provide a more balanced description of the system.

Specifically, we now state earlier in the manuscript that, although home-cage operant testing offers advantages such as reduced handling and improved experimental efficiency, it also requires careful consideration of animal welfare, particularly with respect to individual housing and the potential effects of social isolation. We have also clarified that the Operant House enables live monitoring of the entire chamber and retrospective inspection of recorded task videos, which facilitate assessment of the animals’ health status and possible signs of stress during experiments. In addition, we have expanded the Discussion to more explicitly address these limitations and the importance of further refinement to minimize the burden associated with single housing.

“The electrophysiology and immunostaining experiments are not mentioned in the abstract, title or introduction. Authors should either rewrite those or take out these experiments out of the paper and keep it for another paper more related to the biology experiments (see main comment).”

In response, we have added a description of the electrophysiological and immunohistochemical analyses to the Abstract.

“Please put the material and method section before the result section.”

Following the reviewer’s suggestion, we have moved the Materials and Methods section to precede the Results section.

“Indicate the condition of housing first, indicate when light was turned on/off and indicate the number of animals per cage, and size of the cage before the experiment started.”

The light on/off schedule is described in the final part of the section entitled “Automated Operant Conditioning Using the Operant House.” We have added the other requested information to the Materials and Methods section of the revised manuscript, including the housing conditions prior to the experiment, the number of animals per cage, and cage size.

“Explain better what light conditions and water restriction were used during the operant house. Make a better figure of the habituation time that includes access to water and link it in the text, I could not follow: do the mice have no water during the first 12h?

Please explain protocols without referring to other protocols. You may tell differences after the initial description, but this is more for the result/discussion section than the material and method.”

In response, we have added a clearer description of the habituation procedure, light conditions, and water restriction schedule to the Materials and Methods section of the revised manuscript. We have also added the water restriction schedule to Figs. 5A and 8A.

“I do not understand ‘the stimulus water supply was not provided’”.

We thank the reviewer for pointing this out. “Stimulus water supply” referred to the “stimulus reward” shown in Figs. 5B and 8B. Because this terminology was inconsistent in the original manuscript, we have now unified it throughout the revised manuscript as “stimulus reward.”

“Add a table for version 1 and version 2 differences in protocols.”

In response, we have included in Fig. S3 of the revised manuscript a table summarizing the differences in the protocols between Operant House Version 1 and Version 2.

"To prevent the mice from acclimating to a specific pattern of changes, two delay schedules, A and B, were randomly assigned to each trial." Please explain what schedules is it, and what do you mean with "randomly assigned": each mouse got a different series, did you use a randomisation table, was randomisation computer generated?”

We thank the reviewer for pointing out that this description was unclear. In the working memory task, two different delay durations were used within a given session; for example, on day 4 of the working memory phase, the delay was either 5 s or 8 s on each trial. The sequence of these delays was determined by a computer-generated randomization table based on a specified seed value. To clarify this procedure, we have added a concrete example of the delay pattern used on day 4 of the working memory phase to the right side of Fig. 5F.

Because the Operant House allows the seed value to be specified, the same delay sequence can be reproduced exactly when the same seed is used. In the present experiments, all mice were tested using the same seed value on each day and therefore experienced identical delay patterns. The seed values used were as follows: day 1, 1; day 2, 2; day 3, 3; day 4, 4; day 5, 5; day 6, 6; day 7, 7; and day 8, 8. We have also added an explanation of this point to the Materials and Methods section of the revised manuscript.

“Please indicate the number of animals and number of cohorts for the experiments.”

The number of animals is indicated in the figure legends, and we have also clarified in the revised manuscript that all experiments were conducted in a single cohort.

“Results. Explain why did you choose midnight as the start of the operant time, this is not when the animals are most active?”

Because mice are nocturnal and show high levels of activity during the dark phase, we initiated the task at midnight, which corresponds to 3 h after the onset of the dark phase, in order to ensure that all animals were reliably awake when the session began.

“Give a better image of the masking wall and refer to the image in the text, also indicate the material used for the wall.”

In response, we have added to Fig. S11 both an overall photograph of the masking wall and a magnified image showing its surface texture more clearly. We have also added a description of the masking wall, including the material used, in the main text.

"accuracy 100% (comparison between automated detection and human counting during the first 30 min of the VD2 session)" please give us data, was it counted by 2 humans? If not, please rephrase.”

We thank the reviewer for this helpful comment. We apologize that this description was unclear. The comparison was based on manual counting by a single observer, not by two independent observers. In this analysis, the number of nose pokes detected automatically matched the number counted manually during the first 30 min of the VD2 session. To avoid overstating this validation, we have revised the wording in the manuscript accordingly. The corresponding video data have also been added to the Supporting Information as S22 Video.

"Implemented operant tasks" this list is longer than what is presented in the paper. Maybe add these other tasks in this paper if you split the manuscript.”

As discussed above, we would prefer to retain the Alzheimer’s disease model data in the present manuscript. While space limitations do not allow us to present experimental data for all operant tasks that have already been implemented, we believe that listing these successfully implemented tasks is nevertheless useful for illustrating the versatility of the Operant House.

"’Water intake: 25-35 µl per lick.’ how was this calculated?”

The estimated water intake per lick was calculated by dividing the decrease in water-bottle weight measured before and after the task session by the total number of licks recorded during the session. Because the bottle tip contains two metal balls that keep the outlet closed except during licking, we consider the contribution of evaporation to be negligible.

“longer actual delay time: it is not clear why this was specified in the AD experiments and not before. This should be part of the description of the behavior and not come at an extra element with the experiment with transgenic animals. “we selected trials whose actual delay time was within 1.3 times the specified delay time plus 10 seconds”: this is part of the material and method section, explain why that selection, and when did you make it.”

We thank the reviewer for this important comment. As pointed out, control of the actual delay duration was also necessary in the DNMTP experiment using ibotenic acid-treated mice, as in the other DNMTP experiments. In response, we have added a new panel showing the actual delay duration (Fig. 6P), and we have added the performance calculated using only trials that met the actual-delay criterion to Fig. 6O (blue outlines).

During this reanalysis, we also found an error in data processing. Although the Materials and Methods section stated that performance was analyzed using trials assigned to delay A, the previous graph had inadvertently been generated using the average of delay A and delay B. We have now corrected this and replaced the graph with the values obtained from delay A only (Fig. 6O, black outlines).

Following the reviewer’s suggestion, we have also added an explanation of the trial-selection procedure based on actual delay duration to the end of the DNMTP section in the Materials and Methods. The criterion of programmed delay × 1.3 + 10 s was adopted empirically because trials exceeding this threshold often reflected poor task engagement by the animals. This criterion was defined after we noticed, during the AppNL-G-F mouse experiments, that the actual delay duration was longer in the mutant group than in the control group; however, we did not compare multiple alternative thresholds.

“Although not statistically significant (genotype: F1,10 = 1.117, p = 0.315), AppNL-G-F mice tended to have longer actual delay times compared to wild-type mice (Fig 7G)”: if this is not significant, there is no reason to mention it.”

Although the mean actual delay duration in the AppNL-G-F group was nearly threefold longer than that in the wild-type group, the difference was not statistically significant. In accordance with the reviewer’s suggestion, we have removed this statement from the revised manuscript.

“Discussion:

Please discuss the results, not putative expansion of the system you do not have or show data of (even when this is asked by other reviewers :) ).

“quantitative analysis of posture dynamics”: either show you can do it, or do not mention it. I would guess that a camera on top is not sufficient to do that.”

In response, we have included new data in the Supporting Information as S23 Video, demonstrating that the positions of the nose and hindquarters can be tracked using DeepLabCut. On this basis, we retained the corresponding statement in the Discussion.

“Additionally, the software includes a TTL signal output and timestamp functionality, enabling seamless integration with external devices for experiments involving optogenetics.”: please indicate time precision in ms if you want to discuss this.”

We thank the reviewer for this helpful comment. We agree that timing precision should be stated explicitly if TTL output is discussed. Because this value was not measured directly for the complete Operant House system, we have revised the manuscript to clarify that the cited precision is based on previously reported performance of the underlying platform, rather than on direct validation of the full setup in the present study.

“Please compare habituation time in hours too (in your setup 1 day is 24 hours, what is in Clelland et al ?”

Unfortunately, a comparison of habituation time in hours was not possible because Clelland et al. did not report the relevant procedural details. That study stated only that mice were pre-trained through several iterative stages, referring to Morton et al. (Nat. Methods, 2006), which likewise did not provide a detailed description of the duration of the habituation or pre-training sessions.

“Statistics:

Please give more information on statistics:

The representation and analysis of the data suppose the data is normally distributed, while it probably is not, at least it cannot be for 9A and 9B (as you may have mice never reaching the criteria). Please use boxplots or jitter points, and other tests (unless you can prove the data is normally distributed). Indicate how scores are calculated and give sample sizes (number of mice, number of trials): it should be one score per mice (not one score per trial), but this is not explained.”

In Fig. 9, all mice reached the criterion and proceeded to the TCSD task. Because the sample sizes were small, we did not perform formal tests for normality. In response to the reviewer’s suggestion, we therefore changed the statistical analyses for two-group comparisons from the t-test to the Mann–Whitney U test, and from the paired t-test to the Wilcoxon signed-rank test. For the two-way repeated-measures ANOVA, we retained this analysis because it was the most suitable method for assessing the effects of genotype and session in these repeated-measures data.

We have also clarified in the Materials and Methods section (“Data analysis and statistics”) that the scores used for the operant tasks were calculated on a per-session basis, not on a per-trial basis. Thus, each mouse contributed one score per day. The number of mice is indicated in the figure legends, and the numbers of trials are provided in Figs. 7H, 9F, 10G, S14F–J, S15E–H, S17E–H, and S18H.

Regarding data presentation, we added plots of individual data points to the quantitative graphs for the immunohistochemical analyses in Fig. S19. For the operant-task graphs, we retained the current format, as boxplots or jitter plots would become visually crowded given the number of groups and sessions and might reduce clarity. Because the underlying data are publicly available in our GitHub repository, readers interested in the detailed distribution of the data may access them there.

“You also often test the data multiple times without mentioning having a bonferroni correction. Be a bit more straight on your tests: for each experiment have one statistical test that matters first, and for any other test, you may not mention any statistics. Selecting part of the data for the analysis a posteriori is a p-hacking strategy.”

We thank the reviewer for this important comment. In the DNMTP and TCSD tasks, Bonferroni correction was applied when repeated comparisons were performed, and this is stated in both the figure legends and the Materials and Methods section.

Regarding the concern about multiple testing, our a priori hypothesis was specifically that AppNL-G-F mice would show impaired working memory relative to wild-type mice. Accordingly, the key analysis was focused on the working memory phase, whereas the earlier phases primarily served as shaping steps to habituate the mice to the apparatus and train them in the task rules. We therefore do not think it would be appropriate to combine all phases into a single statistical framework, as this would test a different and much broader hypothesis than the one addressed in this study. In addition, because the acquisition phase differed in duration across animals, it was not technically feasible to include all phases from VD1 through the working memory phase in a single repeated-measures ANOVA. We have clarified this rationale in the revised manuscript.

Additional change not directly requested by the reviewers

In Fig. S16, we found that, for one of the mice used in the DNMTP task, the touch-detection sensitivity for panel responses in Operant House version 2 had been lowered during the course of the experiment. We therefore replaced this figure with the corresponding dataset excluding this mouse. This change does not affect the results, interpretation, or conclusions of the manuscript.

Attachment Submitted filename: Response to Reviewer_revision.pdf

# Peer review: Decision Letter 1

**DOI:** 10.1371/journal.pone.0360626.r004

**Authors:** Mario Treviño Villegas

9 Jul 2026

Dear Dr. Yuzaki,

Thank you for submitting your manuscript to PLOS ONE. After careful consideration, we feel that it has merit but does not fully meet PLOS ONE’s publication criteria as it currently stands. Therefore, we invite you to submit a revised version of the manuscript that addresses the points raised during the review process.

**We have now received the reviewers' comments on your manuscript. Based on their evaluations, we invite you to submit a minor revision. Please address each reviewer comment point by point and submit a marked-up version of the revised manuscript highlighting all changes. Once we receive and evaluate your revised submission, we will be able to make a final editorial decision.**

Please submit your revised manuscript by Aug 23 2026 11:59PM. If you will need more time than this to complete your revisions, please reply to this message or contact the journal office at plosone@plos.org. When you're ready to submit your revision, log on to https://www.editorialmanager.com/pone/ and select the 'Submissions Needing Revision' folder to locate your manuscript file.

Please include the following items when submitting your revised manuscript:

- A letter that responds to each point raised by the academic editor and reviewer(s). You should upload this letter as a separate file labeled 'Response to Reviewers'.
- A marked-up copy of your manuscript that highlights changes made to the original version. You should upload this as a separate file labeled 'Revised Manuscript with Track Changes'.
- An unmarked version of your revised paper without tracked changes. You should upload this as a separate file labeled 'Manuscript'.

If you would like to make changes to your financial disclosure, please include your updated statement in your cover letter. Guidelines for resubmitting your figure files are available below the reviewer comments at the end of this letter.

If applicable, we recommend that you deposit your laboratory protocols in protocols.io to enhance the reproducibility of your results. Protocols.io assigns your protocol its own identifier (DOI) so that it can be cited independently in the future. For instructions see: https://journals.plos.org/plosone/s/submission-guidelines#loc-laboratory-protocols. Additionally, PLOS ONE offers an option for publishing peer-reviewed Lab Protocol articles, which describe protocols hosted on protocols.io. Read more information on sharing protocols at https://plos.org/protocols?utm_medium=editorial-email&utm_source=authorletters&utm_campaign=protocols.

As the corresponding author, your ORCID iD is verified in the submission system and will appear in the published article. PLOS supports the use of ORCID, and we encourage all coauthors to register for an ORCID iD and use it as well. Please encourage your coauthors to verify their ORCID iD within the submission system before final acceptance, as unverified ORCID iDs will not appear in the published article. *Only*  the individual author can complete the verification step; PLOS staff *cannot*  verify ORCID iDs on behalf of authors.

We look forward to receiving your revised manuscript.

Kind regards,

Mario Treviño Villegas, Ph.D

Academic Editor

PLOS One

**Journal Requirements:**

1. If the reviewer comments include a recommendation to cite specific previously published works, please review and evaluate these publications to determine whether they are relevant and should be cited. There is no requirement to cite these works unless the editor has indicated otherwise.

2. Please review your reference list to ensure that it is complete and correct. If you have cited papers that have been retracted, please include the rationale for doing so in the manuscript text, or remove these references and replace them with relevant current references. Any changes to the reference list should be mentioned in the rebuttal letter that accompanies your revised manuscript. If you need to cite a retracted article, indicate the article’s retracted status in the References list and also include a citation and full reference for the retraction notice.

[Note: HTML markup is below. Please do not edit.]

Reviewers' comments:

Reviewer's Responses to Questions

**Comments to the Author**

Reviewer #2: (No Response)

Reviewer #3: (No Response)

Reviewer #4: All comments have been addressed

Reviewer #5: All comments have been addressed

**********

2. Is the manuscript technically sound, and do the data support the conclusions?

Reviewer #2: Partly

Reviewer #3: Yes

Reviewer #4: Yes

Reviewer #5: Yes

**********

3. Has the statistical analysis been performed appropriately and rigorously?

Reviewer #2: I Don't Know

Reviewer #3: Yes

Reviewer #4: Yes

Reviewer #5: Yes

**********

4. Have the authors made all data underlying the findings in their manuscript fully available?

The PLOS Data policy

Reviewer #2: No

Reviewer #3: Yes

Reviewer #4: Yes

Reviewer #5: Yes

**********

5. Is the manuscript presented in an intelligible fashion and written in standard English?

Reviewer #2: Yes

Reviewer #3: Yes

Reviewer #4: Yes

Reviewer #5: Yes

**********

**Reviewer #2:**  Dear authors,

The manuscript has been only slightly changed. Please note I did not perform a review of the non-behavioral aspects of this paper. I leave it to the editor to state whether this part was reviewed enough from the other reviewers, as authors do not want to take them out.

Please add a section about data and code availability with correct links, and provide an archive of them (via zenodo or GIN). Please also add these links in the website. I could also not find the MIT licence, please make it more visible.

A lot of “response to the review” did not seem to have made their way into the manuscript, I am puzzled whether this is the latest version. In general, the response given are satisfactory, but I do not see the changes in the manuscript.

One exception where I want to insist: in the discussion, the authors should be focusing on discussing the results and not bragging about what else is possible with the hardware. They may mention other paradigms briefly, but it should not take more than two sentences (it is a whole paragraph). Discussion should not bring new information, but only discuss the results. People interested will look at the website, there is no need to mention this here. (“The data presented in the manuscript must support the conclusions drawn. » : no data in the paper means no need to discuss it).

Please also look at each answer and see why the manuscript does not contain answers you mention (for example discussion of performance compared to other 4 studies: still find only 2, only age of animals was added: please also discuss this: is it the same, is it better, why ? This is not the only example, please revisit).

The data provided is not the raw data and the claim from the authors that new graphs can be made out of may be inaccurate (I do not have time to look into details here). Please provide raw data as you get it out of the hardware, this is important for future users (so they know what to expect) and describe the analysis, that I supposed was made in excel ? This can be made in the github repository. I would also look again at the statistics when I get access to the data.

I put a lot of effort in my review, I expect the authors to do similar effort in modifying the manuscript.

**Reviewer #3:**  This manuscript presents the development of the Operant House, a low-cost and automated home-cage system for operant conditioning in mice. This is a well-designed study that addresses an important challenge in behavioral neuroscience. The system is affordable, flexible, and has the potential to make advanced behavioral testing available to more laboratories. The manuscript is clearly organized, and the revisions have improved several parts of the paper, especially the description of animal welfare and experimental methods. The work is suitable for publication after a few minor issues are addressed.

1. The statistical analyses appear appropriate, but some important details are still missing. The authors should report the sample size for every experiment more consistently in the Results or figure legends. It would also help to explain how normality was assessed before using parametric tests, why outliers were removed with the Smirnov-Grubbs test, and to include effect sizes where possible. These additions would make the statistical analysis more complete.

2. The authors state that the Operant House can support many behavioral tasks. While this is likely true, only the DNMTP and TCSD tasks are fully validated in this study. The Discussion should make a clearer distinction between tasks that have been experimentally tested and those that are expected to work but have not yet been validated.

3. The different findings between female and male AppNL-G-F mice are interesting. The Discussion would benefit from a little more explanation of why these sex differences may exist and whether they could reflect differences in disease progression or task demands.

4. The manuscript discusses the limitations of single housing, which is appreciated. Adding a brief description of body weight monitoring, health checks, or other welfare measures during the experiments would further strengthen this section.

This manuscript presents a useful and well-validated behavioral platform that could be valuable for many neuroscience laboratories. The study is carefully performed, and the main conclusions are supported by the data. The remaining concerns are relatively small and mainly involve improving the reporting of statistical methods, clarifying some methodological choices, and slightly strengthening the Discussion.

**Reviewer #4:**  The authors present an automated operant conditioning testing platform, and the experimental procedures are described in a detailed and accessible manner. However, I have a concern regarding how the relevant prior literature is framed.

The following two studies appear to be directly relevant to the present work:

・PLoS One. 2013 Dec 6;8(12). “A fully automated high-throughput training system for rodents.” PMID: 24349451

・Front Neural Circuits. 2017 Mar 1;11:10. “Automated Operant Conditioning in the Mouse Home Cage.” PMID: 28298887

Although these papers are cited in the Discussion section, I think they should also be introduced in the Introduction. Since readers generally encounter the rationale and background of a research article through the Introduction, directly relevant prior studies should be presented there to clarify what has already been established, what approaches have previously been developed, and what limitations or gaps remain. This would allow the novelty and significance of the present Operant House platform to be evaluated more clearly.

**Reviewer #5:**  Author has addressed all previous comments in a manner that is in my opinion acceptable. Congratulations on the paper and hope the design can be of use for many other labs.

**********

what does this mean?). If published, this will include your full peer review and any attached files.

If you choose “no”, your identity will remain anonymous but your review may still be made public.

**Do you want your identity to be public for this peer review?**  For information about this choice, including consent withdrawal, please see our Privacy Policy

Reviewer #2: **Yes:**  Julien Colomb

Reviewer #3: No

Reviewer #4: No

Reviewer #5: No

**********

[NOTE: If reviewer comments were submitted as an attachment file, they will be attached to this email and accessible via the submission site. Please log into your account, locate the manuscript record, and check for the action link "View Attachments". If this link does not appear, there are no attachment files.]

To ensure your figures meet our technical requirements, please review our figure guidelines: https://journals.plos.org/plosone/s/figures

You may also use PLOS’s free figure tool, NAAS, to help you prepare publication quality figures: https://journals.plos.org/plosone/s/figures#loc-tools-for-figure-preparation.

NAAS will assess whether your figures meet our technical requirements by comparing each figure against our figure specifications.

# Author comment: Author response to Decision Letter 2

**DOI:** 10.1371/journal.pone.0360626.r005

7 Sep 2026

Point-by-point responses to reviewers’ comments

We thank the reviewers for their time and constructive feedback, and we are encouraged that the remaining issues are relatively minor and can be addressed in the present revision. Below, we provide detailed, point-by-point responses to each comment. Newly added text in the revised manuscript is shown in blue in the marked-up copy.

Reviewer #2:

1. “The manuscript has been only slightly changed. Please note I did not perform a review of the non-behavioral aspects of this paper. I leave it to the editor to state whether this part was reviewed enough from the other reviewers, as authors do not want to take them out.

Please add a section about data and code availability with correct links, and provide an archive of them (via zenodo or GIN). Please also add these links in the website. I could also not find the MIT licence, please make it more visible.

A lot of “response to the review” did not seem to have made their way into the manuscript, I am puzzled whether this is the latest version. In general, the response given are satisfactory, but I do not see the changes in the manuscript.”

We thank the reviewer for these comments. We apologize that the extent of the revisions made in response to the previous review was not sufficiently apparent. The revisions were indicated in blue in the marked-up version but were not visible in the clean version, and the distinction between the two files may not have been sufficiently clear. To avoid any further confusion, we have carefully rechecked the current manuscript against every item in our previous point-by-point response and have confirmed that all of the revisions described there are included in the manuscript.

As stated in the previous version of the manuscript, the Python code (OperantHouse1.04.py) was publicly available at https://shintaro18.github.io/office1001.html, together with the files required for 3D printing and the complete parts list. The header of the Python code file, OperantHouse1.04.py, already contained the full MIT License notice. However, we agree that the licensing information was not sufficiently prominent on the website itself. To make it immediately visible, we have now added the statement “Software available on this website is released under the MIT License” to the copyright section of the website and have included a direct link to the MIT License.

In response to the reviewer’s request in the previous review round, the data underlying the study had also been uploaded to the same website. Upon rechecking the website, we found that some files were not accessible as intended because of a technical issue. We sincerely apologize for this problem. We have corrected the issue, tested all download links, and confirmed that the data and associated files are now accessible.

In addition, in accordance with the reviewer’s present request, we have deposited a permanent, versioned archive containing the original raw output files, processed datasets underlying the figures and statistical analyses, Python source code, 3D-printing files, parts list, and associated documentation in Zenodo (doi.org/10.5281/zenodo.22019760). The permanent archive link has also been added to the project website.

Finally, we have added a dedicated “Data and Code Availability” section to the revised manuscript. This section provides direct links to both the project website and the permanent Zenodo archive and clearly specifies the locations of the underlying data, source code, 3D-printing files, parts list, and licensing information.

2. ”One exception where I want to insist: in the discussion, the authors should be focusing on discussing the results and not bragging about what else is possible with the hardware. They may mention other paradigms briefly, but it should not take more than two sentences (it is a whole paragraph).”

We thank the reviewer for this comment. We apologize if the paragraph gave the impression that we intended to promote or overstate the capabilities of the hardware. This was not our intention. The paragraph was originally included to clarify the broader applicability of the system and its distinction from existing approaches in response to comments raised during the earlier review process.

Nevertheless, we agree that the Discussion should focus primarily on the findings of the present study. Accordingly, we have substantially shortened the relevant paragraph in the Discussion to two sentences, as suggested by the reviewer. We have also revised the Results and Discussion to clearly distinguish among tasks that were experimentally demonstrated in the present study, tasks that have been evaluated in a separate study, and other paradigms that have been implemented and technically tested but have not yet undergone systematic behavioral validation in animals.

3. ”Please also look at each answer and see why the manuscript does not contain answers you mention (for example discussion of performance compared to other 4 studies: still find only 2, only age of animals was added: please also discuss this: is it the same, is it better, why ? This is not the only example, please revisit).”

We thank the reviewer for pointing this out. We have carefully re-examined the entire manuscript together with our previous point-by-point response to confirm that each change described in the response is clearly reflected in the manuscript.

Regarding the comparison with four previous studies, all four studies were included in the previously revised manuscript, although we recognize that the relevant comparisons were distributed between the Results and Discussion and may therefore not have been sufficiently easy to identify. Specifically, in the Discussion, we compared performance in the working-memory task conducted in Operant House with that reported for a conventional non-home-cage touchscreen-based delayed non-matching-to-position (DNMTP) task by Kwak et al. (2015). We also compared our results with those obtained using a conventional touchscreen-based location-discrimination task by Clelland et al. (2009), including the available information on the age and sex of the animals. In addition, we discussed the results of a location-discrimination (LD) task performed in a conventional non-home-cage touchscreen apparatus by Saifullah et al. (2020) in relation to performance in the corresponding task in Operant House. Finally, in the Results, we compared acquisition of the lever-press task in Operant House with that reported using a conventional non-home-cage lever-press apparatus by Yu et al. (2009). In accordance with the reviewer’s previous suggestion, we included the age, sex, and genetic background of the animals wherever this information was available. However, we agree that listing these experimental characteristics alone does not make sufficiently clear whether the behavioral performance was comparable to, or more efficient than, that reported previously. We have therefore revised the relevant sections to state more explicitly, for each comparison, whether the final behavioral performance was comparable, whether task acquisition or completion was faster, and which methodological differences limit direct quantitative comparison.

In brief, the revised text now clarifies that Operant House produced behavioral performance broadly comparable to that reported using conventional non-home-cage apparatuses, while shortening task acquisition or preparatory training in some paradigms. At the same time, we have avoided claiming superiority where differences in task design, training criteria, mouse strain, age, sex, or reporting methods preclude a rigorous direct comparison.

To make these comparisons easier to locate, we have consolidated and more clearly signposted the relevant text in the revised manuscript and have explicitly described the paradigm, apparatus, available animal characteristics, training duration, and principal performance measures for the relevant comparisons. We have also reviewed all other responses from the previous round and confirmed that the corresponding revisions are present and clearly identifiable in the marked-up manuscript.

4. “The data provided is not the raw data and the claim from the authors that new graphs can be made out of may be inaccurate (I do not have time to look into details here). Please provide raw data as you get it out of the hardware, this is important for future users (so they know what to expect) and describe the analysis, that I supposed was made in excel? This can be made in the github repository. I would also look again at the statistics when I get access to the data.”

We thank the reviewer for this important comment. In the previous revision, we made available the processed numerical data used to generate the graphs in the manuscript. These files allow the figures to be reproduced and alternative graphs to be generated from the processed values, but we agree that they are not the original files directly produced by the Operant House hardware. We apologize that this distinction was not sufficiently clear.

In response to the reviewer’s request, we have now uploaded the original raw output files generated directly by Operant House for all experiments reported in the manuscript, except for the video files, to both the GitHub repository (https://github.com/Shintaro18/DataForOperantHousePaper_PlosOne) and the permanent Zenodo archive (doi.org/10.5281/zenodo.22019760). These raw files include, for example, the original Touch*.txt and Touch*.csv files generated during behavioral testing.

The Operant House software has been updated several times during the course of this study, and the format of the raw output files consequently differs among software versions. Different file formats therefore require the corresponding analysis program or Excel spreadsheet. To make the relationship between the raw data and the appropriate analysis method clear, we have organized the repository by data format and placed the corresponding analysis program or spreadsheet in the same folder as each set of raw data. We have also added a README file that specifies, for each experiment:

1. the format and contents of the raw output files;

2. the corresponding analysis program or spreadsheet;

3. the manuscript figure and panel to which each dataset corresponds.

For datasets analyzed using the dedicated Windows programs, users can run the corresponding executable file and drag and drop the relevant raw data file, such as a Touch*.txt or Touch*.csv file, into the program. The program filenames and the README clearly indicate which raw-data format is supported by each program. Where Excel was used for subsequent calculations or graph preparation, we have provided the corresponding spreadsheets, including the formulas used to derive the plotted values. We have also expanded the “Data Analysis and statistics” subsection of the revised manuscript to describe this workflow.

The video files generated during behavioral testing were not included in the GitHub repository because each file is typically approximately 300 MB and the complete set is very large. These videos were used for routine monitoring and were not used to derive the quantitative outcomes reported in the manuscript. To show future users the format and quality of the video output, we have provided a representative video file in the permanent archive (doi.org/10.5281/zenodo.22019760).

Finally, we have rechecked the processed datasets against the raw output files and re-examined all statistical analyses. Where appropriate, the statistical methods were revised to account explicitly for repeated measurements, data distribution, and multiple comparisons, as detailed in our responses to Reviewer #3, and effect-size estimates have been added where applicable. The numerical source data underlying each figure, together with the files used for statistical analysis, are now included in the archive. We have also added clearer descriptions of the statistical procedures to the Methods and have provided the software and settings used for each analysis. We hope that these additions will allow the reviewer and future users to inspect the original hardware output and reproduce the data-processing and statistical-analysis workflow.

Reviewer #3:

“This manuscript presents the development of the Operant House, a low-cost and automated home-cage system for operant conditioning in mice. This is a well-designed study that addresses an important challenge in behavioral neuroscience. The system is affordable, flexible, and has the potential to make advanced behavioral testing available to more laboratories. The manuscript is clearly organized, and the revisions have improved several parts of the paper, especially the description of animal welfare and experimental methods. The work is suitable for publication after a few minor issues are addressed.”

1. “The statistical analyses appear appropriate, but some important details are still missing. The authors should report the sample size for every experiment more consistently in the Results or figure legends. It would also help to explain how normality was assessed before using parametric tests, why outliers were removed with the Smirnov-Grubbs test, and to include effect sizes where possible. These additions would make the statistical analysis more complete.”

We thank the reviewer for these helpful suggestions. We have carefully rechecked all experiments and revised the Methods and figure legends to report the number of animals and, where applicable, the number of sections, cells, or recordings contributing to each analysis. For the principal behavioral analyses, the individual animal was treated as the experimental unit, and repeated observations within animals were analyzed using mixed-effects models.

We comprehensively re-examined the statistical analyses to better account for the distribution and repeated-measures structure of the data. Correct/incorrect responses in the operant tasks are now analyzed using generalized linear mixed-effects models (GLMMs) with a binomial error distribution and logit link function, with genotype or treatment condition and session (or other repeated-measure factors, as appropriate) as fixed effects and mouse identity as a random effect. Post hoc comparisons are adjusted using the Holm method, and odds ratios (ORs) are reported as effect-size estimates. Simulation-based model diagnostics were performed to assess residual uniformity, dispersion, and potential outliers.

For continuous outcomes involving repeated measurements, we now use linear mixed-effects models (LMMs), with group, the repeated-measure factor, and their interaction as fixed effects and mouse identity as a random effect. Model assumptions were evaluated after fitting the models by examining residual distributions, including assessment of residual normality using the Shapiro–Wilk test. Where necessary, data were transformed to satisfy model assumptions. Post hoc comparisons were adjusted using the Holm method, and Hedges’ g is reported as an effect-size estimate for pairwise comparisons where applicable.

We also reconsidered our previous use of the Smirnov–Grubbs test for outlier exclusion. In the revised mixed-effects analyses, no observations were excluded solely on the basis of statistical outlier testing; potential outliers were instead evaluated as part of the model-diagnostic procedure. The revised “Data analysis and statistics” subsection now describes these procedures explicitly.

Finally, we clarified the unit of analysis for the supportive histological and electrophysiological experiments in Figs. S19–S21. For Fig. S19, section-level measurements were averaged within each mouse, and the mouse was treated as the experimental unit. For the electrophysiological analyses in Figs. S20 and S21, individual recorded neurons or slice recordings were treated as observational units, and the numbers of both animals and recordings are explicitly reported in the corresponding figure legends. In Fig. S20, Levene’s test was used specifically to assess cell-to-cell variability, which is interpreted as cellular heterogeneity rather than as an animal-level estimate of genotype variance.

Thus, the revised manuscript now reports sample sizes and units of analysis explicitly, uses statistical models appropriate to the data structure, describes model diagnostics and multiple-comparison procedures, and provides effect-size estimates where applicable.

2. “The authors state that the Operant House can support many behavioral tasks. While this is likely true, only the DNMTP and TCSD tasks are fully validated in this study. The Discussion should make a clearer distinction between tasks that have been experimentally tested and those that are expected to work but have not yet been validated.”

We thank the reviewer for this important comment. We agree that the previous wording did not distinguish sufficiently clearly between tasks that have been implemented and technically tested in the Operant House software and tasks that have been evaluated experimentally in animals.

All of the tasks listed in the manuscript have been implemented in the source code available on the project website, and we have confirmed that the corresponding programs operate as intended at the software and hardware levels. However, as the reviewer correctly notes, technical implementation does not by itself constitute behavioral validation.

In the present study, the visual discrimination, lever-press, DNMTP, and TCSD tasks were experimentally demonstrated in mice, with the DNMTP and TCSD tasks being used to characterize cognitive phenotypes in AppNL-G-F mice. During the review of this manuscript, a separate study using the Operant House to perform a trial-unique nonmatching-to-location (TUNL) task was also accepted for publication. Thus, the TUNL task has now been evaluated experimentally in a separate study, although it was not validated as part of the present manuscript.

In accordance with the reviewer’s suggestion, we have revised the Discussion to distinguish explicitly among: (1) tasks experimentally examined in the present study; (2) the TUNL task, which has been evaluated in a separate study; and (3) other tasks that have been implemented and technically tested but have not yet undergone systematic behavioral validation in animals. We have also revised statements that could have implied that all available tasks had been behaviorally validated. The revised text now states clearly that the unvalidated tasks represent potential applications of the system that will require task-specific experimental validation.

3. “The different findings between female and male AppNL-G-F mice are interesting. The Discussion would benefit from a little more explanation of why these sex differences may exist and whether they could reflect differences in disease progression or task demands.”

We thank the reviewer for this thoughtful comment. We agree that the sex-dependent behavioral phenotypes observed in AppNL-G-F mice are interesting and warrant further consideration. In the previous version of the Discussion, we compared our findings with earlier studies reporting sex-dependent differences in the behavioral phenotypes of AppNL-G-F mice and discussed the possibility that the DNMTP and TCSD tasks assess partially distinct cognitive processes.

To address the reviewer’s comment more directly, we have now expanded this discussion to clarify that the observed differences could reflect either sex-dependent trajectories of disease-related circuit dysfunction or differences in the cognitive and behavioral demands of the two tasks. The DNMTP task requires animals to retain and update information across a delay, whereas the TCSD task requires discrimination among spatially distributed visual stimuli and may place different demands on cognitive flexibility and response selection. However, because the present study was not designed to compare neuropathological progression or circuit dysfunction between female and male mice, we cannot distinguish among these possibilities. We have therefore presented these interpretations cautiously and have explicitly identified them as hypotheses requiring further investigation.

4. “The manuscript discusses the limitations of single housing, which is appreciated. Adding a brief description of body weight monitoring, health checks, or other welfare measures during the experiments would further strengthen this section.”

We thank the reviewer for this helpful suggestion. We agree that the procedures used to monitor animal welfare during prolonged individual housing should be described more clearly.

During the experiments, body weight was measured daily, and the general condition of each animal was assessed by visual inspection. The health checks included assessment of appearance and grooming, posture, spontaneous activity, apparent food and water intake, and any signs of injury or distress. Body weight was used as an important objective indicator for detecting possible deterioration in health. Animals showing substantial weight loss, abnormal behavior, or other signs of poor health would have been promptly evaluated and, when appropriate, removed from the experiment in accordance with the predetermined humane-endpoint criteria. No animals met these exclusion or humane-endpoint criteria during the experiments.

We have added these welfare-monitoring procedures to the Materials and Methods. We have also added a brief statement to the Discussion emphasizing that, when Operant House is used under individually housed conditions, regular body-weight measurements and visual health assessments are important for identifying potential welfare problems at an early stage.

5. “This manuscript presents a useful and well-validated behavioral platform that could be valuable for many neuroscience laboratories. The study is carefully performed, and the main conclusions are supported by the data. The remaining concerns are relatively small and mainly involve improving the reporting of statistical methods, clarifying some methodological choices, and slightly strengthening the Discussion.”

We sincerely thank the reviewer for this encouraging assessment of our work. We greatly appreciate the reviewer’s careful and constructive evaluation throughout the review process. The comments have helped us improve the clarity of the statistical reporting, methodological descriptions, and Discussion.

Reviewer #4:

1. “The authors present an automated operant conditioning testing platform, and the experimental procedures are described in a detailed and accessible manner. However, I have a concern regarding how the relevant prior literature is framed.

The following two studies appear to be directly relevant to the present work:

・PLoS One. 2013 Dec 6;8(12). “A fully automated high-throughput training system for rodents.” PMID: 24349451

・Front Neural Circuits. 2017 Mar 1;11:10. “Automated Operant Conditioning in the Mouse Home Cage.” PMID: 28298887

Although these papers are cited in the Discussion section, I think they should also be introduced in the Introduction. Since readers generally encounter the rationale and background of a research article through the Introduction, directly relevant prior studies should be presented there to clarify what has already been established, what approaches have previously been developed, and what limitations or gaps remain. This would allow the novelty and significance of the present Operant House platform to be evaluated more clearly.”

We thank the reviewer for this helpful suggestion. We agree that these two directly relevant studies should be introduced in the Introduction, rather than being discussed only later in the manuscript. Accordingly, we have now cited and described both studies in the revised Introduction. We have also clarified what these earlier automated operant-conditioning systems established, as well as the remaining practical and methodological gaps that motivated the development of Operant House. Specifically, the revised text now places Operant House in the context of previous automated and home-cage-based systems by describing both their important contributions and the practical limitations that may restrict broader adoption or flexibility across behavioral paradigms. We then explain how Operant House was designed to address these remaining needs through its combination of low cost, modular hardware, open-source software, compatibility with multiple behavioral paradigms, and suitability for automated testing in individual home cages. We believe that this revised framing places the contribution of Operant House more clearly in the context of prior work without overstating its novelty.

Reviewer #5:

1. “Author has addressed all previous comments in a manner that is in my opinion acceptable. Congratulations on the paper and hope the design can be of use for many other labs.”

We sincerely thank the reviewer for this encouraging comment and for the constructive suggestions provided during the review process. We are very pleased that the reviewer considers our responses and revisions acceptable, and we share the hope that Operant House will be useful to many laboratories.

Attachment Submitted filename: Point-by-point response_v2.docx

# Peer review: Decision Letter 2

**DOI:** 10.1371/journal.pone.0360626.r006

**Authors:** Mario Treviño Villegas

13 Sep 2026

Dear Dr. Yuzaki,

Thank you for submitting your manuscript to PLOS One. After careful consideration, we feel that it has merit but does not fully meet PLOS One’s publication criteria as it currently stands. Therefore, we invite you to submit a revised version of the manuscript that addresses the points raised during the review process.

One reviewer has raised a few remaining minor points that should be addressed before I can make a final decision. Please consider the following comments carefully in your revision:

1. **TCSD interpretation:**  Please be more cautious when describing TCSD deficits as evidence of working-memory impairment, as the task also involves behavioral flexibility and response selection.
2. **Small lesion groups:**  The lesion experiments include very small sample sizes (n = 3–4 per group). The conclusions regarding the role of the PFC should therefore be presented more cautiously, taking this limitation into account.
3. **Electrophysiology:**  Several neurons were recorded from the same animals, which may raise concerns regarding statistical independence. Please clearly report the number of animals and cells recorded per animal and explain how this issue was addressed in the statistical analyses.
4. **Delay-time selection:**  Selecting trials based on the actual delay achieved may introduce potential selection bias. Please report how many trials were excluded on this basis and discuss this limitation.
5. **Water restriction:**  Because motivation can influence operant performance, please report body-weight and water-intake data, or provide a clearer discussion of these factors and their potential influence on the behavioral results.

Please address these points carefully in the revised manuscript and indicate the corresponding changes clearly.

Please submit your revised manuscript by Oct 28 2026 11:59PM. If you will need more time than this to complete your revisions, please reply to this message or contact the journal office at plosone@plos.org. When you're ready to submit your revision, log on to https://www.editorialmanager.com/pone/ and select the 'Submissions Needing Revision' folder to locate your manuscript file.

- A letter that responds to each point raised by the academic editor and reviewer(s). You should upload this letter as a separate file labeled 'Response to Reviewers'.
- A marked-up copy of your manuscript that highlights changes made to the original version. You should upload this as a separate file labeled 'Revised Manuscript with Track Changes'.
- An unmarked version of your revised paper without tracked changes. You should upload this as a separate file labeled 'Manuscript'.

If you would like to make changes to your financial disclosure, please include your updated statement in your cover letter. Guidelines for resubmitting your figure files are available below the reviewer comments at the end of this letter.

If applicable, we recommend that you deposit your laboratory protocols in protocols.io to enhance the reproducibility of your results. Protocols.io assigns your protocol its own identifier (DOI) so that it can be cited independently in the future. For instructions see: https://journals.plos.org/plosone/s/submission-guidelines#loc-laboratory-protocols. Additionally, PLOS One offers an option for publishing peer-reviewed Lab Protocol articles, which describe protocols hosted on protocols.io. Read more information on sharing protocols at https://plos.org/protocols?utm_medium=editorial-email&utm_source=authorletters&utm_campaign=protocols.

As the corresponding author, your ORCID iD is verified in the submission system and will appear in the published article. PLOS supports the use of ORCID, and we encourage all coauthors to register for an ORCID iD and use it as well. Please encourage your coauthors to verify their ORCID iD within the submission system before final acceptance, as unverified ORCID iDs will not appear in the published article. *Only*  the individual author can complete the verification step; PLOS staff *cannot*  verify ORCID iDs on behalf of authors.

We look forward to receiving your revised manuscript.

Kind regards,

Mario Treviño Villegas, Ph.D

Academic Editor

PLOS One

Journal Requirements:

If the reviewer comments include a recommendation to cite specific previously published works, please review and evaluate these publications to determine whether they are relevant and should be cited. There is no requirement to cite these works unless the editor has indicated otherwise.

Please review your reference list to ensure that it is complete and correct. If you have cited papers that have been retracted, please include the rationale for doing so in the manuscript text, or remove these references and replace them with relevant current references. Any changes to the reference list should be mentioned in the rebuttal letter that accompanies your revised manuscript. If you need to cite a retracted article, indicate the article’s retracted status in the References list and also include a citation and full reference for the retraction notice.

[Note: HTML markup is below. Please do not edit.]

Reviewers' comments:

Reviewer's Responses to Questions

**Comments to the Author**

Reviewer #3: (No Response)

Reviewer #4: All comments have been addressed

**********

2. Is the manuscript technically sound, and do the data support the conclusions?

Reviewer #3: Yes

Reviewer #4: Yes

**********

3. Has the statistical analysis been performed appropriately and rigorously?

Reviewer #3: Yes

Reviewer #4: Yes

**********

4. Have the authors made all data underlying the findings in their manuscript fully available?

The PLOS Data policy

Reviewer #3: Yes

Reviewer #4: Yes

**********

5. Is the manuscript presented in an intelligible fashion and written in standard English?

Reviewer #3: Yes

Reviewer #4: Yes

**********

Reviewer #3: The revised manuscript has improved and most previous comments have been addressed. However, a few important issues remain:

1. TCSD interpretation: The authors should be more cautious in describing TCSD deficits as working-memory impairment, since the task also involves behavioral flexibility and response selection.

2. Small lesion groups: The lesion experiments have very small sample sizes (n=3–4 per group). The conclusions about the role of the PFC should therefore be presented more cautiously.

3. Electrophysiology: Several neurons were recorded from the same animals, which may raise concerns about statistical independence. The authors should clearly report the number of animals and cells per animal and explain how this was handled.

4. Delay-time selection: Selecting trials based on the actual delay achieved may introduce bias. The authors should report how many trials were excluded and discuss this limitation.

5. Water restriction: Since motivation can affect operant performance, body-weight and water-intake data should be reported or discussed more clearly.

Overall, the revision is substantial, but these points should be addressed before publication.

Reviewer #4: The authors have adequately addressed my concern by adding the relevant previous studies to the Introduction and clarifying the position of the present work in relation to these earlier systems. I have no further comments and recommend acceptance of the manuscript.

**********

what does this mean?). If published, this will include your full peer review and any attached files.

If you choose “no”, your identity will remain anonymous but your review may still be made public.

**Do you want your identity to be public for this peer review?**  For information about this choice, including consent withdrawal, please see our Privacy Policy

Reviewer #3: No

Reviewer #4: No

**********

[NOTE: If reviewer comments were submitted as an attachment file, they will be attached to this email and accessible via the submission site. Please log into your account, locate the manuscript record, and check for the action link "View Attachments". If this link does not appear, there are no attachment files.]

To ensure your figures meet our technical requirements, please review our figure guidelines: https://journals.plos.org/plosone/s/figures

You may also use PLOS’s free figure tool, NAAS, to help you prepare publication quality figures: https://journals.plos.org/plosone/s/figures#loc-tools-for-figure-preparation.

NAAS will assess whether your figures meet our technical requirements by comparing each figure against our figure specifications.

# Author comment: Author response to Decision Letter 3

**DOI:** 10.1371/journal.pone.0360626.r007

22 Sep 2026

Point-by-point responses to Reviewers’ comments

We thank the Academic Editor and Reviewers for careful review of our revised manuscript and for identifying the remaining issues. These points correspond to the comments raised by Reviewer #3 and are addressed individually below. We believe that these constructive comments have helped us to improve the clarity of the manuscript and the rigor with which the behavioral and electrophysiological findings are interpreted.

Reviewer #3:

1. TCSD interpretation “The authors should be more cautious in describing TCSD deficits as working-memory impairment, since the task also involves behavioral flexibility and response selection.”

We agree with the Reviewer. We have revised the manuscript throughout to distinguish the interpretation of the DNMTP and TCSD tasks more clearly. The DNMTP task is described as directly assessing working memory, whereas TCSD performance is now described as reflecting multiple cognitive processes, including working-memory-related processes, behavioral flexibility, and response selection.

Accordingly, we have revised the Abstract, Introduction, Results, Discussion, figure titles, and concluding statements where necessary. For example, the Discussion now states:

“The DNMTP task directly assesses working memory, whereas performance in the TCSD task likely reflects multiple cognitive processes, including working memory, behavioral flexibility, and response selection.”

Similarly, the Results now describe the phenotype of male AppNL-G-F mice as “impaired TCSD performance, consistent with deficits in working-memory-related processes and/or behavioral flexibility and response selection,” rather than simply as working-memory impairment. The title of Fig. 10 has also been revised to state that male AppNL-G-F mice exhibit “impaired performance in the TCSD task.”

Finally, the overall conclusion now refers to the detection of “early cognitive deficits” in AppNL-G-F mice, while specifically identifying working-memory impairment as the phenotype detected by the DNMTP task.

2. Small lesion groups ” The lesion experiments have very small sample sizes (n = 3–4 per group). The conclusions about the role of the PFC should therefore be presented more cautiously.”

We agree that the relatively small group sizes in the lesion experiments limit the strength and precision of the conclusions that can be drawn regarding prefrontal cortex involvement.

We have therefore revised the Discussion to state explicitly:

“Because the lesion experiments involved relatively small groups (n = 3–4 per group), these findings should be regarded as supportive evidence for prefrontal involvement and interpreted cautiously with respect to the magnitude of the lesion effects.”

We have also moderated the corresponding language in the Results. For the TCSD experiment, we now describe the lesion phenotype as “impaired TCSD performance” and conclude that the findings “support a contribution of the prefrontal cortex to TCSD performance,” rather than interpreting the lesion effect as definitive evidence of working-memory impairment.

Similarly, for the DNMTP experiment, the delay-restricted analysis is now described as a sensitivity analysis supporting the primary all-trial analysis, rather than as independently confirming a lesion-induced working-memory deficit.

3. Electrophysiology “Several neurons were recorded from the same animals, which may raise concerns about statistical independence. The authors should clearly report the number of animals and cells per animal and explain how this was handled.”

We thank the Reviewer for raising this important statistical issue. We agree that multiple cells or slices obtained from the same animal cannot be regarded as fully independent biological replicates.

We therefore reanalyzed all genotype comparisons in the electrophysiological experiments shown in S20 and S21 using heteroscedastic linear mixed-effects models. Individual cells (S20) or slice recordings (S21) were retained as observational units, while mouse identity was included as a random intercept to account explicitly for the clustering of multiple recordings within the same animal. Genotype was included as a fixed effect, and genotype-specific residual variances were allowed using the varIdent variance structure in the nlme package. Thus, the individual recordings contribute information to the analysis, while their non-independence within each animal is explicitly accounted for.

For S20, the numbers of recordings from individual animals are now reported explicitly in the figure legend. The recording numbers were as follows:

• S20A: wild-type, 22 cells from 3 mice (12, 2, and 8 cells per mouse); AppNL-G-F, 22 cells from 3 mice (11, 6, and 5 cells per mouse).

• S20B, mEPSCs: wild-type, 14 cells from 4 mice (3, 3, 3, and 5 cells per mouse); AppNL-G-F, 22 cells from 3 mice (8, 9, and 5 cells per mouse).

• S20B, mIPSCs: wild-type, 15 cells from 4 mice (3, 4, 3, and 5 cells per mouse); AppNL-G-F, 17 cells from 3 mice (2, 9, and 6 cells per mouse).

• S20C, I/E ratio: 13 cells from 3 mice per genotype (wild-type: 6, 4, and 3 cells per mouse; AppNL-G-F: 3, 5, and 5 cells per mouse).

For the previously reported difference in cell-to-cell variability in the I/E ratio, we additionally compared a homoscedastic model with a heteroscedastic model allowing genotype-specific residual variances. Because residuals from the untransformed I/E-ratio data were non-normally distributed, the data were natural-log transformed before the final analysis. The estimated residual cell-to-cell variability in the I/E ratio was greater in AppNL-G-F mice, and the heteroscedastic model provided a significantly better fit than the homoscedastic model (likelihood-ratio test, χ²(1) = 23.31, p < 0.0001).

We also reanalyzed mIPSC frequency using the mixed-effects framework. After transformation required to satisfy the residual-normality assumption, the previously observed genotype difference in variability was no longer significant. We therefore removed the previous claim of increased mIPSC-frequency variability from the revised manuscript.

For S21, LTP magnitude and paired-pulse ratio were also reanalyzed using heteroscedastic linear mixed-effects models with mouse identity included as a random intercept. For each genotype, 9 slice recordings were obtained from 3 mice, with 3 slices recorded from each mouse. Neither LTP magnitude (F(1, 4) = 0.059, p = 0.821) nor paired-pulse ratio (F(1, 4) = 0.289, p = 0.620) differed significantly between genotypes.

The Methods, Results, and S20/S21 figure legends have been revised accordingly. We have also clarified that individual-cell and individual-slice measurements are retained as observational units, whereas genotype-level statistical inference is performed using mixed-effects models that explicitly account for clustering of multiple recordings within individual animals.

4. Delay-time selection “Selecting trials based on the actual delay achieved may introduce bias. The authors should report how many trials were excluded and discuss this limitation.”

We agree with the Reviewer that restricting analyses according to actual delay duration may introduce selection bias because actual delay duration itself can be influenced by the animal’s behavioral state.

We have therefore revised the manuscript in three ways.

First, we now explicitly distinguish the primary analyses, which include all trials, from the delay-restricted analyses, which are treated as sensitivity analyses. The same delay criterion was applied to all experimental groups and was based solely on actual delay duration, irrespective of whether the trial response was correct or incorrect.

Second, we now report the exact numbers and proportions of retained and excluded trials for each delay-restricted analysis:

• DNMTP lesion experiment (Fig. 6): 1,231 of 1,783 saline trials (69.0%) and 1,309 of 2,013 IBO trials (65.0%) were retained; 552 (31.0%) and 704 (35.0%) trials, respectively, were excluded.

• DNMTP AppNL-G-F experiment (Fig. 7): 2,756 of 3,403 wild-type trials (81.0%) and 2,909 of 3,700 AppNL-G-F trials (78.6%) were retained; 647 (19.0%) and 791 (21.4%) trials, respectively, were excluded.

• TCSD lesion experiment (Fig. 9): 445 of 1,514 saline trials (29.4%) and 210 of 1,398 IBO trials (15.0%) were retained; 1,069 (70.6%) and 1,188 (85.0%) trials, respectively, were excluded.

• TCSD AppNL-G-F experiment (Fig. 10): 3,728 of 5,019 wild-type trials (74.3%) and 2,696 of 4,531 AppNL-G-F trials (59.5%) were retained; 1,291 (25.7%) and 1,835 (40.5%) trials, respectively, were excluded.

Third, we have added an explicit limitation to the Discussion. We note that selection based on actual delay duration could retain different subsets of trials across experimental groups, particularly in the TCSD experiments, in which the proportions of retained trials differed substantially between groups. We therefore interpret the delay-restricted analyses only as sensitivity analyses used to determine whether the principal findings persisted when variability in actual delay duration was reduced; they are not treated as independent confirmatory analyses. The primary conclusions are based on analyses including all trials.

5. Water restriction “Since motivation can affect operant performance, body-weight and water-intake data should be reported or discussed more clearly.”

We agree that motivational state is an important consideration in water-reinforced operant tasks.

We have therefore clarified the water-access protocol and animal monitoring procedures in the Methods. For the DNMTP and TCSD paradigms, cumulative water-access duration was monitored during each daily session. If water-access duration was below the predefined level (DNMTP: 160 s; TCSD: 300 s), supplemental water access was provided immediately after the session. We also clarify that the programmed variable was water-access duration, and that the actual volume of water consumed by each mouse was not directly measured.

Body weight was measured daily as part of routine welfare monitoring during behavioral testing, together with visual assessment of general appearance, grooming, posture, spontaneous activity, apparent food and water intake, and signs of injury or distress.

In addition, we recovered paired body-weight measurements from six wild-type mice tested in independent Operant House version 2 experiments using the same programmed DNMTP water-access regimen (up to 80 water-access periods of 2 s each; maximum programmed access duration, 160 s/day). Mean body weight was 22.47 ± 0.52 g before testing and 21.89 ± 1.11 g after testing, corresponding to no significant group-level change (paired t-test, t(5) = −0.65, p = 0.545). These data have now been added to the Results.

We have also added a limitation to the Discussion. Specifically, because task performance was reinforced by access to water, we acknowledge that differences in motivational state related to thirst cannot be completely excluded. Moreover, because actual water consumption was not directly measured and the available paired body-weight data were obtained from independent experiments rather than from all principal experimental cohorts, individual or genotype-dependent differences in hydration or motivation remain possible.

Reviewer #4: “All comments have been addressed.”

We thank Reviewer #4 for the careful evaluation of our revised manuscript and are grateful that the Reviewer considers the previous concerns to have been adequately addressed. No additional changes were required in response to this comment.

Attachment Submitted filename: Point-by-point response_final.docx

# Peer review: Decision Letter 3

**DOI:** 10.1371/journal.pone.0360626.r008

**Authors:** Mario Treviño Villegas

30 Sep 2026

<p>Operant House: A Versatile Open-Source Platform for Automated Operant Conditioning Testing of Mice in Home Cages

PONE-D-25-65341R3

Dear Dr. Yuzaki,

We’re pleased to inform you that your manuscript has been judged scientifically suitable for publication and will be formally accepted for publication once it meets all outstanding technical requirements.

Within one week, you’ll receive an e-mail detailing the required amendments. When these have been addressed, you’ll receive a formal acceptance letter and your manuscript will be scheduled for publication.

An invoice will be generated when your article is formally accepted. Please note, if your institution has a publishing partnership with PLOS and your article meets the relevant criteria, all or part of your publication costs will be covered. Please make sure your user information is up-to-date by logging into Editorial Manager at Editorial Manager® and clicking the ‘Update My Information' link at the top of the page. For questions related to billing, please contact billing support.

If your institution or institutions have a press office, please notify them about your upcoming paper to help maximize its impact. If they’ll be preparing press materials, please inform our press team as soon as possible -- no later than 48 hours after receiving the formal acceptance. Your manuscript will remain under strict press embargo until 2 pm Eastern Time on the date of publication. For more information, please contact onepress@plos.org.

Kind regards,

Mario Treviño Villegas, Ph.D

Academic Editor

PLOS One

Additional Editor Comments (optional):

Reviewers' comments:

Reviewer's Responses to Questions

**Comments to the Author**

Reviewer #3: All comments have been addressed

**********

2. Is the manuscript technically sound, and do the data support the conclusions?

Reviewer #3: Yes

**********

3. Has the statistical analysis been performed appropriately and rigorously?

Reviewer #3: Yes

**********

4. Have the authors made all data underlying the findings in their manuscript fully available?

The PLOS Data policy

Reviewer #3: Yes

**********

5. Is the manuscript presented in an intelligible fashion and written in standard English?

Reviewer #3: Yes

**********

Reviewer #3: (No Response)

**********

what does this mean?). If published, this will include your full peer review and any attached files.

If you choose “no”, your identity will remain anonymous but your review may still be made public.

**Do you want your identity to be public for this peer review?**  For information about this choice, including consent withdrawal, please see our Privacy Policy

Reviewer #3: No

**********

# Editor report: Acceptance letter

**DOI:** 10.1371/journal.pone.0360626.r009

**Authors:** Mario Treviño Villegas

PONE-D-25-65341R3

PLOS One

Dear Dr. Yuzaki,

I'm pleased to inform you that your manuscript has been deemed suitable for publication in PLOS One. Congratulations! Your manuscript is now being handed over to our production team.

At this stage, our production department will prepare your paper for publication. This includes ensuring the following:

* All references, tables, and figures are properly cited

* All relevant supporting information is included in the manuscript submission,

* There are no issues that prevent the paper from being properly typeset

You will receive further instructions from the production team, including instructions on how to review your proof when it is ready. Please keep in mind that we are working through a large volume of accepted articles, so please give us a few days to review your paper and let you know the next and final steps.

Lastly, if your institution or institutions have a press office, please let them know about your upcoming paper now to help maximize its impact. If they'll be preparing press materials, please inform our press team within the next 48 hours. Your manuscript will remain under strict press embargo until 2 pm Eastern Time on the date of publication. For more information, please contact onepress@plos.org.

You will receive an invoice from PLOS for your publication fee after your manuscript has reached the completed accept phase. If you receive an email requesting payment before acceptance or for any other service, this may be a phishing scheme. Learn how to identify phishing emails and protect your accounts at https://explore.plos.org/phishing.

If we can help with anything else, please email us at customercare@plos.org.

Thank you for submitting your work to PLOS One and supporting open access.

Kind regards,

PLOS One Editorial Office Staff

on behalf of

Dr. Mario Treviño Villegas

Academic Editor

PLOS One
